# Transcriptional fingerprints of antigen-presenting cell subsets in the human vaginal mucosa and skin reflect tissue-specific immune microenvironments

**DOI:** 10.1186/s13073-014-0098-y

**Published:** 2014-11-25

**Authors:** Dorothée Duluc, Romain Banchereau, Julien Gannevat, Luann Thompson-Snipes, Jean-Philippe Blanck, Sandra Zurawski, Gerard Zurawski, Seunghee Hong, Jose Rossello-Urgell, Virginia Pascual, Nicole Baldwin, Jack Stecher, Michael Carley, Muriel Boreham, SangKon Oh

**Affiliations:** Baylor Institute for Immunology Research, 3434 Live Oak St, Dallas, TX 75204 USA; Department of Obstetrics and Gynecology, Baylor University Medical Center, 3600 Gaston Ave, Dallas, TX 75246 USA

## Abstract

**Background:**

Dendritic cells localize throughout the body, where they can sense and capture invading pathogens to induce protective immunity. Hence, harnessing the biology of tissue-resident dendritic cells is fundamental for the rational design of vaccines against pathogens.

**Methods:**

Herein, we characterized the transcriptomes of four antigen-presenting cell subsets from the human vagina (Langerhans cells, CD14^-^ and CD14^+^ dendritic cells, macrophages) by microarray, at both the transcript and network level, and compared them to those of three skin dendritic cell subsets and blood myeloid dendritic cells.

**Results:**

We found that genomic fingerprints of antigen-presenting cells are significantly influenced by the tissue of origin as well as by individual subsets. Nonetheless, CD14^+^ populations from both vagina and skin are geared towards innate immunity and pro-inflammatory responses, whereas CD14^-^ populations, particularly skin and vaginal Langerhans cells, and vaginal CD14^-^ dendritic cells, display both Th2-inducing and regulatory phenotypes. We also identified new phenotypic and functional biomarkers of vaginal antigen-presenting cell subsets.

**Conclusions:**

We provide a transcriptional database of 87 microarray samples spanning eight antigen-presenting cell populations in the human vagina, skin and blood. Altogether, these data provide molecular information that will further help characterize human tissue antigen-presenting cell lineages and their functions. Data from this study can guide the design of mucosal vaccines against sexually transmitted pathogens.

**Electronic supplementary material:**

The online version of this article (doi:10.1186/s13073-014-0098-y) contains supplementary material, which is available to authorized users.

## Background

Dendritic cells (DCs) are professional antigen-presenting cells (APCs) that can induce and direct host immune responses towards immunity or tolerance [1]. DCs disseminate throughout the body, sensing invading pathogens in various tissues, including the skin [2-4] and mucosa [5-7]. Therefore, defining the biology of tissue-resident DCs is fundamental for the understanding of tissue-specific immune microenvironments and for the rational design of vaccines that can mount protective immunity in these tissues.

Sexually transmitted microbial pathogens, including viruses and bacteria [8,9], are a major public health burden worldwide. The human vaginal mucosa is the main entry site of these pathogens and therefore has long been attractive as a potential site for mounting protective mucosal immunity. However, the vaginal mucosa, a site constantly exposed to foreign antigens, is also thought to be a unique tolerogenic microenvironment that tightly regulates unwanted immune responses [10-12]. Nonetheless, the immunology of the human vagina remains poorly understood.

We recently reported the presence of four major subsets of APCs in the human vaginal mucosa, including Langerhans cells (LCs) in the epithelium, and CD14^-^ DCs, CD14^+^ DCs and macrophages (Møs) in the lamina propria (LP) [5,6]. These mucosal APC subsets display common and unique functions in directing T-cell responses *in vitro* [5,6], as do subsets of DCs isolated from human skin [2-4]. Importantly, DCs can display functional specialization and plasticity in response to external and internal stimuli [13,14], which can determine the outcome of host immune responses. Recent evidence further indicates that these characteristics of DCs can be influenced by tissue-specific physical and biological factors [15,16]. One can thus hypothesize that the same DC subsets localized in different tissues could display distinct functions in response to the same antigens. These differences may also influence the type of immunity established in different human anatomical sites. As such, vaccines delivered to skin DCs can elicit systemic immunity but are not sufficient to mount mucosal immunity [6,10,17].

Systems biology approaches provide snapshots of genetic, transcriptional and protein networks, enabling the phenotypic and functional analysis of the immune system [18-20]. In this study, we investigated the phenotype and function of human vaginal DC subsets by microarray transcriptional profiling and compared them with those of DCs from human skin and blood. This study provides fundamental information for the immunology of human vaginal mucosa versus skin, which can eventually guide the rational design of efficacious vaccines against sexually transmitted pathogens.

## Methods

### Samples

Vaginal and skin tissues were obtained from female patients who underwent pelvic or cosmetic surgeries under protocols approved by the Institutional Review Board of Baylor Research Institute (Dallas, TX, USA). Informed consent was waived by the institutional review board (IRB 008-227) for tissue samples. This study conforms to the Helsinki Declaration. Patients were not infected with HIV, hepatitis C virus or tuberculosis and did not display inflammation in the tissues. Written informed consent was obtained from healthy female volunteers to use their blood in this study, and the protocol was approved by the IRB (IRB 012-200) of Baylor Research Institute.

### Vaginal and skin antigen-presenting cell isolation

**Figure 1:**
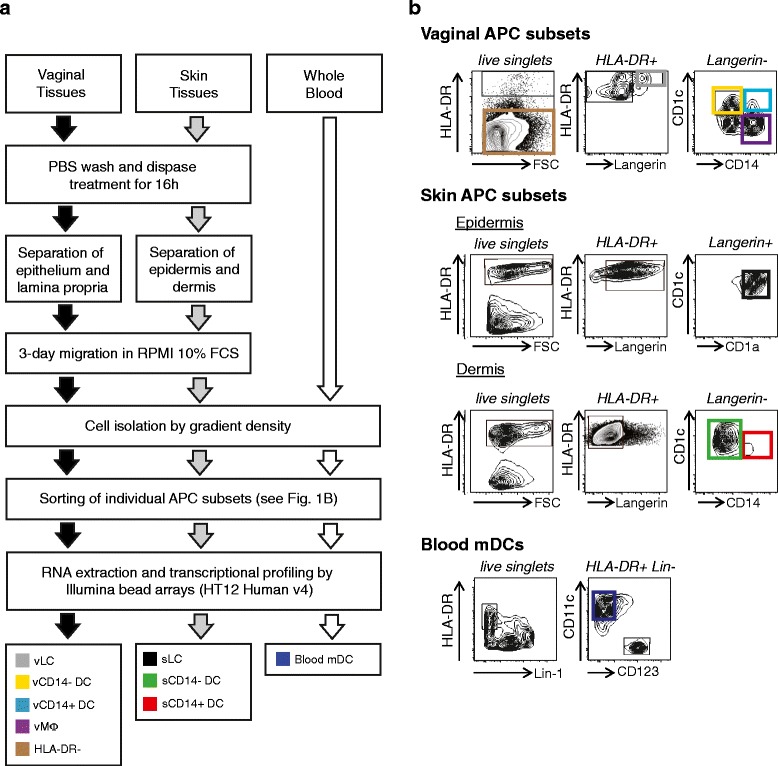
**Isolation protocols for the eight antigen-presenting cell populations studied. (a)** Workflow representing the protocol for isolation of APCs from skin, vagina and blood. **(b)** Gating strategy for FACS sorting of vaginal APC subsets (upper panels), skin DCs (middle panels) or blood mDCs (lower panels). mDC, myeloid dendritic cell; FCS, fetal calf serum; PBS, phosphate-buffered saline; s, skin; v, vaginal.

Tissue biopsies were cut into 1 cm^2^ pieces and incubated in phosphate-buffered saline containing bacterial protease dispase type 2 (Roche Applied Science, Indianapolis, IN, USA) and antibiotic/antimycotic solution (Invitrogen, Carlsbad, CA, USA) overnight at 4°C. Epithelium and LP were then separated. The LP was cut into smaller pieces (1 to 5 mm^2^). Epithelial sheets and LP pieces were incubated at 37°C in RPMI 1640 (Invitrogen) supplemented with HEPES buffer (Invitrogen), antibiotic/antimycotic (Invitrogen), L-glutamine, nonessential amino acids, sodium pyruvate (Sigma Aldrich, St. Louis, MO, USA) and 10% fetal calf serum (HyClone, Logan, UT, USA). After 2 days, the cells that migrated into the medium were further enriched by Ficoll-sodium diatrizoate gradient (Lymphocyte Separation Medium, MP Biomedicals, Solon, OH, USA). Cells were stained with 7-AAD (Biolegend, San Diego, CA, USA), anti-HLA-DR-AF700 (Biolegend), anti-Langerin-PE (Beckman Coulter, Brea, CA, USA), anti-CD1c-FITC (Invitrogen) and CD14-eFluor450 (eBioscience, San Diego, CA, USA). HLA-DR^+^ cells were gated and Langerin^+^ LCs, CD1c^+^CD14^-^ DCs, CD1c^+^CD14^+^ DCs and CD1c^-^ CD14^+^ Møs were sorted by FACS Aria II (BD Biosciences, San Jose, CA, USA) (Figure 1a, b). To purify HLA-DR^-^ cells, single cell suspensions of epithelium and LP were mixed and subsequently sorted (Figure 1b). Skin biopsies were processed similarly. Langerin^+^ cells from the epidermis (sLCs; note that cell types prefixed by 's' refer to skin cells) as well as CD1c^+^CD14^-^ DCs and CD1c^+^CD14^+^ DCs cells from the dermis were sorted by FACS Aria II (BD Biosciences). As previously described [3], sCD14^-^ DCs were CD1a^+^, while sCD14^+^ DCs were CD1a^-^ and sLCs were CD1a^high^ (Additional file 1).

### Blood dendritic cell isolation

Peripheral blood mononuclear cells from healthy volunteers were isolated by density gradient centrifugation using Ficoll-Paque™ PLUS (GE Healthcare, Stockholm, Sweden). Blood DCs were enriched from these cells using a Stemcell Pan-DC kit (Stemcell, Vancouver, BC, Canada) according to the manufacturer’s protocol. Cells were then stained with Lin-FITC (BD Biosciences), CD123-PE (Biolegend), CD11c-APC (BD Biosciences) and HLA-DR-Pacific Orange (Invitrogen). mDC (Lin^-^HLA-DR^+^CD11c^+^CD123^-^) were then sorted by FACS Aria (BD Biosciences) (Figure 1a, b).

### Immunofluorescence and microscopy

Cryo-sections were fixed in cold acetone, dried and blocked for non-specific fluorescence with Fc Receptor Block and Background Buster (Innovex Biosciences, Richmond, CA, USA). Sections were stained with the indicated antibodies and subsequently stained with DAPI (Invitrogen). Digital images were taken using an Olympus BX51 with a Planapo20/0.7 or Planapo40/0.95 objective, a Roper Coolsnap HQ camera and Metamorph software (Molecular Devices, Sunnyvale, CA, USA). Images were acquired using the same exposures for antibody and isotype staining and identical scaling was applied.

### Cell phenotype

Cells were stained with 7-AAD, anti-HLA-DR-AF700, anti-Langerin PE or anti-Langerin AF488 (in-house), anti-CD1c-AF647 (Biolegend) and anti-CD14-eFluor450. Cells were also stained with anti-LOX-1 (clone 15C4, in-house) [21], anti-DC-SIGN (BD Biosciences), anti-DC-ASGPR, [21] anti-DCIR (clone 9E8, in-house), anti-DEC205 (Biolegend) and anti-CD40 (BD Biosciences). Phenotypes of vaginal APCs were analyzed by flow cytometry on an LSR II (BD Biosciences).

### mRNA preparation and hybridization

Total RNA was isolated from cell lysates using the ArrayPure-Nano-scale RNA Purification Kit (Epicentre, Madison, WI, USA) according to the manufacturer’s instructions. RNA (250 ng) from all samples passing quality control was amplified and labeled using the TargetAmp™ 2-Round aRNA Amplification Kit 2.0 (Epicentre). Amplified labeled RNA (750 ng) was hybridized overnight to Illumina HT12 V4 beadchips (Illumina, San Diego, CA, USA). Chips were scanned on an Illumina BeadStation 500 following the manufacturer’s protocols.

### Data pre-processing and batch correction

Raw data were normalized (average) in Genome Studio™ (Illumina). Data were normalized to the median of the 80 samples in Genespring 7.3 (Agilent Technologies, Santa Clara, CA, USA). To identify technical sources of variability, we conducted principal component analysis (PCA) and principal variance component analysis (PVCA) using the 27,935 detected genes (Illumina detection *P*-values <0.01 in at least 1 of 80 samples). To correct the batch effect, we conducted Combat correction using the SVA package from R/Bioconductor [22]. The batch effect’s contribution to variability was removed, as shown by PVCA in Additional file 2.

### Analysis of variance

One-way Welch analysis of variance (ANOVA) was conducted using a *P*-value cutoff of 0.05 and Benjamini-Hochberg multiple testing correction.

### Principal variance component analysis

The weighted average proportion variance was calculated with the R/Bioconductor package 'pvca' (version 1.0.0) [23]. The threshold used for the minimum amount of the variance explained by the selected principal components was 0.5.

## Results

### Eight populations of human antigen-presenting cells

We isolated eight populations of human APCs - four vaginal populations, three skin populations and blood mDCs - and characterized their transcriptional profiles by microarray. The isolation protocol and sort gating strategy are presented in Figure 1. The same protocol was used to isolate cells from the vaginal mucosa and the skin: i) the biopsy samples were cultured overnight in the presence of dispase; ii) the skin epidermis and the dermis, or vaginal epithelium and LP, were separated and further incubated to allow APC migration into the media (Figure 1a); and iii) the cells were sorted using the same antibodies (Figure 1b).

From the vaginal epithelium and LP, vaginal vLCs, vCD14^-^ DCs, vCD14^+^ DCs and vMøs were obtained. HLA-DR^-^ cells were controls. From the skin, epidermal sLCs, sCD14^-^ DCs and sCD14^+^ DCs were obtained. Blood mDC were sorted from buffy coats.

### Global transcriptional relationships across APC populations and tissues

**Figure 2:**
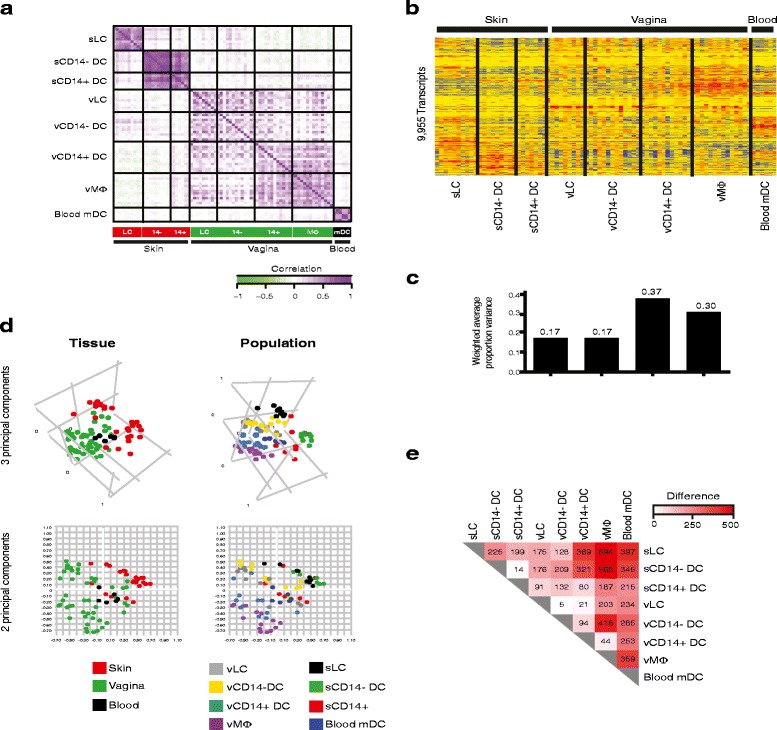
**Unsupervised analysis highlights transcriptional relationships between antigen-presenting cell populations. (a)** Correlation matrix of samples obtained from the 27,935 transcripts detected in the dataset (Illumina detection *P*-values <0.01 in at least 1 of 80 samples). **(b)** Hierarchical clustering (Pearson correlation) of the 9,955 transcripts differentially expressed between the eight populations studied. **(c)** Histogram representing the weighted average proportion variance for tissue, cell population and the interactive term of these two parameters measured by PVCA. **(d)** Principal component analysis of the 80 samples analyzed, classified according to the normalized expression of the 9,955 transcripts identified above. The samples are colored either by tissue (left panel) or cell population (right panel). Both three-dimensional (upper panels) and two-dimensional PCA are displayed (lower panels). **(e)** Heatmap representing the results of Tukey’s test. The values plotted represent the number of transcripts differentially expressed between each pair of cell populations.

To compare the transcriptional profiles of these eight APC populations, we first conducted correlation analysis of all samples starting from the 27,935 transcripts detected in this dataset (Illumina detection *P*-values <0.01 in at least 1 of 80 samples). The matrix in Figure 2a displays correlations between samples within and across cell populations and tissues. sLCs and mDCs showed patterns distinct from all other populations. Dermal sCD14^-^ and sCD14^+^ DCs displayed high correlation, highlighting their transcriptional similarities. Although the four vaginal APC subsets displayed significant variability within each population, vLCs correlated more strongly with vCD14^-^ DCs, while vCD14^+^ DCs correlated more strongly with vMøs. This observation is in agreement with the previously described functional similarities between these two pairs of human vaginal APC subsets [5].

One-way ANOVA identified 9,955 differentially expressed transcripts (DETs) between the eight APC populations. These transcripts were hierarchically clustered and represented as a heatmap in Figure 2b. To identify the known experimental parameters that most influenced sample clustering, we conducted PVCA. Taking into account tissue, cell population, and the interaction between these two parameters, we found that the tissue of origin (skin, vagina or blood) explained the largest proportion of variance (0.37), followed by cell population (0.17) (Figure 2c). This observation was further substantiated using PCA (Figure 2d) and hierarchical clustering (Additional file 3), where samples primarily clustered by tissue. In the vagina, vLCs and vCD14^-^ DCs clustered together, while vCD14^+^ DCs and vMøs formed another cluster, consistent with the correlation levels observed between these APC subsets in Figure 2a.

Finally, Tukey’s test following ANOVA identified the transcripts differentially regulated between populations in a pair-wise fashion. The results are displayed as a heatmap representing the number of DETs between each population pair (Figure 2e). The most distant populations were vMøs and sLCs (694 DETs), while vLCs and vCD14^-^ DCs were the most similar (only 5 DETs).

Thus, unsupervised analysis identified relative transcriptional similarities and differences between the eight APC populations considered, highlighting the importance of the tissue of origin in determining global transcriptional fingerprints.

### Tissue- and population-specific transcriptional profiles

**Figure 3:**
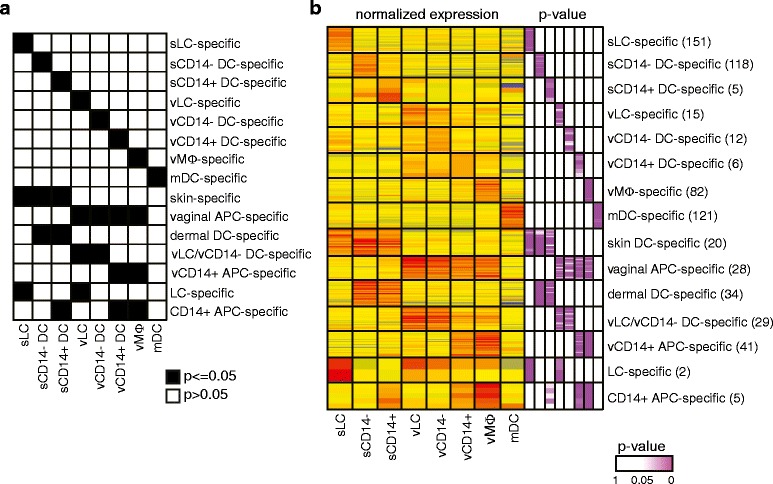
**Analysis of variance identifies population-specific transcripts. (a)** Heatmap describing the filtering method applied to identify population-specific transcripts. **(b)** Heatmap representing the normalized expression of transcripts specifically over-expressed in each APC population studied, as well as groups of populations or tissues. *P*-values are represented in purple gradient on the right.

To further understand the differences in transcriptional profiles between these eight APC populations, we conducted additional variance analysis. The 9,955 DETs resulting from the ANOVA (Figure 2b) were filtered based on the *P*-value for each population. For each subset, we selected transcripts that were differentially expressed from the mean of all samples (*P* < 0.05), requiring that the *P*-value for all populations other than the one of interest remains greater than 0.05 (Figure 3a). This approach also enables the identification of genes specifically modulated in a group of populations compared with others, such as genes specific for skin (*P* < 0.05 in sLCs, sCD14^-^ DCs and sCD14^+^ DCs), vagina (*P* < 0.05 in vLCs, vCD14^-^ DCs, vCD14^+^ DCs and vMøs) or LCs (*P* < 0.05 in sLCs and vLCs), for example. The results of this analysis are displayed in Figure 3b, with the *P*-value for each population displayed on the right panel of the heatmap. To focus on over-expressed transcripts, we additionally filtered significant genes for a minimum 1.5-fold increase compared with the normalized mean across samples.

In this fashion, we identified 151 transcripts specifically over-expressed in sLCs, 118 in sCD14^-^ DCs, 5 in sCD14^+^ DCs, 15 in vLCs, 12 in vCD14^-^ DCs, 6 in vCD14^+^ DCs, 82 in vMøs and 121 in mDCs. In addition, 20 transcripts were specifically over-expressed in sDCs and 28 in vaginal APCs. Population-specific transcripts are represented as knowledge-based Ingenuity Pathway Analysis (IPA) networks in Additional files 4 and 5. These groups of genes can act as potential molecular biomarkers for the APC subsets considered herein.

### Two major vaginal APC transcriptional phenotypes

**Figure 4:**
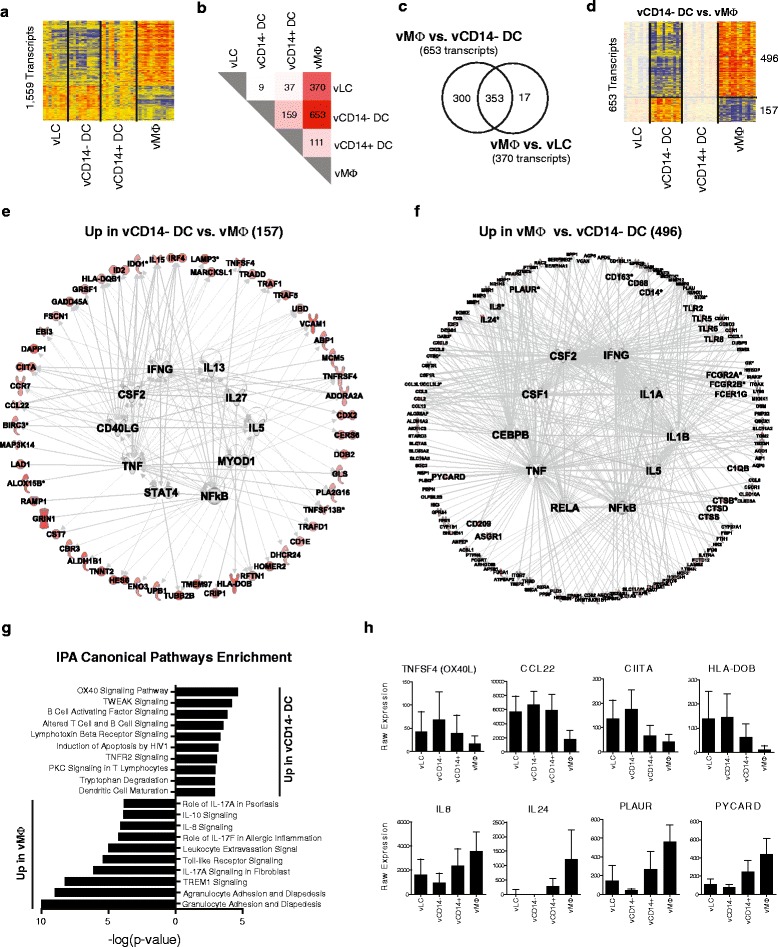
**Transcriptional analysis of vaginal antigen-presenting cell subsets. (a)** Hierarchical clustering (Pearson correlation) of the 1,559 transcripts differentially expressed (one-way ANOVA, *P* < 0.05, Benjamini-Hochberg correction) between the four vaginal APC populations. **(b)** Heatmap representing the results of Tukey’s test conducted after ANOVA. **(c)** Venn diagram of the 653 and 370 transcripts differentially expressed between vCD14^-^ DCs and vMøs and between vLCs and vMøs. **(d)** Hierarchical clustering (Pearson correlation) of the 653 transcripts differentially expressed between vCD14^-^ DCs and vMøs. **(e)** IPA network analysis for the 157 transcripts over-expressed in vCD14^-^ DCs compared with vMøs. **(f)** IPA network analysis for the 496 transcripts over-expressed in vMøs compared with vCD14^-^ DCs. **(g)** Bar chart representing the IPA canonical pathway enrichment in vCD14^-^ DCs and vMøs. **(h)** Bar charts representing the batch-corrected expression values for selected transcripts over-expressed in vCD14^-^ DCs (top row) or vMøs (bottom row).

We then compared the four subsets of vaginal APCs independently of other populations. The 42 samples obtained from vaginal tissue were normalized to the median of all samples. One-way ANOVA identified 1,559 DETs between the four populations. Hierarchical clustering supported the similarity between vLCs and vCD14^-^ DCs as well as between vCD14^+^ DCs and vMøs (Figure 4a). Tukey’s test identified the DETs between vaginal APC populations in a pairwise fashion (Figure 4b). vMøs and vCD14^-^ DCs were most distant, with 653 DETs. Only nine genes separated vLCs and vCD14^-^ DCs. Venn diagram analysis confirmed that the majority of genes (353 out of 370) differentially expressed between vLCs and vMøs were also differentially expressed between vCD14^-^ DCs and vMøs (Figure 4c). Functionally, vLCs and vCD14^-^ DCs displayed similar capacities to induce T-cell responses [5].

The 653 DETs between vCD14^-^ DCs and vMøs are presented as a heatmap (Figure 4d) and further analyzed for regulation and enrichment of biologically relevant pathways by IPA. The predicted regulatory pathways were represented as circular networks with the transcripts on the circumference and the transcription factors and cytokines enriched by *in silico* IPA network growth in the center. The 157 transcripts over-expressed in vCD14^-^ DCs included transcripts related to antigen processing and presentation (LAMP3, HLA-DOB, HLA-DQB1, CIITA, CD1E), DC maturation (CCR7), interaction with lymphocytes (VCAM-1) [24] and Th2 activation molecules (TNFSF4 (OX40L), CCL22) (Figure 4e) [25,26]. These transcripts were connected *in silico* to molecules involved in the activation of T-helper cells, including CD40LG, IL5, IL13 and IFNG. In addition, vCD14^-^ DCs expressed increased levels of the regulatory T cell (Treg) inducer IDO1 [27], the anti-inflammatory gene *RAMP1* [28] and B-cell activation and survival factor TNFSF13B [29]. Conversely, the 496 transcripts over-expressed in vMøs were enriched for Mø markers (CD14, CD163, CD68) and innate pro-inflammatory molecules (IL8, PLAUR, cathepsins, Fc receptors, CXCLs, complement and bacterial Toll-like receptors (TLRs)). These genes were connected *in silico* to major inflammatory mediators, including IL1A/B, NFKB/RELA, TNF and colony stimulating factors CSF1 and CSF2 (Figure 4f). In addition, vMøs expressed increased levels of IL24 transcript, which encodes for a cytokine known to promote Th1 polarization [30].

These observations were further supported by IPA canonical pathway and Gene Ontology biological process enrichment analyses, which identified the Th2-inducing OX40 signaling pathway as the most highly enriched pathway in genes over-expressed in vCD14^-^ DCs. Several innate and inflammatory pathways were enriched in vMøs (Figure 4g; Figure S3a in Additional file 6), including signaling of IL8, TLR and the triggering receptor expressed on myeloid cells type-1 (TREM-1), which is an orphan receptor of the immunoglobulin superfamily induced by the DAP12 signaling pathway. TREM-1 activation induces the production of inflammatory cytokines, including IL8, MCP/CCL2 and TNF [31]. The same observations were made when comparing vLCs and vMøs (Figure S3b in Additional file 6; Additional file 7). The batch-corrected expression of transcripts linked to Th2 induction (TNFSF4, CCL22, CIITA, HLA-DOB) and inflammation (IL8, IL24, PLAUR, PYCARD), which are over-expressed in CD14^-^ vaginal APCs and CD14^+^ vaginal APCs complemented the observations made at the pathway level (Figure 4h).

Altogether, these data suggest that vLCs and vCD14^-^ DCs have a transcriptional phenotype oriented towards the activation of Th2 cells. This provides a molecular basis for our previous finding that these populations can differentiate allo-naive CD4^+^ T cells into Th2 cells *in vitro* [5]. In addition, increased expression of IDO and RAMP1 in vCD14^-^ DCs and vLCs suggests that these cells may also have regulatory functions. In contrast, the transcriptionally similar vCD14^+^ DCs and vMøs are geared for innate immunity, inflammation, pathogen-associated molecular patterns (PAMPs) detection and Th1-type responses.

### A regulatory profile in skin epidermal Langerhans cells

**Figure 5:**
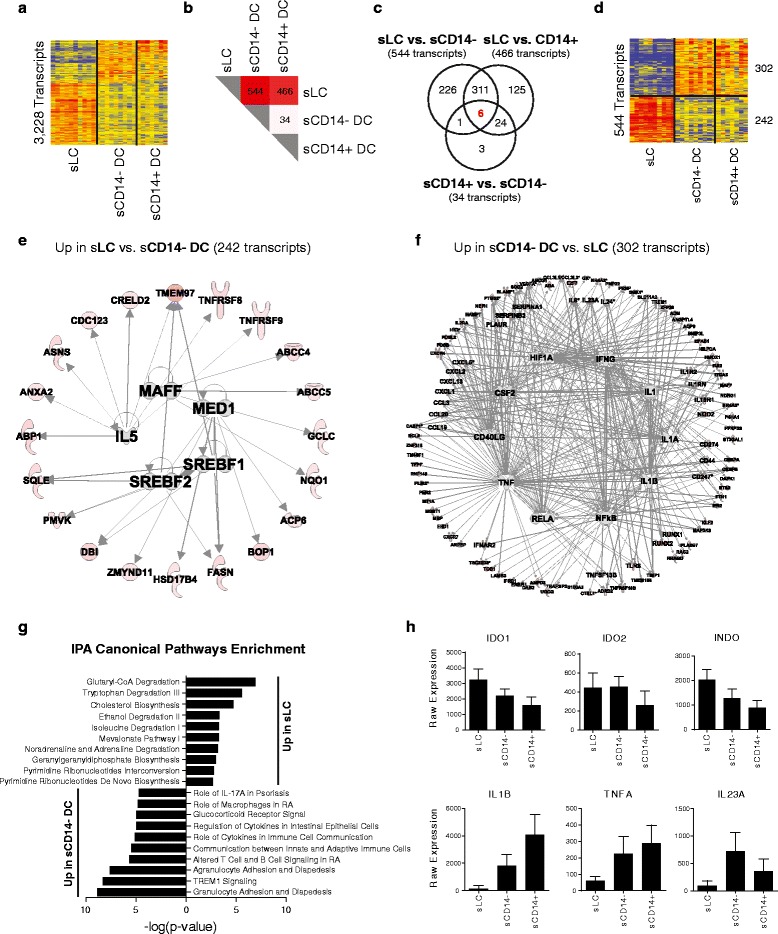
**Transcriptional analysis of skin dendritic cell subsets. (a)** Hierarchical clustering (Pearson correlation) of the 3,228 transcripts differentially expressed (one-way ANOVA, *P* < 0.05, Benjamini-Hochberg correction) between the three skin DC populations. **(b)** Heatmap representing the results of Tukey’s test conducted after ANOVA. **(c)** Venn diagram of the three comparisons conducted during the *post hoc* test. **(d)** Hierarchical clustering (Pearson correlation) of the 544 transcripts differentially expressed between sLCs and sCD14^-^ DCs. **(e)** IPA network analysis for the 242 transcripts over-expressed in sLCs compared with sCD14^-^ DCs. Molecules are colored according to their fold change in the condition represented compared with the median. **(f)** IPA network analysis for the 302 transcripts over-expressed in sCD14^-^ DCs compared with sLCs. **(g)** Bar chart representing the IPA canonical pathway enrichment in sLCs and sCD14^-^ DCs. **(h)** Bar charts representing the batch-corrected expression values for selected transcripts over-expressed in sLCs (top row) or sCD14^-^ DCs (bottom row).

We then conducted a similar analysis in skin DC populations, independently of other APC subsets. We identified 3,228 DETs between sLCs, sCD14^-^ DCs and sCD14^+^ DCs (Figure 5a). Hierarchical clustering of these transcripts highlighted the transcriptional distance between sLCs and sCD14^-^ DCs, the latter population being more similar to sCD14^+^ DCs. This contrasts with vaginal APCs, where vLCs and vCD14^-^ DCs were almost identical transcriptionally (Figure 4a). Thus, the main separation in the skin results from the epidermal and dermal compartmentalization, which was not the case in the vaginal mucosa (Figure 4). This was further confirmed by Tukey’s test, which identified 544 DETs between sLCs and sCD14^-^ DCs and 466 between sLCs and sCD14^+^ DCs (Figure 5b). Of these, 317 transcripts were shared between the two comparisons as shown by Venn diagram (Figure 5c). Only six transcripts representing four genes were differentially expressed between all three comparisons (CXCL1, CXCL5, IL24 and CTSL1; Additional file 8), which could serve as molecular biomarkers of these three skin DC subsets.

The 544 DETs between sLCs and sCD14^-^ DCs were clustered (Figure 5d) and subjected to IPA network and pathway enrichment analysis. The regulatory network of the 242 transcripts over-expressed in sLCs included sterol regulatory element-binding proteins SREBF1 and SREBF2, two molecules involved in the regulation of lipid and cholesterol biosynthesis [32] (Figure 5e). The transcripts enriched in sLCs were enriched for cholesterol biosynthesis, as well as glutaryl-CoA degradation, tryptophan degradation and the melavonate pathway, three pathways linked to T-cell regulation by DCs and involving the regulatory enzyme indolamine 2,3-dioxygenase (INDO/IDO) [27] (Figure 5g). The transcripts encoding this protein (INDO and IDO1) were over-expressed in sLCs, while both dermal CD14^-^ and CD14^+^ DC subsets were enriched in pro-inflammatory molecules IL1B, TNFA and IL23A (Figure 5h).

Conversely, the 302 transcripts over-expressed in sCD14^-^ DCs (and sCD14^+^ DCs) included innate immunity and pro-inflammatory genes (CCLs, CXCLs, SERPINAs, IL8, IL23A), and were connected to major inflammatory cytokines (IL1, IFNG, TNF) and transcription factors (NFKB, RELA) (Figure 5f). As observed for vCD14^+^ DC populations, these transcripts were enriched for pathways linked to innate immunity and inflammation, including TREM1 signaling, Mø function and IL17 signaling (Figure 5g; Additional file 9). This was supported by Gene Ontology enrichment analysis, which identified inflammatory response, defense response and response to stress amongst the biological processes enriched in sCD14^-^ DCs (Additional file 10).

Taken together, the three skin DC subsets displayed two major transcriptional phenotypes that were segregated by the tissue compartment in which they localized. Epidermal sLCs displayed a regulatory phenotype, while dermal sCD14^-^ and sCD14^+^ DCs displayed an innate immunity and pro-inflammatory phenotype similar to that of vaginal CD14^+^ APCs.

### Vaginal dendritic cells versus skin dendritic cells

**Figure 6:**
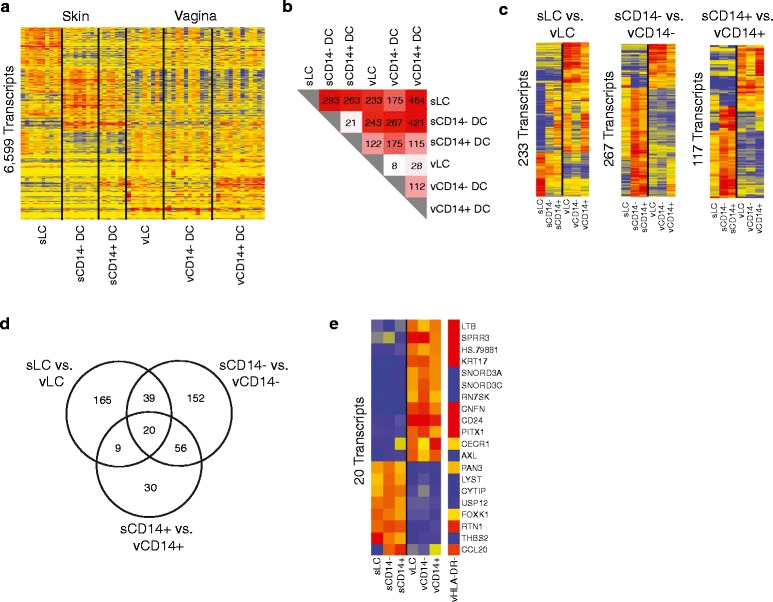
**Comparison between vaginal and skin dendritic cell subsets. (a)** Hierarchical clustering (Pearson correlation) of the 6,559 transcripts differentially expressed (one-way ANOVA, *P* < 0.05, Benjamini-Hochberg correction) between the six skin and vaginal DC populations. **(b)** Heatmap representing the results of Tukey’s test conducted after ANOVA. **(c)** Hierarchical clustering of the transcripts obtained in inter-tissue comparison (skin versus vagina) for each pair of DC subsets. **(d)** Venn diagram of the transcripts obtained in inter-tissue comparison (skin versus vagina) for each pair of DC subsets. **(e)** Hierarchical clustering of the 20 transcripts from the overlap in (d).

To further understand the effect of tissue origin on transcriptional phenotype, we compared DC populations from vagina and skin. To this end, LC, CD14^-^ DC and CD14^+^ DC samples from vagina and skin were recombined into a single experiment and analyzed. vMøs were not included as their skin counterparts were not accessible. One-way ANOVA identified 6,599 DETs between the six populations (Figure 6a). Tukey’s test identified DETs in a pairwise fashion, with the greatest transcriptional difference between sLCs and vCD14^+^ DCs (Figure 6b). The DETs between sLCs and vLCs, sCD14^-^ and vCD14^-^ DCs and sCD14^+^ and vCD14^+^ DCs are clustered and represented as heatmaps in Figure 6c.

Venn diagrams identified overlap between the three comparisons, as well as DETs in each comparison (Figure 6d). These included 12 transcripts over-expressed in vagina and 8 transcripts over-expressed in skin in all three comparisons (Figure 6e), providing a 20 transcript tissue-specific signature. As a non-APC tissue control, transcriptional profiles for vaginal HLA-DR^-^ cells were also obtained (seven biological replicates). Among the genes enriched in vaginal tissue, LTB (lymphotoxin-β) and CD24 were also over-expressed in HLA-DR^-^ vaginal cells. Lymphotoxin signaling is important for the development and maintenance of lymphoid tissue structure, including Peyer’s patches in the mucosa [33], and is also associated with the ability of mucosal DCs to regulate IgA production by plasma cells [34]. CD24 expressed on DCs acts as a co-stimulatory molecule for Th17 cells [35] and cytotoxic T lymphocyte responses [36]. In addition, AXL, a member of the TAM receptor tyrosine kinase family involved in regulation of TLR and interferon-induced inflammatory cascades, was selectively over-expressed in HLA-DR^+^ vaginal populations. sDCs were enriched for CYTIP, a molecule involved in DC motility [37] that also displays regulatory functions in mouse DCs [38]. The DETs in each of the three comparisons are hierarchically clustered and displayed in Additional files 11, 12 and 13.

We also compared vDCs and sDCs by *t*-test, grouping cells by tissue, and including HLA-DR^-^ vaginal cells as control. We found 1,007 DETs between sDCs and vDCs, with 640 over-expressed in sDCs and 367 over-expressed in vDCs (Figure S11a in Additional file 14). The transcripts over-expressed in sDCs were enriched for metabolic processes, including metabolism of nitrogen compounds and RNA, while the transcripts over-expressed in vDCs were enriched for immune-related biological processes, including defense response, phagocytosis and cell adhesion (Figure S11b in Additional file 14). We further compared the 367 transcripts over-expressed in vDCs (compared with sDCs) to HLA-DR^-^ vaginal cells. Of these, 88 were similarly expressed, 123 were under-expressed and 156 were over-expressed in HLA-DR^-^ vaginal cells. The genes over-expressed in vaginal HLA-DR^-^ cells compared with vaginal HLA-DR^+^ cells included many transcripts expressed in epithelial cells, such as keratins, defensins and serpinases, with network connections to inflammatory cytokines such as IL17, IL1 and type I and II interferon (Figure S11c in Additional file 14). The 123 transcripts over-expressed in HLA-DR^+^ DCs were enriched for CLEC10A, OLR1, cathepsins, CD1s and Mø markers such as CD163.

Globally, tissue comparisons yielded more transcripts linked to immune response and inflammation in vDCs, while sDCs exhibited a metabolism fingerprint.

### Pattern-recognition receptor expression in vaginal APC subsets

To further understand the phenotype of vaginal APC subsets and the immunology of the human vagina, we analyzed the expression levels of pattern-recognition receptors, including C-type lectin-like receptors (LLRs) and TLRs, and compared them with those of skin and blood DC subsets. Vaginal APC populations expressed increased levels of CLEC5A, CLEC4F, CLEC4A, CLEC2B, CLEC16A, OLR1, CLEC10A, and CD209 compared with other LLRs (Additional file 15). Consistent with the transcriptional phenotypes of vaginal APCs (Figure 4), vLCs were close to vCD14^-^ DCs while vCD14^+^ DCs were close to vMøs in terms of expression levels of the eight LLRs. Both CD209 and OLR1 were highly expressed especially in vCD14^+^ DCs and vMøs. vCD14^+^ DCs and vMøs expressed higher levels of CD209 and OLR1 than sCD14^+^ DCs. In addition, CLEC5A was expressed in vLCs but not sLCs. Similarly, vCD14^-^ DCs expressed CLEC4F, while sCD14^-^ DCs showed no or minimal expression of CLEC4F. Compared with vaginal APCs and sDCs, blood mDCs expressed increased levels of CLEC2B, CLEC10A, and CLEC12A, but decreased levels of CD209, CLEC5A, and CLEC4F. Furthermore, batch-corrected expression analysis identified three additional lectins that were over-expressed in vCD14^+^ DCs and vMøs: CLEC2B, CLEC5A and LGALS8 (Additional files 15 and 16). Conversely, only CLEC16A was over-expressed in vLCs and vCD14^-^ DCs.

In contrast to LLRs, vDC subsets and their skin counterparts expressed similar levels of TLRs, MDA5, and RIG-I (Additional file 17). However, CD14^+^ populations, particularly vMøs, expressed increased levels of bacterial-sensing TLRs (TLR2, TLR5, TLR6, TLR8).

**Figure 7:**
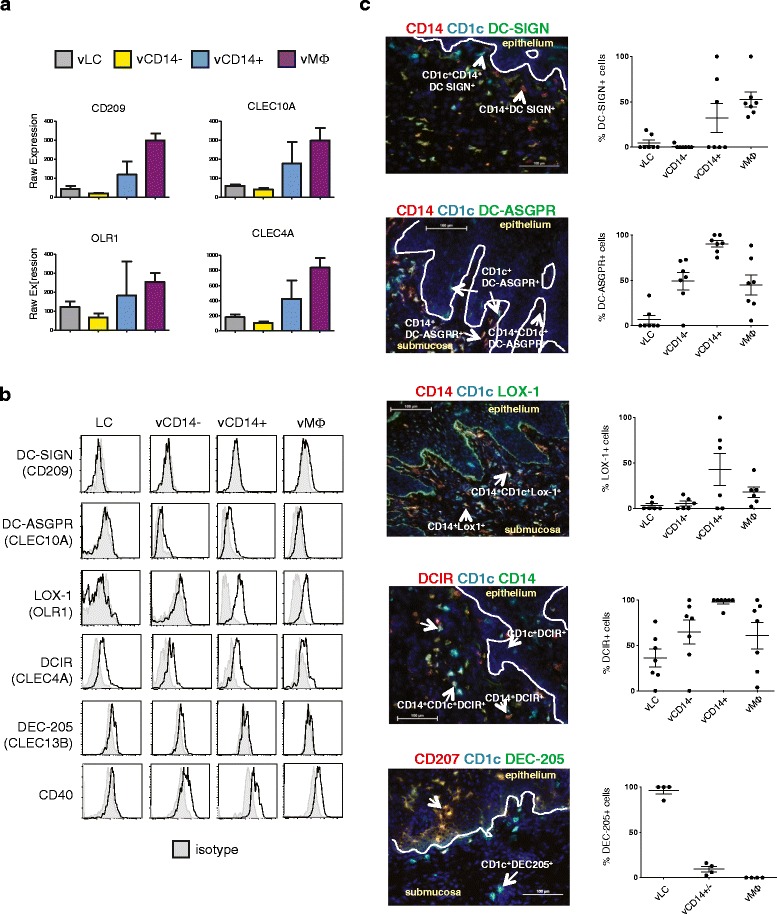
**Surface receptor expression on vaginal antigen-presenting cells. (a)** Transcriptional levels of CD209 (DC-SIGN), OLR1 (LOX-1), CLEC10A (DC-ASGPR) and CLEC4A (DCIR) in the four vaginal APC populations. Bar charts represent the mean ± standard deviation of batch-corrected expression data. **(b)** Fluorescence-activated cell sorting (FACS) analysis of CD209, DC-ASGPR, LOX-1, DCIR, DEC-205 and CD40 expression on the surface of vaginal APC subsets. Vaginal cell suspensions were stained with the indicated antibodies and gated as described in Figure 1b. Gray histograms represent isotype controls. Data are representative of 10 donors. **(c)** Frozen tissue sections were stained for (i) DC-SIGN, DC-ASGPR or LOX-1 (green), CD1c (light blue); and CD14 (red); or (ii) DCIR (red), CD1c (light blue) and CD14 (green); or (iii) DEC-205 (green), CD1c (light blue) and CD207 (red) (DAPI (dark blue), ×20, the horizontal bar represents 100 μm). Data are representative of four to eight independent experiments (left panel) or combined (right panel). Isotype controls are presented in Additional file 18.

We next assessed the expression levels of LLRs and other receptors that have been previously tested for vaccines targeting *in vivo* DCs [21,39-43]. CD209 and CLEC10A, OLR1, and CLEC4A, but not CLEC13B, were detected in all four vaginal APC subsets with increased expression of CD209, CLEC10A, and CLEC4A in vMøs (Figure 7a; Additional file 18). The expression of DC-SIGN, DC-ASGPR, and LOX-1 was confirmed on vCD14^+^ DCs and vMøs at the protein level by flow cytometry (Figure 7b) and *in situ* by immunofluorescence (Figure 7c). LOX-1 was also expressed at low levels on vCD14^-^ DCs (Figure 7b,c). Transcriptional levels of CD207 were low in all vaginal APC subsets, despite detectable protein levels on the surface of vLCs (Figure 1b), as previously reported [5,6]. DC-ASGPR was detected in a few vLCs and approximately 50% (49.06 ± 25) of vCD14^-^ DCs (Figure 7b, c). DCIR was detected on all APC subsets by immunofluorescence and flow cytometry (Figure 7c). DEC-205 was detected on LCs and some submucosal DCs by immunofluorescence and flow cytometry, despite undetectable transcriptional expression. CD40 transcriptional levels were low in all vaginal APC subsets (not shown), although CD40 was detected on all migrated vaginal APC subsets by flow cytometry (Figure 7b).

### Expression of inflammatory mediators in vaginal APC subsets

Finally, we analyzed the expression of cytokines, chemokines and their receptors. The results are summarized in Additional file 19. Dermal DCs and vaginal CD14^+^ populations displayed increased levels of neutrophil chemoattractants CXCL1, CXCL2 and CXCL5 (Additional file 20), as well as pro-inflammatory cytokines IL1A, IL1B, IL24, TNF and IL8 (Additional file 21). All sDC populations expressed higher levels of IL23A, while vaginal populations displayed increased IL32, and mDCs displayed increased IL12A and CXCL10, suggesting tissue-specific capacities to polarize T-helper responses. Dermal DCs expressed higher levels of IL1R1, IL1R2 and IL18R, which may polarize them to respond to products of the inflammasome, while vMøs were enriched in IL10RB and IL17RA, two receptors involved in defense against extracellular pathogens. Interestingly, sLCs expressed increased levels of several CKLF-like MARVEL transmembrane domain-containing proteins (CMTM4, CMTM6, CMTM8), which constitute a novel family of chemokine receptors about which little is known.

## Discussion

We report the first transcriptional characterization of four human vaginal APC subsets by microarray. We compared these transcriptional profiles with those of three skin DC populations and blood mDCs. Combining unsupervised, pathway-level and transcript-level analyses, we identified tissue- and population-specific transcripts, as well as whole molecular pathways that potentially control the biological functions of APCs in the human vaginal mucosa and the skin [3,5,44]. This database of 87 microarray samples obtained from human vagina, skin and blood provides an important resource to understand tissue-specific immunity and guide the rational development of microbial vaccines.

Unsupervised analysis enabled the measurement of the transcriptional separation between APC populations in an unbiased fashion. vLCs were similar to vCD14^-^ DCs, while vCD14^+^ DCs were similar to vMøs, suggesting that CD14 expression can be used to differentiate between two main APC groups with distinct fingerprints. The global transcriptional distance between populations is probably best expressed by PCA (Figure 2d), and can be confirmed by Tukey’s *post hoc* test (Figure 2e). Tukey’s *post hoc* test is a stringent test identifying pairwise differences within the populations considered for ANOVA. Because of the stringency of the test, the number of genes differentially expressed between two populations is affected by the number of samples and variability within groups. vCD14^+^ DCs and vLCs displayed a higher degree of variability across donors than vMøs, thereby explaining lower numbers of genes detected, despite more similar profiles between vCD14^+^ DCs and Møs. In fact, both CD14^+^ populations presented an innate inflammatory profile with increased expression of pattern-recognition receptors, consistent with less mature populations. The CD14^-^ populations were enriched for T-cell co-stimulation and antigen-presentation transcripts, consistent with a more mature profile. The transcriptional proximities between CD14^-^ APCs and CD14^+^ APCs are concordant with the functional capacities we previously described, where vLCs and vCD14^-^ DCs polarize CD4^+^ T cells towards the Th2 phenotype [5]. In this context, the increase in OX40L (TNFSF4), CCL22 and MHC class II-related transcripts (CIITA and HLA-DRs) in CD14^-^ APC populations suggests an OX40L-dependent mechanism of Th2 induction. Interestingly, sDCs did not show the same transcriptional separation based on expression of CD14. Their transcriptional phenotype separated mainly based on physical location, where the two dermal populations displayed a profile distinct from that of sLCs. Finally, it is important to note that the individual APC populations we describe in this manuscript may contain additional levels of heterogeneity. For example, we have previously shown that both CD14^-^ and CD14^+^ LP-DCs could be further separated into two subpopulations based on CD1a expression [5,6]. Further studies, such as single-cell RNA sequencing of populations of interest, are warranted to further characterize the heterogeneity of these populations.

When comparing transcriptional fingerprints between tissues, we observed that sDCs were enriched for metabolic pathways, while vDCs were enriched for immune-related networks. Although the female genital tract is considered an immune-privileged site [10-12], this observation, along with the data from our previous study, [5] supports that DCs in the human vaginal mucosa can elicit immune responses, as previously observed in mice [45]. These findings have important implications for the rational design of mucosal vaccines against sexually transmitted pathogens.

In addition, the transcriptional profiling of skin and vaginal APCs enabled us to formulate new hypotheses on the mechanisms controlling some of the known functions of these subsets. For instance, transcripts encoding IDO1/INDO, a molecule involved in DC-dependent induction of Tregs, were significantly overexpressed in sLCs compared with other sDCs (Figure 5h). These data support recent studies showing the induction of Tregs by sLCs in the absence of foreign antigen stimulation [44,46]. Therefore, these factors should be considered in the development of vaccines that elicit potent immunity in the vaginal mucosa.

The differences observed between tissues may also be influenced by the composition of the microbiome from each tissue. The vaginal microbiome is highly enriched in lactobacilli [47], while the skin microbiome is enriched for staphylococci and actinobacteria [15,48]. In fact, it was shown that microbiota can influence immunity by triggering expression of C-type lectins [49] or controlling the Th1/Th2 balance [50]. The interactions between the microbiome and DC functions in different tissues and the outcomes of immune responses at steady state and during infections need to be further studied.

Finally, we used these data to formulate testable hypotheses regarding the expression of various receptors on the surface of each vaginal APC population. As vaginal CD14^+^ cells displayed a more immature and innate phenotype, it was logical to find that transcripts for receptors that could be used as targeting molecules for antibody-conjugated vaccines (such as C-type lectins) were globally enriched in these populations, particularly in vMøs. Of the known lectins, only CLEC16A was transcriptionally enriched in vLCs and vCD14^-^ DCs. Interestingly, this putative immuno-receptor, which belongs to a gene complex involving CIITA and SOCS1, is linked to autoimmune disorders such as multiple sclerosis and rheumatoid arthritis [51,52].

Our study has several limitations. First, the majority of skin samples were obtained from individuals with high body mass index undergoing cosmetic surgeries. Therefore, we cannot exclude that the lipid-rich environment of the skin affects the transcriptional profiles observed. Although inflamed vaginal tissues were excluded in this study, a large fraction of samples were from patients who underwent pelvic surgeries. These challenges are common in the study of human immunology using surgical tissue samples. Another major limitation is the *in vivo* relevance of the data generated *in vitro*. The data generated with human tissue samples still needs to be validated *in vivo*. In this respect, the surface biomarkers of the vaginal APC subsets characterized in this study are important findings and clinically relevant because we can now design mucosal vaccines that can target specific receptors that are expressed on specific subsets of the vaginal APCs. Vaccines targeting the proper subsets of DCs in conjunction with appropriate adjuvants have proven an efficient strategy to elicit potent immunity [21,40-43].

To conclude, this study provides new insights on the molecular mechanisms that regulate the functions of vaginal APC subsets. The identification of population-specific biomarkers combined with an understanding of major functional characteristics of each APC population in the vaginal mucosa will be important for the development of targeted vaccines against sexually transmitted pathogens, as well as cancers, in the female genital tract.

## Conclusions

We generated a transcriptional dataset of 87 microarray samples spanning eight APC populations across human vagina, skin and blood. Complementary transcript and network-level analyses of these data, combined with *in situ* immunohistochemistry of major pattern recognition receptors in the vaginal mucosa, permitted the phenotypic and functional characterization of these populations. By comparing vagina, skin and blood, we found that APC genomic fingerprints are significantly influenced by the tissue of origin, revealing tissue-specific microenvironments. Nonetheless, CD14^+^ APCs from both vagina and skin are geared towards innate immunity and pro-inflammatory responses, whereas CD14^-^ DCs, particularly sLCs, vLCs, and vCD14^-^ DCs, display both Th2-inducing and regulatory phenotypes. These data will help the further characterization of human tissue APC lineages and will guide the design of mucosal vaccines against sexually transmitted pathogens.

### Data access

The dataset described in this manuscript is deposited in the NCBI Gene Expression Omnibus [53] (GEO series accession number GSE54480). Both background-subtracted and batch-corrected expression datasets are presented.

## Additional files

Additional file 1: Figure S16. The expression of CD1a on the skin DCs. Additional file 2: Figure S17. Results of batch correction. Additional file 3: Figure S1. Heatmap showing DETs between the eight populations. Additional file 4: Figure S2. Population-specific transcript networks. Additional file 5: Table S1. Lists the expression of population-specific transcripts. Additional file 6: Figure S3. Pathway enrichment scores in vaginal DC subsets. Additional file 7: Figure S4. Results of the comparisons between vaginal LC and macrophages. Additional file 8: Figure S5. Transcripts specific for skin APC populations. Additional file 9: Figure S6. Results of comparisons between sLCs and sCD14^+^ DCs. Additional file 10: Figure S7. Pathways enrichment scores in skin LCs and skin CD14^-^ DCs. Additional file 11: Figure S8. DETs between skin and vaginal APC subsets. Additional file 12: Figure S9. DETs between skin and vaginal APC subsets. Additional file 13: Figure S10. DETs between skin and vaginal APC subsets. Additional file 14: Figure S11. IPA analysis of DETs in skin and vagina. Additional file 15: Figure S12. Raw transcriptional expression of C-type lectins, TLRs, chemokines, cytokines and their receptors in all APC subsets. Additional file 16: Table S2. Expression of surface molecules, cytokines and chemokines on all APC subsets. Additional file 17: Figure S13. Raw transcriptional expression of C-type lectins, TLRs, chemokines, cytokines and their receptors in all APC subsets. Additional file 18: Figure S18. Immunofluorescence staining of lectin receptors on human vagina. Additional file 19: Table S3. Expression of surface molecules, cytokines and chemokines on all APC subsets. Additional file 20: Figure S14. Raw transcriptional expression of C-type lectins, TLRs, chemokines, cytokines and their receptors in all APC subsets. Additional file 21: Figure S15. Raw transcriptional expression of C-type lectins, TLRs, chemokines, cytokines and their receptors in all APC subsets.

## Abbreviations

ANOVA: analysis of variance
APC: antigen-presenting cell
DC: dendritic cell
DET: differentially expressed transcript
IL: interleukin
IPA: Ingenuity Pathway Analysis
IRB: institutional review board
LC: Langerhans cell
LLR: lectin-like receptor
LP: lamina propria
mDC: myeloid dendritic cell
Mø: macrophage
PCA: principal component analysis
PVCA: principal variance component analysis
sLC: skin Langerhans cell
TLR: Toll-like receptor
TNF: tumor necrosis factor
Treg: regulatory T cell
