# Supplementary material for: Transcriptional fingerprints of antigen-presenting cell subsets in the human vaginal mucosa and skin reflect tissue-specific immune microenvironments
Source: Genome Med. 2014 Nov 25;6(11):98. doi: 10.1186/s13073-014-0098-y (PMC4268898; doi:10.1186/s13073-014-0098-y)
Supplement: Additional file 1: Figure S16. — The expression of CD1a on the skin DCs. [file 13073_2014_98_MOESM1_ESM.pdf]

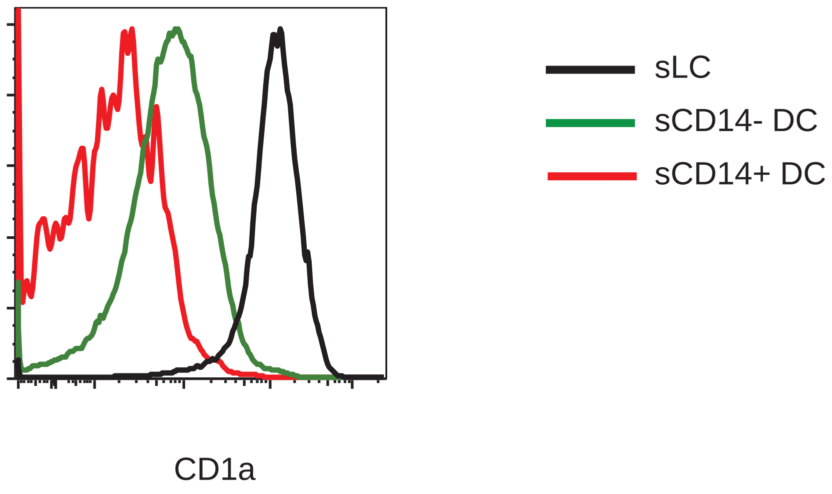

Figure S16: CD1a expression on skin DCs.
CD1a expression was analyzed by flow cytometry on skin LCs (black line), sCD14- DCs (green line) and sCD14+ DCs (red line).
