# Supplementary material for: Transcriptional fingerprints of antigen-presenting cell subsets in the human vaginal mucosa and skin reflect tissue-specific immune microenvironments
Source: Genome Med. 2014 Nov 25;6(11):98. doi: 10.1186/s13073-014-0098-y (PMC4268898; doi:10.1186/s13073-014-0098-y)
Supplement: Additional file 2: Figure S17. — Results of batch correction. [file 13073_2014_98_MOESM2_ESM.pdf]

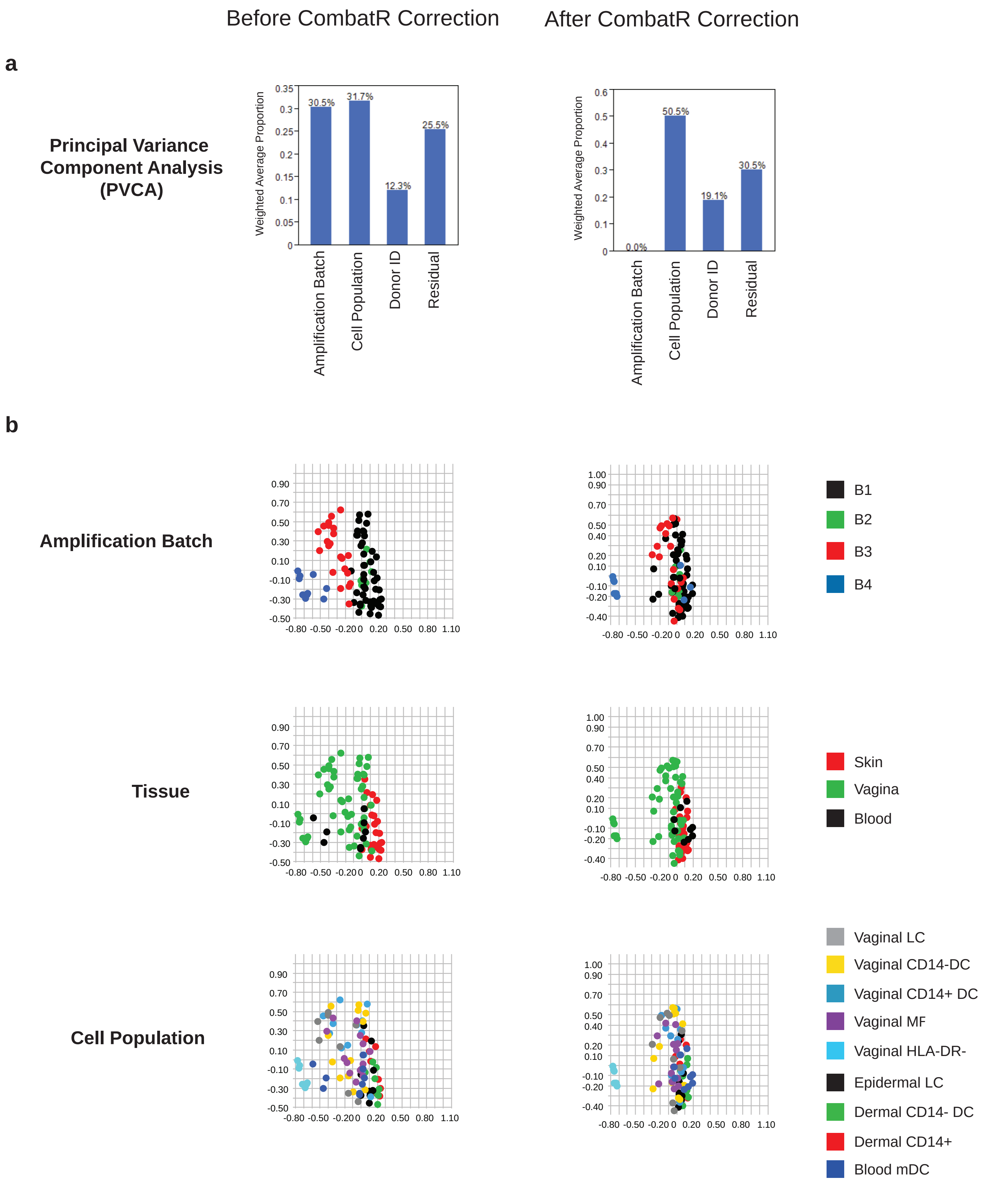

Figure S17 - Batch correction analysis. a. Principal variance component analysis (PVCA) before and after batch correction. **b.** Principal component analysis (PCA) before and after batch correction. Samples are colored by amplification batch, tissue or cell population.
