# Supplementary material for: Transcriptional fingerprints of antigen-presenting cell subsets in the human vaginal mucosa and skin reflect tissue-specific immune microenvironments
Source: Genome Med. 2014 Nov 25;6(11):98. doi: 10.1186/s13073-014-0098-y (PMC4268898; doi:10.1186/s13073-014-0098-y)
Supplement: Additional file 3: Figure S1. — Heatmap showing DETs between the eight populations. [file 13073_2014_98_MOESM3_ESM.pdf]

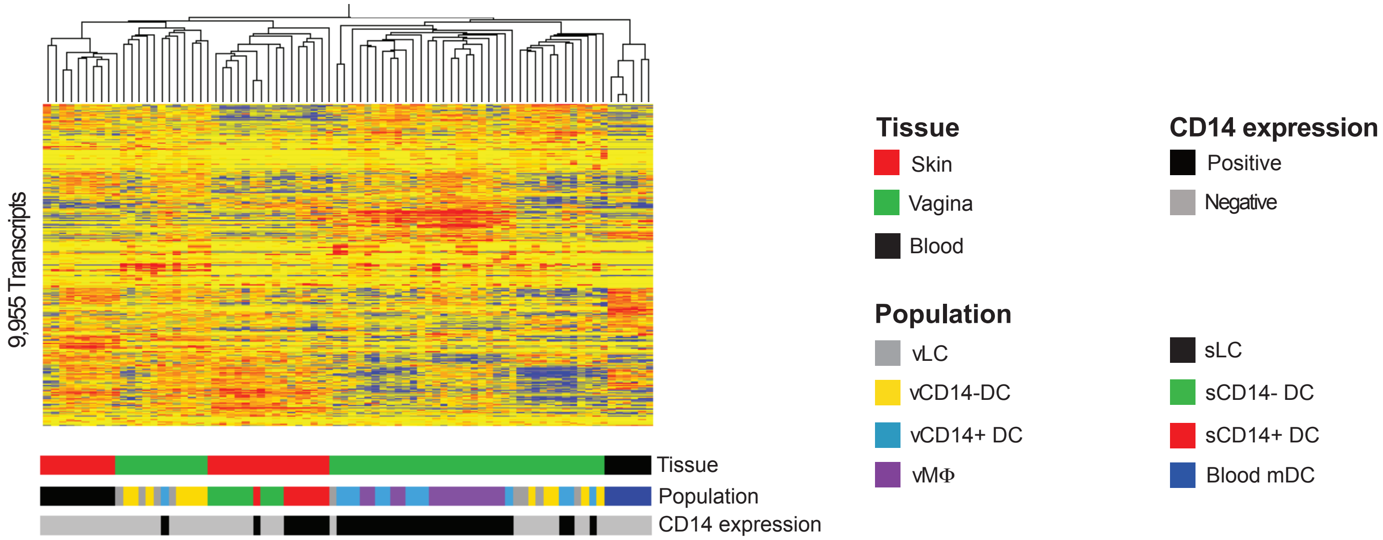

Figure S1: Hierarchical clustering (Pearson correlation) of the 9,955 transcripts differentially expressed between the 8 APC populations. Data are normalized to the median of all samples. Samples are colored by tissue, cell population or CD14 expression
