# Supplementary material for: Transcriptional fingerprints of antigen-presenting cell subsets in the human vaginal mucosa and skin reflect tissue-specific immune microenvironments
Source: Genome Med. 2014 Nov 25;6(11):98. doi: 10.1186/s13073-014-0098-y (PMC4268898; doi:10.1186/s13073-014-0098-y)
Supplement: Additional file 4: Figure S2. — Population-specific transcript networks. [file 13073_2014_98_MOESM4_ESM.pdf]

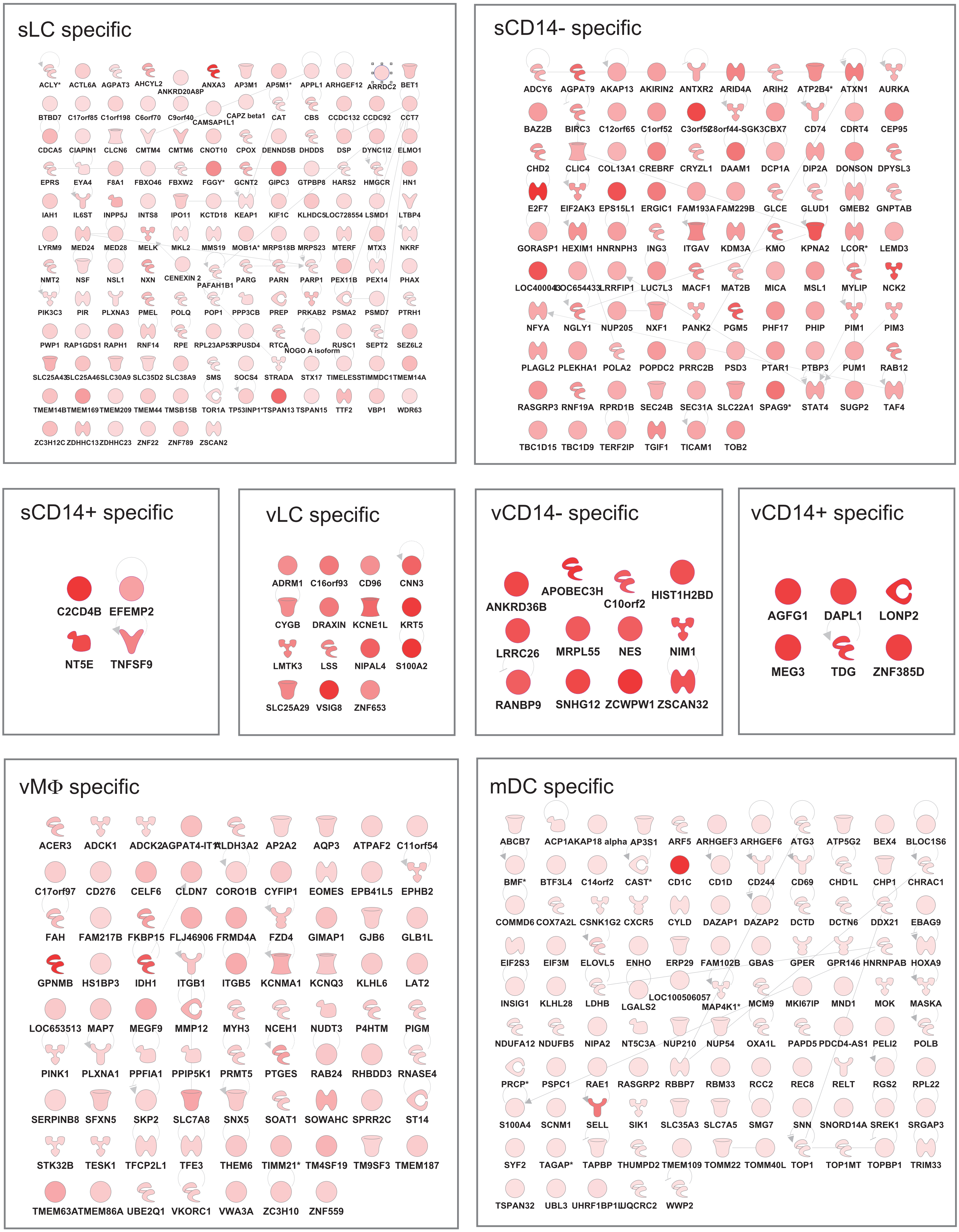

Figure S2: Network representation of population-specific genes identified by variance analysis in Figure 3. Network were built with Ingenuity Pathway Analysis (IPA) software. Genes were connected based on IPA's knowledge-based gene interaction database. The red gradient represents the intensity of over-expression of each gene in the population considered as compared to the median of all samples.
