# Supplementary material for: Transcriptional fingerprints of antigen-presenting cell subsets in the human vaginal mucosa and skin reflect tissue-specific immune microenvironments
Source: Genome Med. 2014 Nov 25;6(11):98. doi: 10.1186/s13073-014-0098-y (PMC4268898; doi:10.1186/s13073-014-0098-y)
Supplement: Additional file 5: Table S1. — Lists the expression of population-specific transcripts. [file 13073_2014_98_MOESM5_ESM.pdf]

Specificity	Illumina ID	Symbol	Definition	Skin			Vagina				Blood
				sLC	sCD14- DC	sCD14+ DC	vLC	vCD14- DC	vCD14+ DC	vMF	mDC
sLC	ILMN_1748546	2-Sep	Homo sapiens septin 2 (SEPT2), transcript variant 1	1.70	0.92	0.83	0.95	0.75	0.79	1.13	1.63
sLC	ILMN_2371379	ACLY	Homo sapiens ATP citrate lyase (ACLY), transcript variant 2	2.21	0.87	0.72	0.93	0.72	0.93	0.95	0.98
sLC	ILMN_1749014	ACLY	Homo sapiens ATP citrate lyase (ACLY), transcript variant 1	2.20	0.83	0.67	0.90	0.78	0.69	0.85	1.27
sLC	ILMN_2328280	ACTL6A	Homo sapiens actin-like 6A (ACTL6A), transcript variant 1	2.16	1.31	1.11	0.90	1.26	0.90	1.33	0.96
sLC	ILMN_1654010	AGPAT3	Homo sapiens 1-acylglycerol-3-phosphate O-acyltransferase 3 (AGPAT	1.56	0.98	1.00	1.16	1.14	1.09	1.06	1.07
sLC	ILMN_1712786	AHCYL2	Homo sapiens S-adenosylhomocysteine hydrolase-like 2 (AHCYL2)	3.75	1.26	1.26	1.12	1.30	1.01	1.12	1.33
sLC	ILMN_2181867	ANKRD20B	Homo sapiens ankyrin repeat domain 20B (ANKRD20B) on chromoso	1.57	0.98	1.09	0.96	0.93	0.97	1.20	0.84
sLC	ILMN_1694548	ANXA3	Homo sapiens annexin A3 (ANXA3)	9.31	0.64	1.03	1.70	1.67	1.03	1.06	0.49
sLC	ILMN_1667086	AP3M1	Homo sapiens adaptor-related protein complex 3, mu 1 subunit (AP3M	1.90	0.82	0.62	0.92	1.11	0.78	1.34	0.88
sLC	ILMN_1763730	APPL1	Homo sapiens adaptor protein, phosphotyrosine interaction, PH domair	1.71	1.43	1.15	1.02	1.02	1.06	0.94	1.42
sLC	ILMN_1810712	ARHGEF12	Homo sapiens Rho guanine nucleotide exchange factor (GEF) 12 (ARI	1.75	1.19	1.14	1.07	1.37	0.89	1.10	0.59
sLC	ILMN_1715181	ARRDC2	Homo sapiens arrestin domain containing 2 (ARRDC2), transcript vari	1.93	1.26	0.93	0.86	0.90	0.89	0.95	0.72
sLC	ILMN_1684042	BET1	Homo sapiens blocked early in transport 1 homolog (S. cerevisiae) (BE	2.53	0.93	1.10	1.00	1.02	1.00	1.00	0.82
sLC	ILMN_1687743	BTBD7	Homo sapiens BTB (POZ) domain containing 7 (BTBD7), transcript va	1.62	1.21	1.16	0.74	0.57	0.60	0.74	0.47
sLC	ILMN_2221076	C17ORF85	Homo sapiens chromosome 17 open reading frame 85 (C17orf85)	1.59	0.78	0.68	1.01	0.98	0.79	0.80	0.98
sLC	ILMN_3253728	C19ORF64	PREDICTED: Homo sapiens chromosome 19 open reading frame 64 (C	5.58	1.14	1.35	1.21	1.26	1.25	0.94	0.90
sLC	ILMN_1657683	C1ORF198	Homo sapiens chromosome 1 open reading frame 198 (C1orf198)	1.98	1.15	1.06	1.23	1.21	1.14	1.09	0.98
sLC	ILMN_2193175	C3ORF1	Homo sapiens chromosome 3 open reading frame 1 (C3orf1)	1.93	1.11	1.26	1.13	1.10	1.12	1.12	0.68
sLC	ILMN_1657064	C6ORF70	Homo sapiens chromosome 6 open reading frame 70 (C6orf70)	1.54	0.95	0.90	1.00	0.89	0.71	0.84	0.73
sLC	ILMN_1711799	C9ORF40	Homo sapiens chromosome 9 open reading frame 40 (C9orf40)	1.56	1.34	1.09	0.97	0.94	0.98	0.97	0.75
sLC	ILMN_1792660	CAMSAP1L1	Homo sapiens calmodulin regulated spectrin-associated protein 1-like 1	1.59	1.33	1.04	0.95	0.78	0.96	1.31	0.41
sLC	ILMN_2053921	CAPZB	Homo sapiens capping protein (actin filament) muscle Z-line, beta (CA	1.63	0.80	0.89	1.14	1.00	1.03	1.11	0.98
sLC	ILMN_2151739	CAT	Homo sapiens catalase (CAT)	1.74	1.40	1.21	0.62	1.18	0.67	0.89	1.11
sLC	ILMN_1804735	CBS	Homo sapiens cystathionine-beta-synthase (CBS)	2.41	0.99	0.97	1.10	1.01	1.11	1.00	0.97
sLC	ILMN_1716342	CCDC132	Homo sapiens coiled-coil domain containing 132 (CCDC132), transcri	2.12	1.00	0.97	0.99	1.12	1.00	1.27	1.18
sLC	ILMN_1731107	CCDC92	Homo sapiens coiled-coil domain containing 92 (CCDC92)	2.28	0.88	0.79	0.79	0.75	0.90	1.24	0.53
sLC	ILMN_1703718	CCT7	Homo sapiens chaperonin containing TCP1, subunit 7 (eta) (CCT7), tra	2.19	0.80	1.03	1.17	0.93	0.84	1.23	0.77
sLC	ILMN_1683450	CDCA5	Homo sapiens cell division cycle associated 5 (CDCA5)	3.12	0.94	1.00	1.48	1.27	1.19	0.79	0.73
sLC	ILMN_1735199	CIAPIN1	Homo sapiens cytokine induced apoptosis inhibitor 1 (CIAPIN1)	1.56	1.01	1.03	1.00	1.17	1.03	1.26	1.37
sLC	ILMN_1741594	CLCN6	Homo sapiens chloride channel 6 (CLCN6), transcript variant ClC-6d	1.74	1.12	0.61	1.01	0.84	0.81	0.84	1.09
sLC	ILMN_1762718	CMTM4	Homo sapiens CKLF-like MARVEL transmembrane domain containin	1.62	1.24	1.26	1.07	1.12	1.12	1.10	0.89
sLC	ILMN_1696494	CMTM6	Homo sapiens CKLF-like MARVEL transmembrane domain containin	2.47	1.15	0.96	0.73	0.69	0.53	0.66	1.76
sLC	ILMN_2078334	CNOT10	Homo sapiens CCR4-NOT transcription complex, subunit 10 (CNOT1	2.00	1.02	0.96	0.86	1.17	0.96	0.78	0.80
sLC	ILMN_3240389	CPOX	Homo sapiens coproporphyrinogen oxidase (CPOX)	2.10	1.44	1.06	1.24	1.35	0.90	0.98	0.82
sLC	ILMN_1791593	DENND5B	Homo sapiens DENN/MADD domain containing 5B (DENND5B)	1.92	1.08	1.13	1.24	1.22	1.09	1.13	0.98
sLC	ILMN_2405642	DHDDS	Homo sapiens dehydrodolichyl diphosphate synthase (DHDDS), transc	1.98	0.92	0.82	1.11	1.11	0.97	1.04	0.84
sLC	ILMN_1655614	DSP	Homo sapiens desmoplakin (DSP), transcript variant 2	2.33	1.06	1.12	2.13	2.25	1.32	1.94	1.00
sLC	ILMN_1773847	DYNC1I2	Homo sapiens dynein, cytoplasmic 1, intermediate chain 2 (DYNC1I2)	2.46	0.93	0.77	0.95	0.80	0.86	1.05	0.49
sLC	ILMN_1784320	ELMO1	Homo sapiens engulfment and cell motility 1 (ELMO1), transcript vari	2.34	1.39	1.39	1.07	1.00	0.84	0.76	1.62
sLC	ILMN_1783695	EPRS	Homo sapiens glutamyl-prolyl-tRNA synthetase (EPRS)	2.24	0.64	0.73	0.97	0.98	0.89	1.19	1.22
sLC	ILMN_1671844	EYA4	Homo sapiens eyes absent homolog 4 (Drosophila) (EYA4), transcript	1.52	1.11	1.00	1.01	1.01	1.01	1.04	0.97
sLC	ILMN_1790249	F8A1	Homo sapiens coagulation factor VIII-associated (intronic transcript) 1	2.20	0.70	0.82	0.75	0.83	1.06	0.97	0.92
sLC	ILMN_1747119	FBXO46	PREDICTED: Homo sapiens F-box protein 46, transcript variant 5 (FB	1.63	0.97	0.87	1.13	1.14	0.84	0.87	0.88
sLC	ILMN_1775753	FBXW2	Homo sapiens F-box and WD-40 domain protein 2 (FBXW2)	1.58	0.86	0.73	0.97	0.90	0.93	1.08	1.09
sLC	ILMN_3271630	FGGY	Homo sapiens FGGY carbohydrate kinase domain containing (FGGY),	4.45	1.08	0.89	1.14	1.22	1.07	1.18	1.04
sLC	ILMN_1737604	FLJ10986	Homo sapiens hypothetical protein FLJ10986 (FLJ10986)	5.12	0.75	0.89	0.87	0.95	0.88	1.12	0.66
sLC	ILMN_1680390	GCNT2	Homo sapiens glucosaminyl (N-acetyl) transferase 2, I-branching enzy	3.20	0.82	0.82	1.17	0.98	0.91	0.96	1.20
sLC	ILMN_1660698	GTPBP8	Homo sapiens GTP-binding protein 8 (putative) (GTPBP8), transcript	1.63	1.18	1.23	0.92	1.05	0.89	0.93	0.96
sLC	ILMN_1715113	HARS2	Homo sapiens histidyl-tRNA synthetase 2, mitochondrial (putative) (H	1.63	1.18	1.21	0.87	0.74	0.75	0.93	0.90
sLC	ILMN_1657395	HMGCR	Homo sapiens 3-hydroxy-3-methylglutaryl-Coenzyme A reductase (HM	2.04	0.99	0.92	1.05	1.18	0.88	0.82	0.79
sLC	ILMN_2384591	HN1	Homo sapiens hematological and neurological expressed 1 (HN1), tran	2.68	1.09	1.13	1.05	1.47	1.02	0.98	0.96
sLC	ILMN_1827211	HS.550193	Human unidentified mRNA, partial sequence	1.77	0.98	1.22	1.00	1.09	1.01	1.07	1.16
sLC	ILMN_1894388	HS.552082	UI-H-FH1-bfi-p-18-0-UI.s1 NCI_CGAP_FH1 Homo sapiens cDNA cl	1.55	0.99	0.92	1.12	1.42	1.09	1.19	0.99
sLC	ILMN_1849013	HS.570988	Homo sapiens primary neuroblastoma cDNA, clone:Nbla10111, full in	2.44	1.31	0.78	0.68	0.82	0.83	0.93	0.56
sLC	ILMN_1833858	HS.66187	Homo sapiens clone 23700 mRNA sequence	3.11	0.96	1.01	1.57	1.37	1.16	1.08	0.88
sLC	ILMN_2217329	IAH1	Homo sapiens isoamyl acetate-hydrolyzing esterase 1 homolog (S. cere	2.30	1.03	0.69	0.94	1.00	0.82	0.80	1.03
sLC	ILMN_1759084	INTS8	Homo sapiens integrator complex subunit 8 (INTS8)	1.61	1.13	1.52	0.86	1.02	0.92	1.26	0.94
sLC	ILMN_1684746	IPO11	Homo sapiens importin 11 (IPO11)	2.36	0.96	0.86	1.10	1.07	1.02	1.26	0.80
sLC	ILMN_2150187	KCTD18	Homo sapiens potassium channel tetramerisation domain containing 18	1.73	1.12	1.80	0.92	0.87	0.93	1.36	1.37
sLC	ILMN_2410771	KEAP1	Homo sapiens kelch-like ECH-associated protein 1 (KEAP1), transcrip	1.53	0.93	1.00	0.82	1.11	0.94	0.77	0.33
sLC	ILMN_1796749	KIF1C	Homo sapiens kinesin family member 1C (KIF1C)	2.09	0.89	0.89	1.12	1.12	1.06	1.21	0.84
sLC	ILMN_1727134	KLHDC5	Homo sapiens kelch domain containing 5 (KLHDC5)	2.48	1.00	0.91	1.11	1.13	1.05	1.06	0.90
sLC	ILMN_3256004	LOC100130003	PREDICTED: Homo sapiens misc_RNA (LOC100130003), miscRNA.	1.61	1.05	1.28	0.95	0.73	0.84	0.86	0.70
sLC	ILMN_3279219	LOC100132457	PREDICTED: Homo sapiens similar to Sm protein G (LOC100132457	1.72	1.07	1.34	0.91	0.79	0.76	0.86	0.71
sLC	ILMN_2205211	LOC134997	Homo sapiens peptidylprolyl isomerase A processed pseudogene (LOC	1.59	0.84	0.69	1.10	1.18	0.85	0.88	1.22
sLC	ILMN_1667298	LOC201229	PREDICTED: Homo sapiens hypothetical protein LOC201229, transcr	1.93	1.11	0.96	1.12	1.21	1.14	1.04	1.00
sLC	ILMN_1734880	LOC644128	PREDICTED: Homo sapiens hypothetical protein LOC644128 (LOC6	1.91	0.99	0.76	1.09	0.88	0.84	1.10	0.81
sLC	ILMN_1715525	LOC646038	PREDICTED: Homo sapiens similar to ADP-ribosylation factor 7 (LO	3.10	1.04	0.99	1.14	1.22	1.05	0.96	1.00
sLC	ILMN_3245074	LOC646916	PREDICTED: Homo sapiens hypothetical LOC646916 (LOC646916)	1.70	1.22	1.20	0.76	0.92	0.86	0.89	1.04
sLC	ILMN_1694752	LOC727726	PREDICTED: Homo sapiens similar to poly (ADP-ribose) glycohydro	1.98	1.04	1.19	1.13	1.07	1.09	1.15	1.04
sLC	ILMN_1691949	LOC728554	PREDICTED: Homo sapiens similar to THO complex 3 (LOC728554)	1.80	0.43	0.69	0.91	0.62	0.69	1.01	0.31
sLC	ILMN_3304898	LOC92755	PREDICTED: Homo sapiens misc_RNA (LOC92755), miscRNA.	1.53	1.21	1.15	0.92	1.14	1.03	1.01	0.83
sLC	ILMN_1691131	LSMD1	Homo sapiens LSM domain containing 1 (LSMD1)	1.75	1.01	1.17	1.02	1.13	1.10	0.98	1.14
sLC	ILMN_1665219	LTBP4	Homo sapiens latent transforming growth factor beta binding protein 4	1.99	0.89	0.86	1.25	1.29	1.36	1.08	0.93
sLC	ILMN_2388272	MED24	Homo sapiens mediator complex subunit 24 (MED24), transcript varia	2.26	0.69	0.84	1.07	0.73	0.95	1.09	0.99
sLC	ILMN_1749821	MED28	Homo sapiens mediator complex subunit 28 (MED28)	1.97	1.06	1.24	0.64	0.59	0.66	0.62	1.14
sLC	ILMN_1731184	MELK	Homo sapiens maternal embryonic leucine zipper kinase (MELK)	3.19	1.23	1.19	1.13	1.23	1.09	1.12	0.91
sLC	ILMN_1740512	MGC39900	PREDICTED: Homo sapiens hypothetical protein MGC39900 (MGC3	2.00	0.99	0.98	1.11	1.14	1.05	1.00	1.10
sLC	ILMN_1690807	MKL2	Homo sapiens MKL/myocardin-like 2 (MKL2)	1.54	1.00	0.99	1.00	1.04	1.00	1.03	1.00
sLC	ILMN_3237324	MMS19	Homo sapiens MMS19 nucleotide excision repair homolog (S. cerevisi	1.69	1.07	0.90	0.85	1.04	0.98	0.78	0.59
sLC	ILMN_2125562	MOBK1B	Homo sapiens MOB1, Mps One Binder kinase activator-like 1B (yeast	1.55	0.85	0.78	1.07	0.99	1.04	1.03	0.65
sLC	ILMN_3242011	MOBKL1B	Homo sapiens MOB1, Mps One Binder kinase activator-like 1B (yeast	2.03	0.89	1.01	1.10	0.99	0.84	1.07	0.52
sLC	ILMN_2121282	MRPS18B	Homo sapiens mitochondrial ribosomal protein S18B (MRPS18B), nuc	1.76	0.86	0.87	1.01	0.97	0.90	0.92	0.72
sLC	ILMN_1687359	MRPS23	Homo sapiens mitochondrial ribosomal protein S23 (MRPS23), nuclea	1.68	0.94	0.87	0.85	0.94	1.07	1.15	1.36
sLC	ILMN_2099249	MTERF	Homo sapiens mitochondrial transcription termination factor (MTERF)	1.86	1.06	1.01	1.28	1.27	1.20	1.33	0.95
sLC	ILMN_1679071	MTX3	Homo sapiens metaxin 3 (MTX3)	2.38	0.86	1.00	0.97	0.98	1.03	1.35	1.37
sLC	ILMN_2204297	MUDENG	Homo sapiens MU-2/AP1M2 domain containing, death-inducing (MU	2.21	1.14	1.18	1.05	1.09	1.04	1.25	0.92
sLC	ILMN_1741331	MUDENG	Homo sapiens MU-2/AP1M2 domain containing, death-inducing (MU	1.98	1.13	0.98	0.68	0.71	0.75	0.77	1.24
sLC	ILMN_1707534	NKRF	Homo sapiens NFKB repressing factor (NKRF)	1.52	1.13	0.74	0.80	1.17	0.87	0.78	1.61
sLC	ILMN_1656378	NMT2	Homo sapiens N-myristoyltransferase 2 (NMT2)	2.44	1.09	0.96	1.10	0.96	0.75	0.81	0.44
sLC	ILMN_1680353	NSF	Homo sapiens N-ethylmaleimide-sensitive factor (NSF)	1.66	0.90	0.74	0.89	0.94	0.81	1.15	0.68
sLC	ILMN_1739210	NSL1	Homo sapiens NSL1, MIND kinetochore complex component, homolo	1.59	0.60	1.15	0.81	0.56	0.67	1.38	0.50
sLC	ILMN_1791226	NXN	Homo sapiens nucleoredoxin (NXN)	3.99	1.00	0.98	1.21	1.18	1.08	1.12	1.00
sLC	ILMN_1730698	ODF2	Homo sapiens outer dense fiber of sperm tails 2 (ODF2), transcript var	1.75	0.85	0.69	0.86	0.81	0.81	1.17	1.15
sLC	ILMN_1722276	PAFAH1B1	Homo sapiens platelet-activating factor acetylhydrolase, isoform Ib, alp	1.87	0.83	0.77	0.84	0.62	0.56	1.29	0.73

sLC	ILMN_1724811	PARN	Homo sapiens poly(A)-specific ribonuclease (deadenylation nuclease) (	2.45	0.52	1.17	0.71	0.65	0.77	1.23	0.45
sLC	ILMN_1686871	PARP1	Homo sapiens poly (ADP-ribose) polymerase family, member 1 (PARP	1.82	0.64	0.60	0.82	0.87	0.77	1.05	0.53
sLC	ILMN_1678546	PEX11B	Homo sapiens peroxisomal biogenesis factor 11 beta (PEX11B)	2.60	0.90	0.53	0.88	0.71	0.79	1.23	1.09
sLC	ILMN_1763634	PEX14	Homo sapiens peroxisomal biogenesis factor 14 (PEX14)	1.72	1.01	1.02	1.08	1.11	1.07	1.09	0.94
sLC	ILMN_1728074	PHAX	Homo sapiens phosphorylated adaptor for RNA export (PHAX)	1.73	1.06	0.72	0.84	0.71	0.90	0.85	0.58
sLC	ILMN_1777644	PIB5PA	Homo sapiens phosphatidylinositol (4,5) bisphosphate 5-phosphatase, A	3.35	1.16	1.12	1.19	1.31	0.90	0.88	0.96
sLC	ILMN_1687896	PIK3C3	Homo sapiens phosphoinositide-3-kinase, class 3 (PIK3C3)	1.74	1.25	1.01	0.92	0.85	0.81	1.10	0.82
sLC	ILMN_2383383	PIR	Homo sapiens pirin (iron-binding nuclear protein) (PIR), transcript vari	1.58	1.00	0.98	1.05	0.99	1.11	1.03	1.00
sLC	ILMN_1719972	PLXNA3	Homo sapiens plexin A3 (PLXNA3)	1.68	0.94	0.94	1.12	0.79	1.02	1.13	0.47
sLC	ILMN_1740291	POLQ	Homo sapiens polymerase (DNA directed), theta (POLQ)	1.83	0.95	1.00	1.13	1.29	1.12	1.17	1.04
sLC	ILMN_1768273	POP1	Homo sapiens processing of precursor 1, ribonuclease P/MRP subunit (	1.51	1.00	1.03	1.18	1.22	1.22	1.12	1.00
sLC	ILMN_1802669	PPP3CB	Homo sapiens protein phosphatase 3 (formerly 2B), catalytic subunit, b	1.65	0.99	0.92	0.85	0.88	0.77	1.06	0.91
sLC	ILMN_1751887	PREP	Homo sapiens prolyl endopeptidase (PREP)	2.21	0.93	0.80	0.88	1.20	0.84	0.85	0.61
sLC	ILMN_1786021	PRKAB2	Homo sapiens protein kinase, AMP-activated, beta 2 non-catalytic subu	2.84	1.11	0.78	1.17	1.21	0.74	0.90	1.18
sLC	ILMN_2058512	PSMA2	Homo sapiens proteasome (prosome, macropain) subunit, alpha type, 2	1.94	0.93	0.96	0.90	0.75	0.87	0.87	1.01
sLC	ILMN_1776173	PSMD7	Homo sapiens proteasome (prosome, macropain) 26S subunit, non-ATF	1.58	0.85	0.85	0.85	0.69	0.71	1.07	1.12
sLC	ILMN_1763842	PTRH1	Homo sapiens peptidyl-tRNA hydrolase 1 homolog (S. cerevisiae) (PT	1.64	0.96	0.89	1.21	1.22	1.09	1.02	0.99
sLC	ILMN_1743049	PWP1	Homo sapiens PWP1 homolog (S. cerevisiae) (PWP1)	2.11	1.13	0.82	0.88	0.58	0.66	0.76	1.24
sLC	ILMN_1783846	RAPH1	Homo sapiens Ras association (RalGDS/AF-6) and pleckstrin homolog	2.44	1.09	0.88	1.20	1.25	0.90	1.00	0.54
sLC	ILMN_1714461	RNF14	Homo sapiens ring finger protein 14 (RNF14), transcript variant 3	2.21	1.06	1.02	1.14	1.19	1.19	1.26	0.89
sLC	ILMN_1699476	RPE	Homo sapiens ribulose-5-phosphate-3-epimerase (RPE), transcript vari	2.20	1.20	1.01	1.10	1.01	1.05	1.15	1.09
sLC	ILMN_1730082	RPUSD4	Homo sapiens RNA pseudouridylate synthase domain containing 4 (RP	1.67	0.76	0.71	1.09	0.83	0.92	0.78	1.26
sLC	ILMN_2059294	RTCD1	Homo sapiens RNA terminal phosphate cyclase domain 1 (RTCD1)	2.13	1.04	1.14	0.90	1.02	1.04	1.04	0.86
sLC	ILMN_2352036	RTN4	Homo sapiens reticulon 4 (RTN4), transcript variant 1	1.78	0.99	1.30	0.63	0.64	0.59	1.02	0.66
sLC	ILMN_1743747	RUSC1	Homo sapiens RUN and SH3 domain containing 1 (RUSC1)	1.89	0.80	0.92	1.23	1.03	0.92	1.04	0.65
sLC	ILMN_2413779	SEZ6L2	Homo sapiens seizure related 6 homolog (mouse)-like 2 (SEZ6L2), tra	2.41	1.02	1.00	1.07	1.14	1.22	1.12	0.97
sLC	ILMN_1665994	SILV	Homo sapiens silver homolog (mouse) (SILV)	2.99	1.01	1.01	1.03	1.04	1.12	1.02	1.00
sLC	ILMN_2142284	SLC25A43	Homo sapiens solute carrier family 25, member 43 (SLC25A43)	3.43	0.70	0.64	0.81	1.32	0.89	0.79	0.78
sLC	ILMN_1720311	SLC25A46	Homo sapiens solute carrier family 25, member 46 (SLC25A46)	2.63	0.95	1.03	0.96	1.10	0.99	1.08	1.41
sLC	ILMN_1799128	SLC30A9	Homo sapiens solute carrier family 30 (zinc transporter), member 9 (SL	2.25	1.22	1.13	0.80	0.72	0.70	1.13	0.93
sLC	ILMN_1706571	SLC35D2	Homo sapiens solute carrier family 35, member D2 (SLC35D2)	1.79	0.98	0.78	1.08	1.06	0.76	0.79	0.76
sLC	ILMN_1773643	SLC38A9	Homo sapiens solute carrier family 38, member 9 (SLC38A9)	2.14	1.00	1.05	0.76	0.62	0.66	1.08	0.46
sLC	ILMN_1694305	SMS	Homo sapiens spermine synthase (SMS)	1.54	0.67	1.05	1.13	1.05	1.09	0.93	0.41
sLC	ILMN_2413259	SOCS4	Homo sapiens suppressor of cytokine signaling 4 (SOCS4), transcript v	1.62	1.14	1.00	1.09	1.08	1.09	1.11	0.95
sLC	ILMN_2315694	STRADA	Homo sapiens STE20-related kinase adaptor alpha (STRADA), transcr	1.50	1.13	1.34	0.80	0.83	0.85	0.96	0.70
sLC	ILMN_1750896	STX17	Homo sapiens syntaxin 17 (STX17)	1.60	1.07	1.04	1.09	1.09	1.15	1.10	1.00
sLC	ILMN_1735093	TIMELESS	Homo sapiens timeless homolog (Drosophila) (TIMELESS)	1.72	1.16	1.08	0.91	0.95	0.97	0.98	0.49
sLC	ILMN_1785191	TMEM14A	Homo sapiens transmembrane protein 14A (TMEM14A)	3.07	1.00	1.00	1.16	0.99	0.99	1.23	1.50
sLC	ILMN_1685258	TMEM14B	Homo sapiens transmembrane protein 14B (TMEM14B)	2.67	0.88	0.84	1.06	1.15	0.78	0.88	1.07
sLC	ILMN_1813796	TMEM169	Homo sapiens transmembrane protein 169 (TMEM169)	4.38	0.72	0.97	1.47	1.42	1.08	0.97	0.50
sLC	ILMN_1748926	TMEM209	Homo sapiens transmembrane protein 209 (TMEM209)	1.80	0.98	0.95	1.19	1.04	1.10	1.26	0.82
sLC	ILMN_2388070	TMEM44	Homo sapiens transmembrane protein 44 (TMEM44), transcript varian	2.29	1.17	1.15	1.25	1.25	1.15	1.38	1.06
sLC	ILMN_1805812	TOR1A	Homo sapiens torsin family 1, member A (torsin A) (TOR1A)	1.73	0.75	0.85	0.73	0.61	0.76	1.20	1.37
sLC	ILMN_2214197	TP53INP1	Homo sapiens tumor protein p53 inducible nuclear protein 1 (TP53INP	2.24	1.20	1.07	0.74	0.96	0.66	1.06	0.67
sLC	ILMN_1714108	TP53INP1	Homo sapiens tumor protein p53 inducible nuclear protein 1 (TP53INP	1.66	1.16	0.78	0.85	0.96	0.97	1.08	0.81
sLC	ILMN_1669881	TSPAN13	Homo sapiens tetraspanin 13 (TSPAN13)	6.80	1.14	1.14	0.94	1.22	0.88	0.88	0.63
sLC	ILMN_1803850	TSPAN15	Homo sapiens tetraspanin 15 (TSPAN15)	1.54	1.14	0.94	1.09	0.97	1.07	1.27	0.78
sLC	ILMN_1810228	TTF2	Homo sapiens transcription termination factor, RNA polymerase II (TT	3.00	0.96	0.91	1.01	1.34	0.91	0.87	0.93
sLC	ILMN_1800612	VBP1	Homo sapiens von Hippel-Lindau binding protein 1 (VBP1)	2.25	0.90	0.79	1.00	1.08	0.95	1.06	1.53
sLC	ILMN_1653771	WDR63	Homo sapiens WD repeat domain 63 (WDR63)	1.62	1.00	1.02	1.03	1.04	1.01	1.05	1.00
sLC	ILMN_3237956	ZC3H12C	Homo sapiens zinc finger CCCH-type containing 12C (ZC3H12C)	2.85	1.02	1.00	0.93	0.97	0.73	0.79	0.79
sLC	ILMN_1684663	ZDHHC13	Homo sapiens zinc finger, DHHC-type containing 13 (ZDHHC13), tran	2.59	1.49	1.17	1.28	1.41	1.10	0.99	0.94
sLC	ILMN_1736901	ZDHHC23	Homo sapiens zinc finger, DHHC-type containing 23 (ZDHHC23)	1.75	1.05	1.05	1.23	1.20	1.09	1.13	1.00
sLC	ILMN_1798533	ZNF22	Homo sapiens zinc finger protein 22 (KOX 15) (ZNF22)	1.90	0.87	0.68	0.94	0.99	0.72	0.77	0.79
sLC	ILMN_1810127	ZNF789	Homo sapiens zinc finger protein 789 (ZNF789), transcript variant 2	2.31	1.03	1.07	1.09	1.13	1.05	1.07	1.22
sLC	ILMN_1653163	ZSCAN2	Homo sapiens zinc finger and SCAN domain containing 2 (ZSCAN2),	1.79	0.99	0.96	1.10	1.11	1.00	1.01	1.00
sCD14- DC	ILMN_2311674	ADCY6	Homo sapiens adenylate cyclase 6 (ADCY6), transcript variant 2	1.00	1.60	1.11	1.00	1.02	1.00	1.00	0.99
sCD14- DC	ILMN_1794875	AGPAT9	Homo sapiens 1-acylglycerol-3-phosphate O-acyltransferase 9 (AGPAT	0.91	3.03	0.88	1.19	0.68	0.88	0.78	0.78
sCD14- DC	ILMN_1752247	AKAP13	Homo sapiens A kinase (PRKA) anchor protein 13 (AKAP13), transcri	0.76	1.71	0.74	0.64	0.84	0.72	0.74	1.58
sCD14- DC	ILMN_2148847	AKIRIN2	Homo sapiens akirin 2 (AKIRIN2)	0.84	1.92	1.08	0.96	0.48	0.58	0.74	1.28
sCD14- DC	ILMN_1812926	ANTXR2	Homo sapiens anthrax toxin receptor 2 (ANTXR2)	0.92	1.75	1.44	1.19	0.88	1.00	0.93	0.87
sCD14- DC	ILMN_1810229	ARID4A	Homo sapiens AT rich interactive domain 4A (RBP1-like) (ARID4A), t	1.10	2.51	1.37	0.90	1.01	0.84	0.89	2.09
sCD14- DC	ILMN_1792825	ARIH2	Homo sapiens ariadne homolog 2 (Drosophila) (ARIH2)	1.09	1.61	1.01	1.10	0.78	0.83	0.85	0.86
sCD14- DC	ILMN_1680579	ATP2B4	Homo sapiens ATPase, Ca++ transporting, plasma membrane 4 (ATP2E	0.79	2.22	0.86	0.88	1.10	0.72	0.80	0.69
sCD14- DC	ILMN_2367753	ATP2B4	Homo sapiens ATPase, Ca++ transporting, plasma membrane 4 (ATP2E	0.98	2.27	1.15	1.14	1.31	0.74	0.82	1.00
sCD14- DC	ILMN_2153332	ATXN1	Homo sapiens ataxin 1 (ATXN1)	1.25	2.59	1.45	1.02	0.97	0.74	0.95	0.51
sCD14- DC	ILMN_2357438	AURKA	Homo sapiens aurora kinase A (AURKA), transcript variant 3	1.24	1.58	1.45	0.85	0.90	0.85	1.04	0.48
sCD14- DC	ILMN_3247802	BAT2L	Homo sapiens HLA-B associated transcript 2-like (BAT2L)	1.39	1.53	0.93	0.92	0.91	0.95	0.93	0.75
sCD14- DC	ILMN_1720850	BAZ2B	Homo sapiens bromodomain adjacent to zinc finger domain, 2B (BAZ2	1.42	1.99	0.78	0.79	0.90	0.79	1.04	0.61
sCD14- DC	ILMN_1681644	BIRC3	Homo sapiens baculoviral IAP repeat-containing 3 (BIRC3), transcript	0.82	2.13	1.24	1.04	1.28	1.34	0.72	0.38
sCD14- DC	ILMN_1667510	C12ORF65	Homo sapiens chromosome 12 open reading frame 65 (C12orf65)	1.24	1.68	1.29	1.12	1.24	1.12	0.93	0.82
sCD14- DC	ILMN_1742611	C1ORF52	Homo sapiens chromosome 1 open reading frame 52 (C1orf52)	0.95	2.02	1.44	0.92	1.02	0.83	0.97	0.90
sCD14- DC	ILMN_2184612	C3ORF52	Homo sapiens chromosome 3 open reading frame 52 (C3orf52)	0.97	3.79	1.44	0.99	0.99	0.93	0.92	0.65
sCD14- DC	ILMN_1776788	C5ORF41	Homo sapiens chromosome 5 open reading frame 41 (C5orf41)	0.75	2.48	0.73	1.06	1.08	1.09	1.05	1.00
sCD14- DC	ILMN_3248941	C6ORF225	Homo sapiens chromosome 6 open reading frame 225 (C6orf225)	1.34	1.68	1.36	1.06	1.02	1.03	1.06	0.81
sCD14- DC	ILMN_1657361	CBX7	Homo sapiens chromobox homolog 7 (CBX7)	0.91	1.98	1.35	0.97	0.96	0.71	0.99	0.42
sCD14- DC	ILMN_1686135	CCDC45	Homo sapiens coiled-coil domain containing 45 (CCDC45)	0.91	2.25	1.34	0.74	0.71	0.69	0.80	1.48
sCD14- DC	ILMN_1761464	CD74	Homo sapiens CD74 molecule, major histocompatibility complex, clas	1.20	1.76	1.01	1.17	1.23	0.98	0.97	0.99
sCD14- DC	ILMN_1691418	CDRT4	Homo sapiens CMT1A duplicated region transcript 4 (CDRT4)	0.93	1.64	1.51	1.08	1.00	0.82	1.27	0.52
sCD14- DC	ILMN_2339284	CHD2	Homo sapiens chromodomain helicase DNA binding protein 2 (CHD2)	0.91	2.08	1.03	1.33	1.35	0.96	0.99	2.01
sCD14- DC	ILMN_2063586	CLIC4	Homo sapiens chloride intracellular channel 4 (CLIC4), nuclear gene e	1.43	1.70	1.59	0.88	0.90	0.81	0.94	0.68
sCD14- DC	ILMN_2370624	COL13A1	Homo sapiens collagen, type XIII, alpha 1 (COL13A1), transcript varia	1.10	1.51	1.45	1.13	1.06	1.02	1.15	0.99
sCD14- DC	ILMN_1728180	CROP	Homo sapiens cisplatin resistance-associated overexpressed protein (C	0.93	2.10	1.29	0.93	0.97	0.90	0.93	1.11
sCD14- DC	ILMN_1719290	CRYZL1	Homo sapiens crystallin, zeta (quinone reductase)-like 1 (CRYZL1), tra	1.00	1.65	1.15	0.80	0.77	0.75	0.99	0.57
sCD14- DC	ILMN_2046470	DAAM1	Homo sapiens dishevelled associated activator of morphogenesis 1 (DA	1.38	2.75	1.47	1.30	1.35	1.01	0.95	0.82
sCD14- DC	ILMN_1809285	DCP1A	Homo sapiens DCP1 decapping enzyme homolog A (S. cerevisiae) (DC	0.96	1.60	0.95	0.94	0.90	0.98	0.85	2.10
sCD14- DC	ILMN_2277523	DIP2A	Homo sapiens DIP2 disco-interacting protein 2 homolog A (Drosophila	1.06	1.88	1.05	0.93	1.08	0.92	1.04	0.64
sCD14- DC	ILMN_1758629	DONSON	Homo sapiens downstream neighbor of SON (DONSON)	1.09	2.04	1.12	1.19	1.11	1.00	1.04	0.97
sCD14- DC	ILMN_1679262	DPYSL3	Homo sapiens dihydropyrimidinase-like 3 (DPYSL3)	0.45	2.06	1.58	0.92	1.14	1.26	1.26	0.45
sCD14- DC	ILMN_1798210	E2F7	Homo sapiens E2F transcription factor 7 (E2F7)	0.95	4.21	1.29	0.87	0.93	0.84	0.71	0.68
sCD14- DC	ILMN_1724984	EIF2AK3	Homo sapiens eukaryotic translation initiation factor 2-alpha kinase 3 (	1.03	2.05	1.02	1.20	1.10	1.03	0.97	1.67
sCD14- DC	ILMN_1708369	EPS15L1	Homo sapiens epidermal growth factor receptor pathway substrate 15-l	1.09	3.58	1.16	1.00	1.01	1.01	1.00	1.43
sCD14- DC	ILMN_1664068	ERGIC1	Homo sapiens endoplasmic reticulum-golgi intermediate compartment	1.06	2.64	1.32	1.04	0.99	1.08	1.02	0.98
sCD14- DC	ILMN_1651504	FAM193A	Homo sapiens family with sequence similarity 193, member A (FAM19	1.02	1.80	0.92	0.92	0.76	0.75	0.87	1.08
sCD14- DC	ILMN_1714349	GLCE	Homo sapiens glucuronic acid epimerase (GLCE)	1.08	1.69	1.33	0.78	0.91	0.75	1.26	0.59
sCD14- DC	ILMN_1713756	GLUD1	Homo sapiens glutamate dehydrogenase 1 (GLUD1)	0.88	1.54	1.12	0.92	0.71	0.74	1.07	1.25
sCD14- DC	ILMN_1683204	GMEB2	Homo sapiens glucocorticoid modulatory element binding protein 2 (G	0.82	1.73	1.25	1.03	0.96	0.86	0.83	1.21
sCD14- DC	ILMN_1736757	GNPTAB	Homo sapiens N-acetylglucosamine-1-phosphate transferase, alpha and	1.24	1.55	0.62	1.08	0.96	0.71	0.77	0.69

sCD14- DC	ILMN_1716821	GORASP1	Homo sapiens golgi reassembly stacking protein 1, 65kDa (GORASP1	0.83	1.63	0.65	0.95	0.89	0.75	1.01	0.96
sCD14- DC	ILMN_1685115	HEXIM1	Homo sapiens hexamethylene bis-acetamide inducible 1 (HEXIM1)	0.93	2.19	1.23	1.11	0.93	0.85	0.83	0.70
sCD14- DC	ILMN_3270641	HNRNPH3	Homo sapiens heterogeneous nuclear ribonucleoprotein H3 (2H9) (HN	1.30	1.95	1.48	0.94	0.96	0.88	1.06	1.33
sCD14- DC	ILMN_1831106	HS.19193	PREDICTED: Homo sapiens hypothetical LOC400043 (LOC400043),	1.12	3.51	1.37	1.02	0.98	1.06	1.15	1.19
sCD14- DC	ILMN_1901304	HS.238996	Homo sapiens mRNA; cDNA DKFZp761E11121 (from clone DKFZp7	1.01	1.84	1.43	1.35	1.32	1.05	1.00	0.98
sCD14- DC	ILMN_1873677	HS.31037	Homo sapiens cDNA FLJ35556 fis, clone SPLEN2004844	0.93	1.89	1.45	1.37	0.94	1.04	1.17	0.70
sCD14- DC	ILMN_1907042	HS.445414	Homo sapiens cDNA FLJ41270 fis, clone BRAMY2036387	0.86	2.25	1.24	1.03	0.95	1.11	0.97	0.92
sCD14- DC	ILMN_1883937	HS.552282	Homo sapiens mRNA; cDNA DKFZp313P0125 (from clone DKFZp31	1.03	1.70	1.38	0.98	1.05	1.28	1.14	0.97
sCD14- DC	ILMN_2237746	ING3	Homo sapiens inhibitor of growth family, member 3 (ING3), transcript	1.19	1.77	1.54	0.80	0.89	0.81	0.87	2.14
sCD14- DC	ILMN_2169439	ITGAV	Homo sapiens integrin, alpha V (vitronectin receptor, alpha polypeptide	1.03	2.43	1.35	0.88	0.79	0.91	1.09	0.39
sCD14- DC	ILMN_1722532	JMJD1A	Homo sapiens jumonji domain containing 1A (JMJD1A)	0.77	2.00	1.23	0.79	1.01	0.75	1.17	1.03
sCD14- DC	ILMN_1730917	KMO	Homo sapiens kynurenine 3-monooxygenase (kynurenine 3-hydroxylas	0.79	2.39	1.18	0.90	0.78	0.90	1.27	0.45
sCD14- DC	ILMN_1689817	LCOR	Homo sapiens ligand dependent nuclear receptor corepressor (LCOR)	0.90	2.00	0.86	0.98	1.01	0.97	0.98	1.95
sCD14- DC	ILMN_2062381	LCOR	Homo sapiens ligand dependent nuclear receptor corepressor (LCOR)	0.86	1.96	1.28	0.74	0.78	0.81	0.95	1.77
sCD14- DC	ILMN_1727361	LEMD3	Homo sapiens LEM domain containing 3 (LEMD3)	0.92	1.56	0.68	1.10	0.53	0.63	0.68	1.01
sCD14- DC	ILMN_3187254	LOC100128016	PREDICTED: Homo sapiens misc_RNA (LOC100128016), miscRNA.	1.04	2.01	1.28	0.88	0.84	0.90	0.83	0.97
sCD14- DC	ILMN_3241692	LOC100129668	PREDICTED: Homo sapiens hypothetical protein LOC100129668 (LO	1.09	1.56	0.85	1.19	1.19	1.13	1.06	0.64
sCD14- DC	ILMN_3200322	LOC387791	PREDICTED: Homo sapiens misc_RNA (LOC387791), miscRNA.	1.04	1.55	1.10	1.12	0.91	0.95	0.91	0.92
sCD14- DC	ILMN_1665290	LOC643995	PREDICTED: Homo sapiens similar to Importin alpha-2 subunit (Kary	1.02	3.44	1.54	0.89	0.97	0.89	0.85	1.03
sCD14- DC	ILMN_3237368	LOC644132	PREDICTED: Homo sapiens misc_RNA (LOC644132), miscRNA.	0.84	1.54	1.13	1.21	0.88	0.75	0.89	0.81
sCD14- DC	ILMN_1712347	LOC644422	PREDICTED: Homo sapiens misc_RNA (LOC644422), miscRNA.	0.94	1.77	1.15	0.97	1.03	1.06	0.94	1.81
sCD14- DC	ILMN_3246060	LOC654433	Homo sapiens hypothetical LOC654433 (LOC654433), non-coding RN	1.05	1.60	1.17	1.11	1.07	1.20	1.30	0.81
sCD14- DC	ILMN_2214997	LRRFIP1	Homo sapiens leucine rich repeat (in FLII) interacting protein 1 (LRRF	0.86	1.64	1.08	0.87	1.01	0.93	0.80	1.27
sCD14- DC	ILMN_2301624	MACF1	Homo sapiens microtubule-actin crosslinking factor 1 (MACF1), transc	1.26	2.25	1.17	0.81	0.61	0.75	1.02	0.74
sCD14- DC	ILMN_1811367	MAT2B	Homo sapiens methionine adenosyltransferase II, beta (MAT2B), trans	1.48	1.54	1.23	0.72	0.63	0.57	0.85	0.70
sCD14- DC	ILMN_3248324	MGC16384	PREDICTED: Homo sapiens hypothetical protein MGC16384 (MGC1	1.14	1.52	1.15	1.09	1.12	1.06	1.06	0.90
sCD14- DC	ILMN_3245973	MSL1	Homo sapiens male-specific lethal 1 homolog (Drosophila) (MSL1)	0.83	1.98	1.24	0.96	0.98	0.76	1.04	0.65
sCD14- DC	ILMN_1656111	MYLIP	Homo sapiens myosin regulatory light chain interacting protein (MYLI	0.52	2.39	1.09	0.60	0.47	0.50	0.56	0.77
sCD14- DC	ILMN_1724718	NCK2	Homo sapiens NCK adaptor protein 2 (NCK2), transcript variant 2	1.43	3.53	1.55	0.74	1.06	0.74	0.65	0.80
sCD14- DC	ILMN_1690325	NFYA	Homo sapiens nuclear transcription factor Y, alpha (NFYA), transcript v	1.07	1.74	1.24	1.05	1.11	1.07	1.07	0.92
sCD14- DC	ILMN_1728779	NGLY1	Homo sapiens N-glycanase 1 (NGLY1)	0.74	2.26	1.37	1.18	0.74	0.82	0.92	2.09
sCD14- DC	ILMN_1673962	NUP205	Homo sapiens nucleoporin 205kDa (NUP205)	1.38	1.70	0.88	0.63	0.56	0.64	0.79	0.89
sCD14- DC	ILMN_2358652	NXF1	Homo sapiens nuclear RNA export factor 1 (NXF1), transcript variant	0.65	1.95	1.01	0.71	0.63	0.57	0.69	0.82
sCD14- DC	ILMN_1708095	PANK2	Homo sapiens pantothenate kinase 2 (PANK2), transcript variant 3	1.24	1.68	1.11	0.99	0.83	0.77	0.76	0.96
sCD14- DC	ILMN_1781388	PGM5	Homo sapiens phosphoglucomutase 5 (PGM5)	1.03	3.27	1.99	0.96	0.96	1.23	1.05	0.65
sCD14- DC	ILMN_1736015	PHF17	Homo sapiens PHD finger protein 17 (PHF17), transcript variant S	1.24	2.14	1.71	1.55	1.14	0.97	0.85	1.44
sCD14- DC	ILMN_1788689	PHIP	Homo sapiens pleckstrin homology domain interacting protein (PHIP)	1.03	1.81	1.02	0.98	0.93	0.71	0.80	1.48
sCD14- DC	ILMN_1815023	PIM1	Homo sapiens pim-1 oncogene (PIM1)	0.82	1.84	1.20	0.77	0.73	0.67	0.89	0.94
sCD14- DC	ILMN_1707748	PIM3	PREDICTED: Homo sapiens pim-3 oncogene (PIM3)	0.85	1.66	1.06	1.06	1.08	0.93	0.69	1.89
sCD14- DC	ILMN_1786601	PLAGL2	Homo sapiens pleiomorphic adenoma gene-like 2 (PLAGL2)	0.86	1.84	1.10	0.74	0.87	0.82	1.09	1.13
sCD14- DC	ILMN_1662839	PLEKHA1	Homo sapiens pleckstrin homology domain containing, family A (phos	1.51	1.76	1.50	1.03	1.18	0.82	0.74	0.40
sCD14- DC	ILMN_1696713	POLA2	Homo sapiens polymerase (DNA directed), alpha 2 (70kD subunit) (PO	0.93	1.56	1.29	0.69	0.92	1.02	0.82	0.48
sCD14- DC	ILMN_1660282	POPDC2	Homo sapiens popeye domain containing 2 (POPDC2)	1.08	2.02	1.22	0.99	1.07	1.06	1.00	0.99
sCD14- DC	ILMN_1717477	PSD3	Homo sapiens pleckstrin and Sec7 domain containing 3 (PSD3), transc	1.11	1.50	1.03	1.03	1.03	1.02	1.01	0.89
sCD14- DC	ILMN_3237679	PTAR1	Homo sapiens protein prenyltransferase alpha subunit repeat containing	1.13	2.00	1.49	0.94	0.91	0.90	1.03	1.46
sCD14- DC	ILMN_2401155	PUM1	Homo sapiens pumilio homolog 1 (Drosophila) (PUM1), transcript var	0.87	1.71	1.17	0.95	0.73	0.91	0.78	0.81
sCD14- DC	ILMN_1670666	RAB12	Homo sapiens RAB12, member RAS oncogene family (RAB12)	1.22	2.15	1.02	0.91	0.89	0.95	0.94	2.11
sCD14- DC	ILMN_1727045	RASGRP3	Homo sapiens RAS guanyl releasing protein 3 (calcium and DAG-regu	0.76	2.14	1.24	1.11	0.82	0.81	0.97	0.30
sCD14- DC	ILMN_2381197	RNF19A	Homo sapiens ring finger protein 19A (RNF19A), transcript variant 2	1.07	2.15	1.27	0.91	1.02	0.85	0.96	0.99
sCD14- DC	ILMN_2117223	ROD1	Homo sapiens ROD1 regulator of differentiation 1 (S. pombe) (ROD1)	1.04	1.55	1.05	0.87	0.80	0.63	0.92	0.92
sCD14- DC	ILMN_3243461	RPRD1B	Homo sapiens regulation of nuclear pre-mRNA domain containing 1B (	0.98	1.67	1.14	1.07	1.01	0.97	1.20	2.45
sCD14- DC	ILMN_2334303	SEC24B	Homo sapiens SEC24 family, member B (S. cerevisiae) (SEC24B), tran	1.36	1.58	1.25	0.90	0.81	0.74	0.94	1.89
sCD14- DC	ILMN_1724959	SEC31A	Homo sapiens SEC31 homolog A (S. cerevisiae) (SEC31A), transcript	1.28	1.56	1.08	1.09	1.24	0.97	1.07	1.04
sCD14- DC	ILMN_1689007	SFRS14	Homo sapiens splicing factor, arginine/serine-rich 14 (SFRS14), transc	0.82	1.81	1.14	1.07	1.10	1.14	1.23	1.04
sCD14- DC	ILMN_1747020	SGK3	Homo sapiens serum/glucocorticoid regulated kinase family, member 3	0.89	1.76	1.34	0.77	0.82	0.75	1.25	0.73
sCD14- DC	ILMN_1715742	SLC22A1	Homo sapiens solute carrier family 22 (organic cation transporter), mer	1.05	1.85	1.05	1.06	1.15	1.06	1.05	0.99
sCD14- DC	ILMN_1768798	SPAG9	Homo sapiens sperm associated antigen 9 (SPAG9), transcript variant 2	1.10	2.53	1.17	1.23	1.08	1.11	1.05	0.93
sCD14- DC	ILMN_1799104	SPAG9	Homo sapiens sperm associated antigen 9 (SPAG9)	0.80	2.80	1.40	0.98	0.71	0.58	0.82	0.89
sCD14- DC	ILMN_2263718	SPAG9	Homo sapiens sperm associated antigen 9 (SPAG9)	0.99	2.66	1.39	0.87	0.63	0.60	0.72	1.14
sCD14- DC	ILMN_1785202	STAT4	Homo sapiens signal transducer and activator of transcription 4 (STAT4	1.67	1.81	1.20	0.55	0.82	0.74	0.69	0.83
sCD14- DC	ILMN_1737535	TAF4	Homo sapiens TAF4 RNA polymerase II, TATA box binding protein (T	0.80	1.77	0.99	0.87	0.87	0.96	1.13	0.64
sCD14- DC	ILMN_1803941	TBC1D15	Homo sapiens TBC1 domain family, member 15 (TBC1D15)	0.82	1.70	0.91	1.29	0.82	0.60	0.78	0.82
sCD14- DC	ILMN_1703891	TBC1D9	Homo sapiens TBC1 domain family, member 9 (with GRAM domain)	1.35	1.61	1.17	1.24	1.22	1.17	1.16	0.94
sCD14- DC	ILMN_1657983	TERF2IP	Homo sapiens telomeric repeat binding factor 2, interacting protein (TE	0.81	1.68	0.81	0.83	0.84	0.71	0.88	0.88
sCD14- DC	ILMN_2318638	TGIF1	Homo sapiens TGFB-induced factor homeobox 1 (TGIF1), transcript v	1.08	2.26	1.44	0.86	0.64	0.71	0.76	0.73
sCD14- DC	ILMN_1724863	TICAM1	Homo sapiens toll-like receptor adaptor molecule 1 (TICAM1), transcr	0.63	1.80	1.17	1.04	0.60	0.60	0.71	0.68
sCD14- DC	ILMN_1748884	TOB2	Homo sapiens transducer of ERBB2, 2 (TOB2)	1.04	2.02	1.49	1.07	0.92	0.90	0.87	0.71
sCD14- DC	ILMN_3247223	TPBG	Homo sapiens trophoblast glycoprotein (TPBG)	1.14	1.72	1.09	1.06	1.06	1.06	1.03	1.00
sCD14- DC	ILMN_1731043	TRA2A	Homo sapiens transformer 2 alpha homolog (Drosophila) (TRA2A)	0.72	2.68	1.31	0.81	0.75	0.87	0.77	1.00
sCD14- DC	ILMN_1682316	TRIM33	Homo sapiens tripartite motif-containing 33 (TRIM33), transcript varia	1.16	2.65	1.24	0.90	0.78	0.70	1.04	1.31
sCD14- DC	ILMN_1783771	UBE2Z	PREDICTED: Homo sapiens ubiquitin-conjugating enzyme E2Z (putat	0.94	2.06	1.17	1.27	1.07	1.40	0.95	0.76
sCD14- DC	ILMN_1780887	USP21	Homo sapiens ubiquitin specific peptidase 21 (USP21), transcript varia	0.93	2.00	1.10	0.85	1.02	0.89	1.08	0.95
sCD14- DC	ILMN_1739454	USP34	Homo sapiens ubiquitin specific peptidase 34 (USP34)	1.15	1.86	1.10	0.99	1.05	1.04	0.97	1.06
sCD14- DC	ILMN_2414826	VPS13A	Homo sapiens vacuolar protein sorting 13 homolog A (S. cerevisiae) (V	1.14	1.65	1.10	1.06	1.08	1.02	1.02	0.95
sCD14- DC	ILMN_1745852	WDR33	Homo sapiens WD repeat domain 33 (WDR33), transcript variant 3	1.12	2.55	1.15	0.94	1.07	0.95	0.90	1.36
sCD14- DC	ILMN_1807379	WHSC1L1	Homo sapiens Wolf-Hirschhorn syndrome candidate 1-like 1 (WHSC1	0.90	2.25	1.47	1.01	1.24	1.00	0.95	0.78
sCD14+ DC	ILMN_1653203	EFEMP2	Homo sapiens EGF-containing fibulin-like extracellular matrix protein	0.87	1.15	1.54	1.14	0.85	0.94	0.78	0.35
sCD14+ DC	ILMN_1791635	LOC652844	PREDICTED: Homo sapiens similar to phosphodiesterase 4D interacti	1.00	1.17	1.51	1.44	1.13	1.04	1.14	0.96
sCD14+ DC	ILMN_1748751	NLF2	PREDICTED: Homo sapiens nuclear localized factor 2 (NLF2)	0.87	2.10	3.43	1.31	0.93	1.07	1.06	0.88
sCD14+ DC	ILMN_1697220	NT5E	Homo sapiens 5'-nucleotidase, ecto (CD73) (NT5E)	1.21	1.18	3.07	0.86	0.89	0.94	1.16	0.98
sCD14+ DC	ILMN_1751464	TNFSF9	Homo sapiens tumor necrosis factor (ligand) superfamily, member 9 (T	0.97	1.39	2.05	0.98	0.94	0.94	1.02	1.80
vLC	ILMN_2389013	ADRM1	Homo sapiens adhesion regulating molecule 1 (ADRM1), transcript va	1.11	1.03	0.96	1.57	1.09	1.09	1.09	0.99
vLC	ILMN_2179726	C16ORF93	Homo sapiens chromosome 16 open reading frame 93 (C16orf93)	0.98	0.93	0.74	1.83	1.15	1.10	0.87	1.27
vLC	ILMN_1669123	C1ORF187	Homo sapiens chromosome 1 open reading frame 187 (C1orf187)	1.19	1.04	1.06	1.82	1.07	1.24	1.03	1.00
vLC	ILMN_1711573	CD96	Homo sapiens CD96 molecule (CD96), transcript variant 1	1.22	1.04	1.13	1.50	1.41	1.50	1.40	1.00
vLC	ILMN_1782439	CNN3	Homo sapiens calponin 3, acidic (CNN3)	1.31	0.91	0.80	2.13	1.51	1.04	0.82	0.37
vLC	ILMN_1758128	CYGB	Homo sapiens cytoglobin (CYGB)	0.98	1.19	0.99	1.58	0.91	1.38	1.04	0.71
vLC	ILMN_1711650	KCNE1L	Homo sapiens KCNE1-like (KCNE1L)	0.97	1.16	1.36	2.06	1.52	1.32	0.95	0.99
vLC	ILMN_1801632	KRT5	Homo sapiens keratin 5 (KRT5)	1.03	1.02	1.05	2.76	1.87	1.22	1.55	1.00
vLC	ILMN_1668194	LMTK3	PREDICTED: Homo sapiens lemur tyrosine kinase 3 (LMTK3)	1.08	0.95	1.01	1.67	1.40	1.06	1.31	0.90
vLC	ILMN_1715024	LSS	Homo sapiens lanosterol synthase (2,3-oxidosqualene-lanosterol cyclas	1.17	0.98	0.87	1.65	1.40	1.15	1.03	0.93
vLC	ILMN_1713638	NIPAL4	Homo sapiens NIPA-like domain containing 4 (NIPAL4)	1.18	1.67	2.16	2.19	1.60	1.35	1.43	1.05
vLC	ILMN_1725852	S100A2	Homo sapiens S100 calcium binding protein A2 (S100A2)	1.08	1.04	1.20	2.83	1.62	1.37	1.38	1.01
vLC	ILMN_2350801	SLC25A29	Homo sapiens solute carrier family 25, member 29 (SLC25A29), nucle	0.89	1.34	1.07	1.63	1.13	1.14	0.89	1.00
vLC	ILMN_1730740	VSIG8	Homo sapiens V-set and immunoglobulin domain containing 8 (VSIG8	1.01	0.96	1.01	2.77	2.51	1.19	1.54	0.87
vLC	ILMN_1696276	ZNF653	Homo sapiens zinc finger protein 653 (ZNF653)	1.05	0.92	0.89	1.53	1.38	1.18	0.87	0.77
vCD14- DC	ILMN_1664828	APOBEC3H	Homo sapiens apolipoprotein B mRNA editing enzyme, catalytic polyp	1.61	1.00	1.04	1.07	1.91	1.31	0.88	0.63
vCD14- DC	ILMN_1701243	C10ORF2	Homo sapiens chromosome 10 open reading frame 2 (C10orf2)	1.53	1.03	1.08	1.25	1.51	1.23	1.25	1.00

vCD14- DC	ILMN_1651496	HIST1H2BD	Homo sapiens histone cluster 1, H2bd (HIST1H2BD), transcript varian	1.37	1.31	1.17	1.66	1.64	0.95	0.88	0.56
vCD14- DC	ILMN_1699521	KIAA1641	PREDICTED: Homo sapiens KIAA1641, transcript variant 7 (KIAA16	1.75	0.97	0.89	1.71	1.74	1.45	1.50	1.47
vCD14- DC	ILMN_2249018	LOC389816	Homo sapiens cytokeratin associated protein (LOC389816)	1.25	0.99	1.10	1.19	1.61	1.08	1.08	0.95
vCD14- DC	ILMN_1652072	MGC42105	Homo sapiens serine/threonine-protein kinase NIM1 (MGC42105)	1.18	1.00	1.03	1.15	1.56	1.04	1.00	0.97
vCD14- DC	ILMN_2348090	MRPL55	Homo sapiens mitochondrial ribosomal protein L55 (MRPL55), nuclea	1.04	1.13	0.81	1.28	1.54	1.17	0.81	1.10
vCD14- DC	ILMN_1738147	NES	Homo sapiens nestin (NES)	1.01	1.02	1.03	1.17	1.50	1.14	1.03	1.00
vCD14- DC	ILMN_2141157	RANBP9	Homo sapiens RAN binding protein 9 (RANBP9)	1.43	1.11	1.17	1.32	1.50	1.01	1.12	0.98
vCD14- DC	ILMN_3249059	SNHG12	Homo sapiens small nucleolar RNA host gene 12 (non-protein coding)	0.91	1.22	1.13	1.32	1.73	1.04	1.11	0.82
vCD14- DC	ILMN_1751963	ZCWPW1	Homo sapiens zinc finger, CW type with PWWP domain 1 (ZCWPW1	1.18	0.76	0.43	1.35	1.89	1.03	0.93	0.65
vCD14- DC	ILMN_1684591	ZNF434	Homo sapiens zinc finger protein 434 (ZNF434)	1.17	0.86	1.11	1.40	1.62	1.07	1.16	0.80
vCD14+ DC	ILMN_1687556	DAPL1	Homo sapiens death associated protein-like 1 (DAPL1)	1.05	1.03	1.09	1.83	1.78	1.54	1.27	1.00
vCD14+ DC	ILMN_1792497	HRB	Homo sapiens HIV-1 Rev binding protein (HRB)	0.91	1.10	1.17	1.32	1.15	1.58	1.31	0.87
vCD14+ DC	ILMN_2216265	LONP2	Homo sapiens lon peptidase 2, peroxisomal (LONP2)	0.71	0.77	0.83	1.15	1.13	1.67	1.41	0.60
vCD14+ DC	ILMN_2061435	MEG3	Homo sapiens maternally expressed 3 (non-protein coding) (MEG3), tr	0.98	0.96	1.01	1.59	1.20	1.59	1.15	0.94
vCD14+ DC	ILMN_1782331	TDG	Homo sapiens thymine-DNA glycosylase (TDG)	0.82	0.87	0.82	1.27	1.44	1.53	1.02	0.57
vCD14+ DC	ILMN_1710268	ZNF385D	Homo sapiens zinc finger protein 385D (ZNF385D)	0.99	0.98	1.06	1.46	1.21	1.58	1.22	0.96
vMF	ILMN_2061446	AADACL1	Homo sapiens arylacetamide deacetylase-like 1 (AADACL1)	0.97	0.93	0.85	1.28	1.02	1.09	1.77	0.98
vMF	ILMN_1698777	ADCK1	Homo sapiens aarF domain containing kinase 1 (ADCK1)	1.05	1.00	1.00	1.00	1.00	1.09	1.75	1.39
vMF	ILMN_1663132	ADCK2	Homo sapiens aarF domain containing kinase 2 (ADCK2)	0.75	0.86	0.66	1.05	0.85	0.91	1.60	0.85
vMF	ILMN_1794825	ALDH3A2	Homo sapiens aldehyde dehydrogenase 3 family, member A2 (ALDH3	0.84	0.75	1.31	0.67	0.72	0.90	1.98	0.46
vMF	ILMN_1724040	ANKRD57	Homo sapiens ankyrin repeat domain 57 (ANKRD57)	0.74	0.92	2.52	0.91	0.92	1.02	2.94	0.71
vMF	ILMN_1803779	AP2A2	Homo sapiens adaptor-related protein complex 2, alpha 2 subunit (AP2	1.28	1.07	0.91	1.13	1.00	1.19	1.53	0.90
vMF	ILMN_1651574	AQP3	Homo sapiens aquaporin 3 (Gill blood group) (AQP3)	1.11	1.06	1.21	2.01	1.97	1.49	1.73	1.00
vMF	ILMN_1727390	ATPAF2	Homo sapiens ATP synthase mitochondrial F1 complex assembly facto	1.24	1.00	1.09	1.13	1.11	1.15	1.69	1.01
vMF	ILMN_1656045	BRUNOL6	Homo sapiens bruno-like 6, RNA binding protein (Drosophila) (BRUN	1.01	0.95	0.86	1.06	1.01	1.09	2.51	0.97
vMF	ILMN_2213297	C11ORF54	Homo sapiens chromosome 11 open reading frame 54 (C11orf54)	1.36	0.98	0.94	1.13	1.16	1.17	1.55	0.75
vMF	ILMN_1707137	C17ORF97	Homo sapiens chromosome 17 open reading frame 97 (C17orf97)	1.60	0.74	0.85	1.35	1.07	1.17	1.82	1.29
vMF	ILMN_1748916	C18ORF55	Homo sapiens chromosome 18 open reading frame 55 (C18orf55)	1.33	0.92	0.98	1.05	1.03	1.13	1.97	0.89
vMF	ILMN_2182531	C18ORF55	Homo sapiens chromosome 18 open reading frame 55 (C18orf55)	1.29	0.49	0.90	0.77	0.59	0.72	2.14	0.34
vMF	ILMN_1718712	C20ORF177	Homo sapiens chromosome 20 open reading frame 177 (C20orf177)	1.39	0.87	0.88	1.16	1.07	1.12	1.52	0.79
vMF	ILMN_1655572	C6ORF59	PREDICTED: Homo sapiens chromosome 6 open reading frame 59 (C	0.99	0.99	1.07	1.02	1.03	1.18	2.32	0.99
vMF	ILMN_3241996	C6ORF59	Homo sapiens chromosome 6 open reading frame 59 (C6orf59), non-co	0.99	0.99	1.00	1.24	1.11	1.09	1.76	1.05
vMF	ILMN_1777740	C8ORF55	Homo sapiens chromosome 8 open reading frame 55 (C8orf55)	1.13	0.87	0.76	1.51	1.17	1.06	2.01	0.81
vMF	ILMN_2365307	CD276	Homo sapiens CD276 molecule (CD276), transcript variant 1 XM_945	1.11	1.01	1.11	0.98	0.97	1.13	1.67	1.00
vMF	ILMN_2143685	CLDN7	Homo sapiens claudin 7 (CLDN7)	1.01	0.97	0.94	2.85	2.17	1.50	2.35	0.96
vMF	ILMN_1653708	CORO1B	Homo sapiens coronin, actin binding protein, 1B (CORO1B), transcrip	1.08	0.91	0.81	1.39	1.10	1.13	1.64	0.87
vMF	ILMN_2198185	CXORF12	Homo sapiens chromosome X open reading frame 12 (CXorf12)	1.05	1.15	1.02	1.40	1.06	1.21	1.85	1.00
vMF	ILMN_2355463	CYFIP1	Homo sapiens cytoplasmic FMR1 interacting protein 1 (CYFIP1), trans	1.34	0.96	0.97	0.80	0.69	0.86	1.98	0.57
vMF	ILMN_1760509	EOMES	Homo sapiens eomesodermin homolog (Xenopus laevis) (EOMES)	1.19	1.05	1.16	1.65	1.54	1.29	1.58	1.00
vMF	ILMN_1770245	EPB41L5	Homo sapiens erythrocyte membrane protein band 4.1 like 5 (EPB41L5	1.01	1.01	1.02	1.09	1.02	1.14	1.66	1.00
vMF	ILMN_2367384	EPHB2	Homo sapiens EPH receptor B2 (EPHB2), transcript variant 2	1.00	0.97	0.94	0.97	0.94	1.25	2.14	0.95
vMF	ILMN_1781536	FAH	Homo sapiens fumarylacetoacetate hydrolase (fumarylacetoacetase) (F	0.96	1.00	1.18	0.87	0.94	1.30	2.43	0.95
vMF	ILMN_1804863	FKBP15	Homo sapiens FK506 binding protein 15, 133kDa (FKBP15)	1.02	0.95	1.00	0.97	0.96	1.47	3.47	1.17
vMF	ILMN_1773553	FLJ46906	PREDICTED: Homo sapiens hypothetical gene supported by AK12887	0.92	0.91	1.24	1.21	0.93	1.51	2.84	1.08
vMF	ILMN_1678961	FRMD4A	Homo sapiens FERM domain containing 4A (FRMD4A)	0.97	0.96	0.95	1.12	0.98	1.23	2.78	0.92
vMF	ILMN_1743367	FZD4	Homo sapiens frizzled homolog 4 (Drosophila) (FZD4)	1.53	0.83	0.95	1.35	1.11	1.38	2.25	0.83
vMF	ILMN_1757186	GIMAP1	Homo sapiens GTPase, IMAP family member 1 (GIMAP1)	1.00	0.99	1.00	0.97	0.98	1.23	2.20	0.98
vMF	ILMN_2226223	GJB6	Homo sapiens gap junction protein, beta 6 (GJB6)	1.01	1.00	1.02	1.86	1.72	1.16	1.80	0.97
vMF	ILMN_2217513	GLB1L	Homo sapiens galactosidase, beta 1-like (GLB1L)	0.97	1.00	1.08	1.02	1.07	1.16	1.81	1.00
vMF	ILMN_2407389	GPNMB	Homo sapiens glycoprotein (transmembrane) nmb (GPNMB), transcrip	0.77	0.76	0.74	0.89	0.55	2.08	7.61	0.73
vMF	ILMN_2333766	HISPPD2A	Homo sapiens histidine acid phosphatase domain containing 2A (HISP	1.11	0.94	0.81	1.16	1.08	1.02	1.59	0.93
vMF	ILMN_1861270	HS.374023	Homo sapiens mRNA; cDNA DKFZp686N1644 (from clone DKFZp6	1.12	1.08	1.10	1.08	1.03	1.05	2.00	0.99
vMF	ILMN_1738237	HS1BP3	Homo sapiens HCLS1 binding protein 3 (HS1BP3)	1.00	0.99	0.99	0.97	1.02	1.01	1.54	1.02
vMF	ILMN_1696432	IDH1	Homo sapiens isocitrate dehydrogenase 1 (NADP+), soluble (IDH1)	1.22	0.96	1.01	1.00	1.09	1.51	6.04	1.07
vMF	ILMN_1714820	ITGB1	Homo sapiens integrin, beta 1 (fibronectin receptor, beta polypeptide, a	1.54	1.18	1.54	1.31	1.09	1.18	2.09	1.00
vMF	ILMN_1796755	ITGB5	Homo sapiens integrin, beta 5 (ITGB5) XM_944688 XM_944693	0.92	0.87	0.95	0.91	0.84	1.21	3.03	0.80
vMF	ILMN_2297765	KCNMA1	Homo sapiens potassium large conductance calcium-activated channel,	0.99	1.22	1.14	1.23	0.96	1.44	2.87	0.98
vMF	ILMN_1768814	KLHL6	Homo sapiens kelch-like 6 (Drosophila) (KLHL6)	0.99	1.04	1.23	1.18	1.08	1.19	1.97	1.12
vMF	ILMN_2326953	LAT2	Homo sapiens linker for activation of T cells family, member 2 (LAT2)	0.99	1.10	1.20	0.90	1.00	1.20	1.93	1.13
vMF	ILMN_1807556	LOC146177	PREDICTED: Homo sapiens hypothetical protein LOC146177 (LOC14	1.01	1.03	0.90	0.92	0.94	1.14	1.91	1.02
vMF	ILMN_1771286	LOC653513	PREDICTED: Homo sapiens similar to phosphodiesterase 4D interacti	1.10	1.16	1.81	1.45	1.22	1.31	1.96	0.96
vMF	ILMN_1675542	LOC729148	PREDICTED: Homo sapiens similar to lethal (2) k00619 CG4775-PA (	0.95	0.88	0.99	0.86	0.69	0.90	2.35	1.02
vMF	ILMN_1712719	MAP7	Homo sapiens microtubule-associated protein 7 (MAP7)	1.07	1.00	1.16	1.50	1.62	1.39	1.97	2.19
vMF	ILMN_2290118	MEGF9	Homo sapiens multiple EGF-like-domains 9 (MEGF9)	0.76	0.75	1.16	0.97	0.92	1.16	3.02	0.64
vMF	ILMN_1768035	MMP12	Homo sapiens matrix metallopeptidase 12 (macrophage elastase) (MM	1.03	1.03	1.40	0.92	0.97	1.45	2.80	1.00
vMF	ILMN_1795119	MYH3	Homo sapiens myosin, heavy chain 3, skeletal muscle, embryonic (MY	0.87	0.74	1.10	1.04	0.92	1.30	1.93	0.71
vMF	ILMN_1724907	NUDT3	Homo sapiens nudix (nucleoside diphosphate linked moiety X)-type m	0.86	0.72	0.98	0.75	0.89	0.92	1.88	1.39
vMF	ILMN_1672122	P4HTM	Homo sapiens prolyl 4-hydroxylase, transmembrane (endoplasmic retic	1.08	0.97	1.00	1.15	1.08	1.12	2.03	1.27
vMF	ILMN_1812552	PHCA	Homo sapiens phytoceramidase, alkaline (PHCA)	0.71	0.67	0.75	1.00	0.74	1.42	2.46	1.36
vMF	ILMN_1799860	PIGM	Homo sapiens phosphatidylinositol glycan anchor biosynthesis, class M	1.03	0.98	1.04	1.14	1.04	1.24	1.82	1.00
vMF	ILMN_1666924	PINK1	Homo sapiens PTEN induced putative kinase 1 (PINK1), nuclear gene	0.81	0.68	0.66	1.28	0.71	0.91	1.73	0.31
vMF	ILMN_1791569	PLXNA1	Homo sapiens plexin A1 (PLXNA1)	1.26	1.01	1.01	1.12	1.00	1.20	1.62	1.77
vMF	ILMN_1800164	PPFIA1	Homo sapiens protein tyrosine phosphatase, receptor type, f polypeptid	0.67	1.00	0.64	0.95	0.64	0.97	1.63	0.83
vMF	ILMN_2403889	PRMT5	Homo sapiens protein arginine methyltransferase 5 (PRMT5), transcrip	1.26	0.90	1.08	1.50	1.53	1.39	1.92	1.19
vMF	ILMN_1713829	PTGES	Homo sapiens prostaglandin E synthase (PTGES)	0.78	0.76	0.83	0.53	0.64	0.94	3.41	0.75
vMF	ILMN_1714393	RAB24	Homo sapiens RAB24, member RAS oncogene family (RAB24), trans	0.93	0.95	1.17	1.10	1.09	1.32	2.01	1.56
vMF	ILMN_1815264	RHBDD3	Homo sapiens rhomboid domain containing 3 (RHBDD3)	1.04	0.99	0.98	1.11	0.96	1.21	1.86	1.00
vMF	ILMN_2294976	RNASE4	Homo sapiens ribonuclease, RNase A family, 4 (RNASE4), transcript v	0.98	1.08	1.26	1.09	1.01	1.17	1.60	1.00
vMF	ILMN_1718960	SERPINB8	Homo sapiens serpin peptidase inhibitor, clade B (ovalbumin), member	0.80	0.79	0.82	1.22	0.83	1.11	1.54	1.07
vMF	ILMN_1767337	SFXN5	Homo sapiens sideroflexin 5 (SFXN5)	1.03	1.00	0.97	1.01	1.12	1.17	1.97	1.21
vMF	ILMN_1665538	SKP2	Homo sapiens S-phase kinase-associated protein 2 (p45) (SKP2), trans	0.69	0.76	1.01	0.91	1.04	0.83	1.58	0.91
vMF	ILMN_1807894	SLC7A8	Homo sapiens solute carrier family 7 (cationic amino acid transporter, y	1.13	0.92	0.97	0.85	0.84	1.41	3.26	0.93
vMF	ILMN_1709772	SNX5	Homo sapiens sorting nexin 5 (SNX5), transcript variant 2	1.03	1.25	1.38	1.01	0.95	1.36	1.94	0.82
vMF	ILMN_1699100	SOAT1	Homo sapiens sterol O-acyltransferase 1 (SOAT1), transcript variant 68	0.98	0.98	0.99	1.19	1.02	1.23	2.01	1.18
vMF	ILMN_2197577	SPRR2C	Homo sapiens small proline-rich protein 2C (pseudogene) (SPRR2C), r	1.06	0.97	1.07	2.00	2.03	1.21	1.75	0.84
vMF	ILMN_1699887	ST14	Homo sapiens suppression of tumorigenicity 14 (colon carcinoma, mat	0.98	0.97	0.93	1.39	1.17	1.29	2.13	1.08
vMF	ILMN_2216637	STK32B	Homo sapiens serine/threonine kinase 32B (STK32B)	1.05	1.03	1.12	1.38	0.96	1.19	1.53	1.00
vMF	ILMN_1791067	TESK1	Homo sapiens testis-specific kinase 1 (TESK1)	0.96	1.30	1.07	1.29	1.08	1.17	1.65	1.04
vMF	ILMN_1736527	TFCP2L1	Homo sapiens transcription factor CP2-like 1 (TFCP2L1)	1.01	1.00	1.02	1.25	1.11	1.18	1.60	0.94
vMF	ILMN_1764826	TFE3	Homo sapiens transcription factor binding to IGHM enhancer 3 (TFE3	0.88	1.00	1.05	0.98	0.80	0.90	1.65	0.81
vMF	ILMN_1808325	TM4SF19	PREDICTED: Homo sapiens transmembrane 4 L six family member 19	0.97	1.11	1.00	0.97	0.98	1.29	2.67	0.99
vMF	ILMN_1669931	TM9SF3	Homo sapiens transmembrane 9 superfamily member 3 (TM9SF3)	1.29	0.80	0.98	0.88	0.86	0.85	1.55	0.77
vMF	ILMN_1719649	TMEM63A	Homo sapiens transmembrane protein 63A (TMEM63A)	1.03	1.01	1.23	1.10	0.97	1.23	3.11	1.01
vMF	ILMN_1797021	TMEM86A	Homo sapiens transmembrane protein 86A (TMEM86A)	0.98	0.99	0.98	1.23	0.99	1.15	1.91	1.36
vMF	ILMN_1776325	UBE2Q1	Homo sapiens ubiquitin-conjugating enzyme E2Q (putative) 1 (UBE2Q	1.05	0.78	0.79	0.84	0.82	0.78	1.80	1.35
vMF	ILMN_1786139	VKORC1	Homo sapiens vitamin K epoxide reductase complex, subunit 1 (VKOR	1.20	0.95	1.01	1.01	1.12	1.14	1.86	0.98
vMF	ILMN_2075847	ZC3H10	Homo sapiens zinc finger CCCH-type containing 10 (ZC3H10)	1.31	0.83	0.92	1.40	1.20	1.04	1.87	0.38
vMF	ILMN_1677785	ZNF559	Homo sapiens zinc finger protein 559 (ZNF559)	1.39	0.96	0.87	1.19	1.07	1.13	1.73	0.89

mDC	ILMN_1687840	ABCB7	Homo sapiens ATP-binding cassette, sub-family B (MDR/TAP), memb	1.29	0.84	0.93	0.76	0.87	0.86	1.07	2.09
mDC	ILMN_1669633	ACP1	Homo sapiens acid phosphatase 1, soluble (ACP1), transcript variant 2	1.18	0.92	1.06	0.88	0.87	0.76	0.97	2.94
mDC	ILMN_1733690	AKAP7	Homo sapiens A kinase (PRKA) anchor protein 7 (AKAP7), transcript	1.25	1.00	1.00	1.52	1.17	1.11	1.37	3.90
mDC	ILMN_2311761	AP3S1	Homo sapiens adaptor-related protein complex 3, sigma 1 subunit (AP3	1.10	0.77	0.75	0.99	0.84	0.81	1.30	2.41
mDC	ILMN_1752340	ARF5	Homo sapiens ADP-ribosylation factor 5 (ARF5)	0.97	0.88	0.91	0.93	1.05	0.88	1.00	1.90
mDC	ILMN_1781010	ARHGEF3	Homo sapiens Rho guanine nucleotide exchange factor (GEF) 3 (ARH	1.08	0.86	0.58	0.80	0.69	0.80	1.10	6.73
mDC	ILMN_1803423	ARHGEF6	Homo sapiens Rac/Cdc42 guanine nucleotide exchange factor (GEF) 6	0.97	0.82	1.22	0.42	0.52	0.59	1.07	3.79
mDC	ILMN_1769566	ATG3	Homo sapiens ATG3 autophagy related 3 homolog (S. cerevisiae) (ATG	0.91	1.05	0.80	0.93	0.93	0.79	0.94	2.63
mDC	ILMN_1660577	ATP5G2	Homo sapiens ATP synthase, H+ transporting, mitochondrial F0 compl	1.11	1.07	1.03	0.89	0.89	0.88	0.93	2.08
mDC	ILMN_2351638	BEX4	Homo sapiens BEX family member 4 (BEX4)	1.37	0.86	0.73	0.77	0.82	0.84	1.26	1.76
mDC	ILMN_1695354	BMF	Homo sapiens Bcl2 modifying factor (BMF), transcript variant 2	0.98	1.07	0.78	0.78	0.92	0.93	0.96	2.96
mDC	ILMN_2308338	BMF	Homo sapiens Bcl2 modifying factor (BMF), transcript variant 4	1.06	1.11	0.86	0.83	0.93	1.05	0.94	3.02
mDC	ILMN_2072140	BTF3L4	Homo sapiens basic transcription factor 3-like 4 (BTF3L4)	0.87	1.43	1.24	0.75	0.86	0.88	1.24	2.61
mDC	ILMN_1652722	C14ORF2	Homo sapiens chromosome 14 open reading frame 2 (C14orf2)	1.09	0.87	0.86	0.88	0.80	0.93	1.14	3.71
mDC	ILMN_1680867	C6ORF61	PREDICTED: Homo sapiens chromosome 6 open reading frame 61 (C	1.26	0.88	0.88	0.86	0.91	0.94	1.08	2.30
mDC	ILMN_1672947	CAST	Homo sapiens calpastatin (CAST), transcript variant 11	1.43	0.95	0.99	1.04	1.02	1.02	1.02	2.36
mDC	ILMN_2322806	CAST	Homo sapiens calpastatin (CAST), transcript variant 8	1.44	0.98	0.82	0.68	0.77	0.63	0.94	3.78
mDC	ILMN_1654210	CD1C	Homo sapiens CD1c molecule (CD1C)	1.00	1.00	1.02	1.06	1.06	1.39	1.00	39.76
mDC	ILMN_1719433	CD1D	Homo sapiens CD1d molecule (CD1D)	1.01	1.01	1.01	1.00	0.99	1.08	1.11	5.24
mDC	ILMN_1702534	CD244	Homo sapiens CD244 molecule, natural killer cell receptor 2B4 (CD24	1.01	0.99	0.97	1.13	1.08	1.06	0.96	3.27
mDC	ILMN_1651316	CD69	Homo sapiens CD69 molecule (CD69)	1.02	1.03	1.00	1.00	1.00	1.00	1.00	4.22
mDC	ILMN_1786016	CHD1L	Homo sapiens chromodomain helicase DNA binding protein 1-like (CH	1.21	1.00	0.81	0.76	0.82	0.77	0.89	2.55
mDC	ILMN_1779401	CHP	Homo sapiens calcium binding protein P22 (CHP)	1.14	0.94	0.78	1.19	0.91	0.78	1.04	2.10
mDC	ILMN_1722390	CHRAC1	Homo sapiens chromatin accessibility complex 1 (CHRAC1)	0.98	1.13	1.03	1.23	1.07	1.03	1.29	5.78
mDC	ILMN_1777378	COMMD6	Homo sapiens COMM domain containing 6 (COMMD6), transcript va	1.08	1.00	0.98	1.03	1.08	1.02	1.03	1.92
mDC	ILMN_3237665	COX7A2L	Homo sapiens cytochrome c oxidase subunit VIIa polypeptide 2 like (C	1.11	1.02	0.88	1.00	0.79	0.77	0.94	1.71
mDC	ILMN_1706521	CSNK1G2	Homo sapiens casein kinase 1, gamma 2 (CSNK1G2)	1.05	0.95	0.89	1.05	1.01	0.95	0.85	1.73
mDC	ILMN_2337928	CXCR5	Homo sapiens chemokine (C-X-C motif) receptor 5 (CXCR5), transcri	1.02	0.99	1.05	1.18	1.11	1.22	1.22	2.12
mDC	ILMN_1775508	CYLD	Homo sapiens cylindromatosis (turban tumor syndrome) (CYLD)	0.79	1.55	1.01	1.08	0.83	0.78	0.79	3.41
mDC	ILMN_1746257	DAZAP1	Homo sapiens DAZ associated protein 1 (DAZAP1), transcript variant	1.23	1.09	0.75	1.01	0.86	0.85	0.85	1.55
mDC	ILMN_1718988	DAZAP2	Homo sapiens DAZ associated protein 2 (DAZAP2)	1.02	0.92	1.07	0.83	0.71	0.73	1.16	1.58
mDC	ILMN_1802456	DCTD	Homo sapiens dCMP deaminase (DCTD), transcript variant 2	0.93	0.98	0.82	0.81	0.71	0.71	1.38	1.70
mDC	ILMN_1744059	DCTN6	Homo sapiens dynactin 6 (DCTN6)	1.02	1.01	0.69	0.98	0.80	0.85	0.98	1.76
mDC	ILMN_1735461	DDX21	Homo sapiens DEAD (Asp-Glu-Ala-Asp) box polypeptide 21 (DDX21	1.45	1.14	0.71	0.78	0.68	0.71	0.98	6.37
mDC	ILMN_1791896	EBAG9	Homo sapiens estrogen receptor binding site associated, antigen, 9 (EB	1.21	0.78	0.80	0.84	0.80	0.75	1.14	2.66
mDC	ILMN_1665717	EIF2S3	Homo sapiens eukaryotic translation initiation factor 2, subunit 3 gamn	1.21	0.98	0.84	0.87	0.63	0.69	1.14	2.89
mDC	ILMN_2192693	EIF3M	Homo sapiens eukaryotic translation initiation factor 3, subunit M (EIF	1.16	0.94	0.77	1.07	1.00	0.87	0.86	2.63
mDC	ILMN_2174369	ELOVL5	Homo sapiens ELOVL family member 5, elongation of long chain fatty	1.63	1.24	1.05	0.92	0.98	0.91	1.03	8.80
mDC	ILMN_1713835	ENHO	Homo sapiens energy homeostasis associated (ENHO)	1.05	1.03	0.97	1.18	1.08	0.97	0.95	3.77
mDC	ILMN_2323048	ERP29	Homo sapiens endoplasmic reticulum protein 29 (ERP29), transcript va	0.94	0.85	0.74	1.00	0.87	1.10	1.18	2.33
mDC	ILMN_1786789	FAM102B	Homo sapiens family with sequence similarity 102, member B (FAM10	1.53	1.01	1.33	0.75	0.94	0.69	1.15	3.38
mDC	ILMN_1778611	GBAS	Homo sapiens glioblastoma amplified sequence (GBAS)	1.50	0.81	0.84	0.85	0.82	0.89	1.44	3.65
mDC	ILMN_2384056	GPER	Homo sapiens G protein-coupled estrogen receptor 1 (GPER), transcrip	1.17	1.46	1.06	1.11	1.07	1.09	1.10	3.08
mDC	ILMN_1684227	GPR146	Homo sapiens G protein-coupled receptor 146 (GPR146)	0.97	1.11	1.07	1.12	1.11	1.04	1.04	1.69
mDC	ILMN_2335718	HNRNPAB	Homo sapiens heterogeneous nuclear ribonucleoprotein A/B (HNRNPA	1.00	1.05	0.77	1.19	0.77	0.96	1.12	1.65
mDC	ILMN_1739582	HOXA9	Homo sapiens homeobox A9 (HOXA9)	1.01	1.07	1.17	1.15	1.19	1.13	1.08	3.13
mDC	ILMN_1881960	HS.444785	full-length cDNA clone CS0DF032YA11 of Fetal brain of Homo sapien	1.00	1.00	1.00	1.00	1.03	1.00	1.06	3.88
mDC	ILMN_1846922	HS.568741	TC125227 Human breast cancer tissue, large insert, pCMV expression	1.08	0.94	0.91	1.22	1.17	1.23	1.04	2.58
mDC	ILMN_1793474	INSIG1	Homo sapiens insulin induced gene 1 (INSIG1), transcript variant 2	0.81	1.19	1.58	0.93	0.97	1.02	0.95	3.95
mDC	ILMN_3251605	KLHL28	Homo sapiens kelch-like 28 (Drosophila) (KLHL28)	0.75	1.08	1.06	0.77	1.11	0.86	1.06	2.65
mDC	ILMN_1728132	LDHB	Homo sapiens lactate dehydrogenase B (LDHB)	0.87	0.84	0.80	0.84	0.98	0.99	1.04	4.14
mDC	ILMN_1687306	LGALS2	Homo sapiens lectin, galactoside-binding, soluble, 2 (LGALS2)	0.99	1.00	1.00	1.12	1.24	1.17	1.04	4.75
mDC	ILMN_3266666	LOC100128196	PREDICTED: Homo sapiens misc_RNA (LOC100128196), miscRNA.	1.50	0.96	0.87	1.23	1.07	0.83	0.83	3.17
mDC	ILMN_3256674	LOC100128485	PREDICTED: Homo sapiens similar to conserved hypothetical protein	1.23	1.00	1.11	1.11	1.07	1.00	1.00	2.11
mDC	ILMN_3177271	LOC100129585	PREDICTED: Homo sapiens similar to hCG2011544 (LOC100129585	1.49	0.83	0.84	1.05	0.79	0.78	1.04	2.32
mDC	ILMN_3240986	LOC100134584	PREDICTED: Homo sapiens hypothetical protein LOC100134584 (LO	0.94	1.19	1.03	0.91	1.17	1.22	1.05	2.64
mDC	ILMN_3278506	LOC148430	PREDICTED: Homo sapiens misc_RNA (LOC148430), miscRNA.	1.02	1.11	0.81	1.00	1.14	0.97	0.89	1.73
mDC	ILMN_3199489	LOC282997	PREDICTED: Homo sapiens misc_RNA (LOC282997), miscRNA.	0.99	1.06	1.02	1.08	1.07	1.08	1.11	1.74
mDC	ILMN_1695034	LOC642817	PREDICTED: Homo sapiens hypothetical LOC642817 (LOC642817)	1.13	0.93	0.97	0.94	0.94	0.95	0.89	1.99
mDC	ILMN_1669424	LOC646531	PREDICTED: Homo sapiens similar to nuclease sensitive element bind	0.86	0.93	0.82	1.02	0.87	0.92	1.15	2.04
mDC	ILMN_1725170	LOC650557	PREDICTED: Homo sapiens similar to HLA class II histocompatibility	1.37	1.91	1.37	0.62	0.86	0.74	0.91	3.92
mDC	ILMN_1685378	LOC728973	PREDICTED: Homo sapiens similar to 40S ribosomal protein S7 (S8)	1.21	1.01	1.07	0.91	0.96	0.90	0.85	1.79
mDC	ILMN_3229467	LOC729217	PREDICTED: Homo sapiens misc_RNA (LOC729217), miscRNA.	1.10	1.01	1.02	1.27	1.07	1.19	1.06	3.62
mDC	ILMN_3227529	LOC729236	PREDICTED: Homo sapiens misc_RNA (LOC729236), miscRNA.	1.11	1.12	1.00	1.15	1.20	0.99	0.99	1.91
mDC	ILMN_1665943	MAP4K1	Homo sapiens mitogen-activated protein kinase kinase kinase kinase 1	1.03	1.02	1.01	1.01	0.83	1.10	1.09	5.79
mDC	ILMN_2365111	MAP4K1	Homo sapiens mitogen-activated protein kinase kinase kinase kinase 1	1.02	1.01	1.02	1.02	0.87	1.09	1.06	5.50
mDC	ILMN_1786189	MKI67IP	Homo sapiens MKI67 (FHA domain) interacting nucleolar phosphopro	1.05	0.66	0.86	0.81	0.67	0.72	0.96	2.50
mDC	ILMN_2148796	MND1	Homo sapiens meiotic nuclear divisions 1 homolog (S. cerevisiae) (MN	1.00	1.00	1.00	1.00	1.00	1.00	1.00	2.58
mDC	ILMN_1676159	MST4	Homo sapiens serine/threonine protein kinase MST4 (MST4), transcrip	1.03	1.51	1.46	0.75	0.87	0.74	0.90	4.39
mDC	ILMN_1737738	NDUFA12	Homo sapiens NADH dehydrogenase (ubiquinone) 1 alpha subcomplex	1.29	0.71	0.70	0.90	0.89	1.03	1.21	2.54
mDC	ILMN_1807397	NDUFB5	Homo sapiens NADH dehydrogenase (ubiquinone) 1 beta subcomplex,	1.10	0.82	0.77	0.95	0.86	0.78	1.01	1.70
mDC	ILMN_1720344	NIPA2	Homo sapiens non imprinted in Prader-Willi/Angelman syndrome 2 (N	0.91	1.03	0.83	0.90	0.76	0.90	1.21	2.57
mDC	ILMN_2352121	NT5C3	Homo sapiens 5'-nucleotidase, cytosolic III (NT5C3), transcript variant	1.01	1.29	1.16	0.85	0.85	0.85	0.90	5.87
mDC	ILMN_1784467	NUP210	Homo sapiens nucleoporin 210kDa (NUP210)	0.98	1.02	0.94	1.14	1.14	1.05	1.10	3.05
mDC	ILMN_1771835	NUP54	Homo sapiens nucleoporin 54kDa (NUP54)	1.21	1.22	0.99	1.02	1.02	1.01	1.01	2.32
mDC	ILMN_1731851	OXA1L	Homo sapiens oxidase (cytochrome c) assembly 1-like (OXA1L)	1.06	0.97	0.89	0.99	0.95	0.91	1.03	3.21
mDC	ILMN_1710571	PAPD5	Homo sapiens PAP associated domain containing 5 (PAPD5), transcrip	0.57	0.96	0.78	1.39	1.27	1.22	1.13	4.63
mDC	ILMN_1780132	PELI2	Homo sapiens pellino homolog 2 (Drosophila) (PELI2)	0.85	0.90	1.09	1.14	1.13	1.17	1.36	1.93
mDC	ILMN_1733666	PLDN	Homo sapiens pallidin homolog (mouse) (PLDN)	1.27	1.12	1.03	0.99	0.98	0.96	0.97	2.18
mDC	ILMN_1767894	POLB	Homo sapiens polymerase (DNA directed), beta (POLB)	0.89	0.89	0.83	1.06	0.90	0.71	1.12	3.38
mDC	ILMN_1769091	PRCP	Homo sapiens prolylcarboxypeptidase (angiotensinase C) (PRCP), tran	1.06	0.86	0.76	0.95	0.74	0.84	1.09	2.55
mDC	ILMN_2367215	PRCP	Homo sapiens prolylcarboxypeptidase (angiotensinase C) (PRCP), tran	1.24	0.90	0.74	0.92	0.86	0.82	1.15	2.81
mDC	ILMN_1741244	PRR8	Homo sapiens proline rich 8 (PRR8)	1.05	1.00	1.21	1.12	1.04	0.91	1.23	2.84
mDC	ILMN_1795927	PSPC1	Homo sapiens paraspeckle component 1 (PSPC1), transcript variant alp	1.12	1.19	0.99	1.14	0.90	0.90	0.96	2.89
mDC	ILMN_1781454	RAE1	Homo sapiens RAE1 RNA export 1 homolog (S. pombe) (RAE1), trans	0.87	1.22	0.99	0.75	0.71	0.88	0.95	1.60
mDC	ILMN_1745282	RAGE	Homo sapiens renal tumor antigen (RAGE)	1.25	0.93	1.01	1.63	1.13	1.21	1.22	2.30
mDC	ILMN_2375319	RASGRP2	Homo sapiens RAS guanyl releasing protein 2 (calcium and DAG-regu	1.17	0.95	0.84	0.97	1.03	1.00	0.91	2.14
mDC	ILMN_1770053	RBBP7	Homo sapiens retinoblastoma binding protein 7 (RBBP7)	1.33	1.20	0.80	0.96	0.89	0.90	0.88	2.33
mDC	ILMN_1720124	RCC2	Homo sapiens regulator of chromosome condensation 2 (RCC2)	0.96	1.06	0.72	0.93	0.83	0.88	1.08	4.19
mDC	ILMN_1751886	REC8	Homo sapiens REC8 homolog (yeast) (REC8), transcript variant 1	1.00	0.96	0.97	0.95	0.93	1.04	1.25	4.20
mDC	ILMN_1748614	RELT	Homo sapiens RELT tumor necrosis factor receptor (RELT), transcript	0.82	1.41	1.31	0.71	0.78	0.80	1.12	3.33
mDC	ILMN_2197365	RGS2	Homo sapiens regulator of G-protein signalling 2, 24kDa (RGS2)	1.13	1.18	0.97	0.97	0.89	0.90	0.79	3.02
mDC	ILMN_2079386	RPL22	Homo sapiens ribosomal protein L22 (RPL22)	1.09	1.14	0.97	0.75	0.81	0.60	0.77	5.48
mDC	ILMN_1684306	S100A4	Homo sapiens S100 calcium binding protein A4 (S100A4), transcript v	1.08	1.21	1.10	1.03	1.04	1.09	1.07	2.70
mDC	ILMN_1746598	SCNM1	Homo sapiens sodium channel modifier 1 (SCNM1)	1.16	0.99	1.00	1.12	1.11	0.99	1.08	1.78
mDC	ILMN_1724422	SELL	Homo sapiens selectin L (SELL)	1.00	1.00	1.00	1.00	1.02	1.04	1.00	25.44
mDC	ILMN_2373266	SFRS12	Homo sapiens splicing factor, arginine/serine-rich 12 (SFRS12), transc	1.20	1.08	0.93	0.83	0.85	0.95	1.05	3.16
mDC	ILMN_3235647	SIK1	Homo sapiens salt-inducible kinase 1 (SIK1)	0.68	1.60	1.29	1.16	1.07	1.01	1.06	3.10

mDC	ILMN_1653429	SLC35A3	Homo sapiens solute carrier family 35 (UDP-N-acetylglucosamine (UD	1.32	1.06	1.18	1.23	1.07	1.07	1.13	3.18
mDC	ILMN_1720373	SLC7A5	Homo sapiens solute carrier family 7 (cationic amino acid transporter, y	0.77	1.14	0.94	1.39	1.00	1.03	1.04	4.67
mDC	ILMN_1706553	SMG7	Homo sapiens Smg-7 homolog, nonsense mediated mRNA decay facto	0.79	1.05	0.91	1.04	0.92	0.97	1.12	3.22
mDC	ILMN_1788251	SNN	Homo sapiens stannin (SNN)	1.08	0.99	0.67	1.06	1.09	1.06	0.84	1.63
mDC	ILMN_1799381	SNORD14A	Homo sapiens small nucleolar RNA, C/D box 14A (SNORD14A), sma	0.91	1.34	1.37	0.89	0.77	0.83	0.86	2.30
mDC	ILMN_2400644	SRGAP3	Homo sapiens SLIT-ROBO Rho GTPase activating protein 3 (SRGAP3	1.35	0.99	1.15	1.41	1.25	1.21	1.25	4.81
mDC	ILMN_1660186	SYF2	Homo sapiens SYF2 homolog, RNA splicing factor (S. cerevisiae) (SY	0.99	0.91	0.91	1.01	0.98	0.94	0.93	1.81
mDC	ILMN_1676408	TAGAP	Homo sapiens T-cell activation RhoGTPase activating protein (TAGAP	0.98	1.02	1.01	1.11	1.12	1.17	1.12	2.92
mDC	ILMN_2333774	TAGAP	Homo sapiens T-cell activation RhoGTPase activating protein (TAGAP	0.94	0.98	1.07	1.15	1.15	1.18	1.21	3.55
mDC	ILMN_1742450	TAPBP	Homo sapiens TAP binding protein (tapasin) (TAPBP), transcript varian	1.03	1.00	0.78	1.01	1.12	0.98	0.96	2.02
mDC	ILMN_1787511	THUMPD2	Homo sapiens THUMP domain containing 2 (THUMPD2)	1.13	0.98	0.79	0.95	0.86	0.94	1.03	4.28
mDC	ILMN_2092756	TMEM109	Homo sapiens transmembrane protein 109 (TMEM109)	1.22	0.97	0.63	1.04	0.70	0.78	0.82	1.78
mDC	ILMN_1714623	TOMM22	Homo sapiens translocase of outer mitochondrial membrane 22 homolo	1.25	0.61	0.67	0.87	0.87	0.80	0.78	2.91
mDC	ILMN_2065606	TOMM40L	Homo sapiens translocase of outer mitochondrial membrane 40 homolo	0.96	0.99	1.17	1.05	0.98	1.09	1.27	1.74
mDC	ILMN_2192316	TOP1	Homo sapiens topoisomerase (DNA) I (TOP1)	0.88	1.03	0.96	0.76	0.86	0.82	0.93	2.21
mDC	ILMN_2293374	TOP1MT	Homo sapiens topoisomerase (DNA) I, mitochondrial (TOP1MT), nucl	1.00	1.00	1.00	1.02	1.00	1.06	1.01	2.56
mDC	ILMN_1684929	TOPBP1	Homo sapiens topoisomerase (DNA) II binding protein 1 (TOPBP1)	1.48	0.79	0.69	0.81	0.81	0.78	1.06	1.70
mDC	ILMN_2351930	TRIM33	Homo sapiens tripartite motif-containing 33 (TRIM33), transcript varia	0.97	1.44	0.58	0.72	0.64	0.70	1.01	1.79
mDC	ILMN_1718621	TSPAN32	Homo sapiens tetraspanin 32 (TSPAN32), transcript variant 2	0.99	0.99	0.99	1.04	1.05	1.06	1.03	4.08
mDC	ILMN_1810729	UBL3	Homo sapiens ubiquitin-like 3 (UBL3)	0.61	1.27	0.80	0.75	0.53	0.62	0.87	1.86
mDC	ILMN_1799601	UHRF1BP1L	Homo sapiens UHRF1 binding protein 1-like (UHRF1BP1L), transcrip	1.20	1.19	1.18	0.92	0.88	0.85	1.09	6.77
mDC	ILMN_1718853	UQCRC2	Homo sapiens ubiquinol-cytochrome c reductase core protein II (UQCI	1.20	0.83	0.80	0.88	0.84	0.86	1.02	1.86
mDC	ILMN_1659703	WWP2	Homo sapiens WW domain containing E3 ubiquitin protein ligase 2 (W	0.59	1.31	0.93	0.73	0.88	0.62	1.19	3.55
