# Supplementary material for: Transcriptional fingerprints of antigen-presenting cell subsets in the human vaginal mucosa and skin reflect tissue-specific immune microenvironments
Source: Genome Med. 2014 Nov 25;6(11):98. doi: 10.1186/s13073-014-0098-y (PMC4268898; doi:10.1186/s13073-014-0098-y)
Supplement: Additional file 6: Figure S3. — Pathway enrichment scores in vaginal DC subsets. [file 13073_2014_98_MOESM6_ESM.pdf]

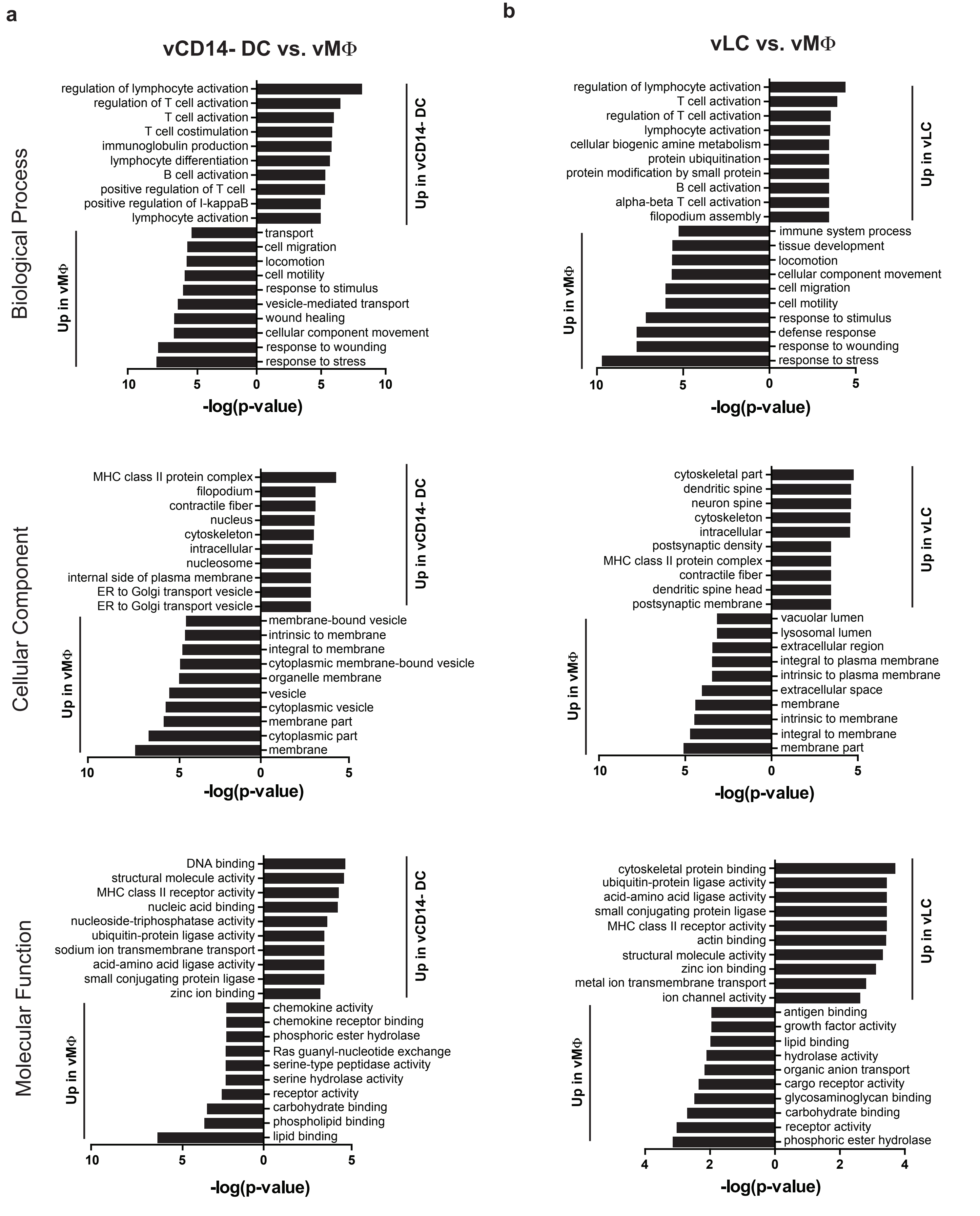

Figure S3: GO enrichment analysis for transcripts significantly differently expressed between vCD14- DC and vMΦ (a) or vLC and vMΦ (b). Biological process, cellular component and molecular function classifications are represented separately.
