# Supplementary material for: Transcriptional fingerprints of antigen-presenting cell subsets in the human vaginal mucosa and skin reflect tissue-specific immune microenvironments
Source: Genome Med. 2014 Nov 25;6(11):98. doi: 10.1186/s13073-014-0098-y (PMC4268898; doi:10.1186/s13073-014-0098-y)
Supplement: Additional file 8: Figure S5. — Transcripts specific for skin APC populations. [file 13073_2014_98_MOESM8_ESM.pdf]

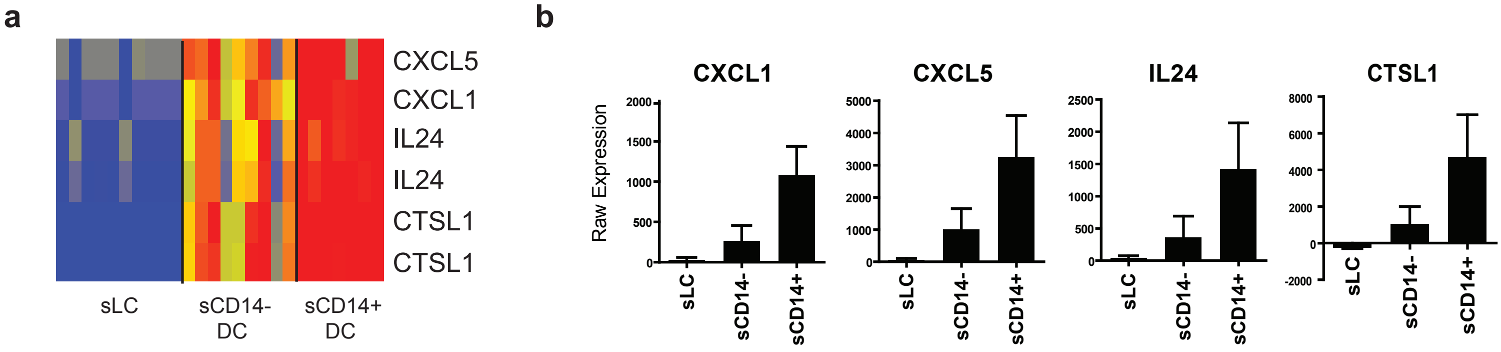

Figure S5: 6 transcripts representing 4 genes differentially expressed between the 3 skin DC subsets. a. Hierarchical clustering of the 6 transcripts differentially expressed in the 3 skin DC subsets. **b.** Bar charts representing the mean raw expression value for 4 of the 6 transcripts. Bars represent the standard deviation.
