# Supplementary material for: Transcriptional fingerprints of antigen-presenting cell subsets in the human vaginal mucosa and skin reflect tissue-specific immune microenvironments
Source: Genome Med. 2014 Nov 25;6(11):98. doi: 10.1186/s13073-014-0098-y (PMC4268898; doi:10.1186/s13073-014-0098-y)
Supplement: Additional file 9: Figure S6. — Results of comparisons between sLCs and sCD14+ DCs. [file 13073_2014_98_MOESM9_ESM.pdf]

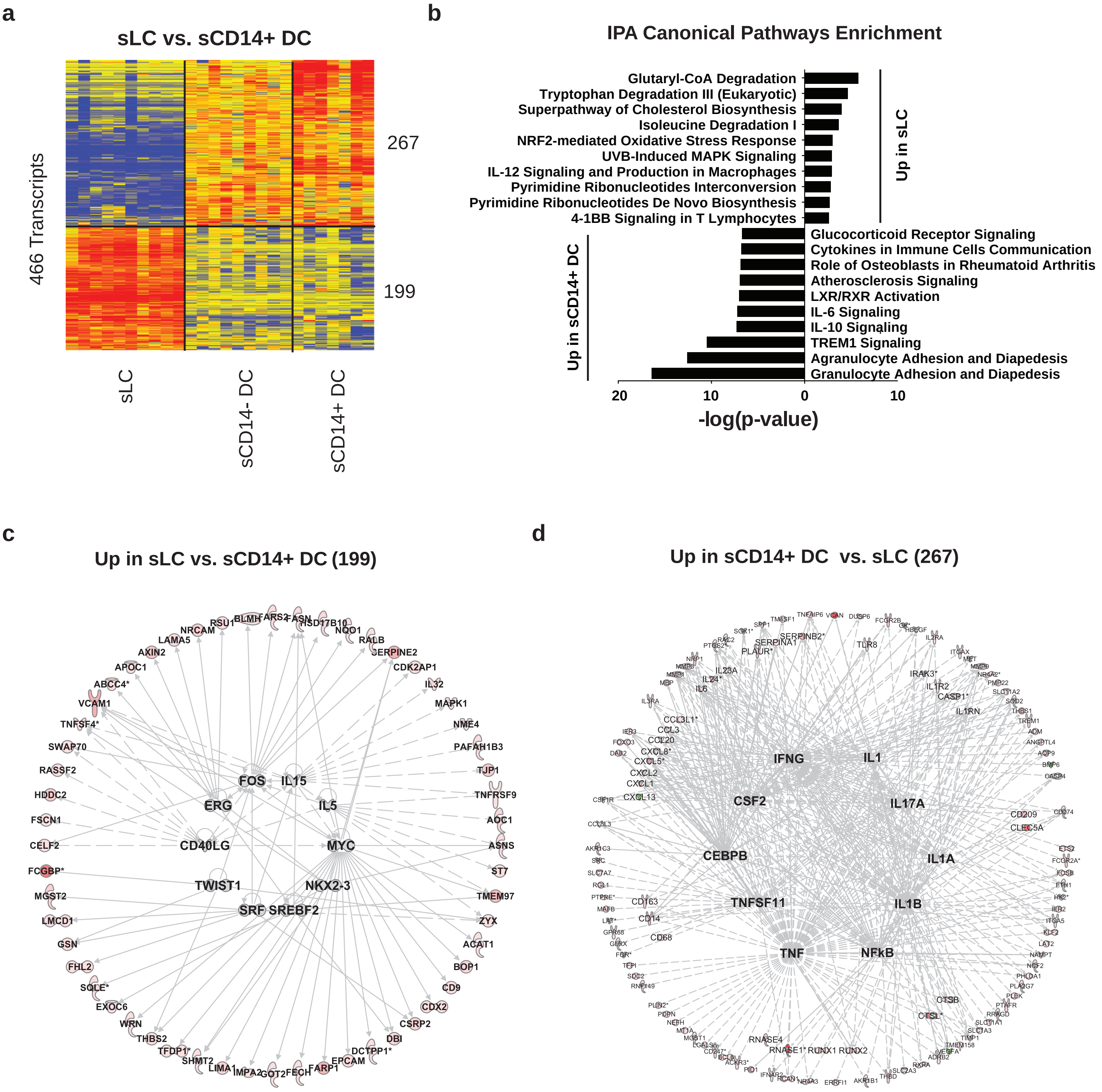

Figure S6: Transcriptional analysis of sLC vs. sCD14+ DC. **a.** Hierarchical clustering of the 466 transcripts (identified by post-hoc Tukey test) differentially expressed between sLC and sCD14+ DC. **b.** Bar chart representing the IPA canonical pathway enrichment for the 199 transcripts over-expressed in sLC and the 267 transcripts over-expressed sCD14+ DC. **c.** Network analysis of the 199 transcripts over-expressed in sLC. **d.** Network analysis of the 267 transcripts over-expressed in sCD14+ DC.
