# Supplementary material for: Transcriptional fingerprints of antigen-presenting cell subsets in the human vaginal mucosa and skin reflect tissue-specific immune microenvironments
Source: Genome Med. 2014 Nov 25;6(11):98. doi: 10.1186/s13073-014-0098-y (PMC4268898; doi:10.1186/s13073-014-0098-y)
Supplement: Additional file 10: Figure S7. — Pathways enrichment scores in skin LCs and skin CD14- DCs. [file 13073_2014_98_MOESM10_ESM.pdf]

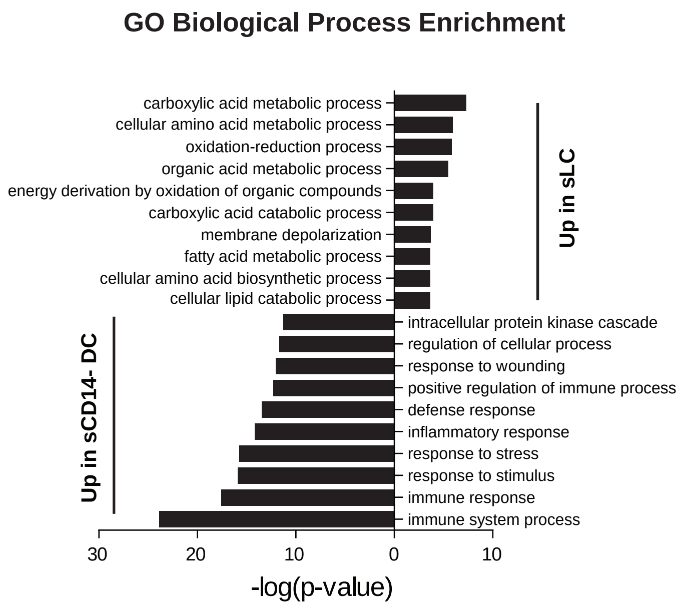

Figure S7: Gene ontology biological process enrichment in sLC and sCD14$^-$ DC.
