# Supplementary material for: Transcriptional fingerprints of antigen-presenting cell subsets in the human vaginal mucosa and skin reflect tissue-specific immune microenvironments
Source: Genome Med. 2014 Nov 25;6(11):98. doi: 10.1186/s13073-014-0098-y (PMC4268898; doi:10.1186/s13073-014-0098-y)
Supplement: Additional file 11: Figure S8. — DETs between skin and vaginal APC subsets. [file 13073_2014_98_MOESM11_ESM.pdf]

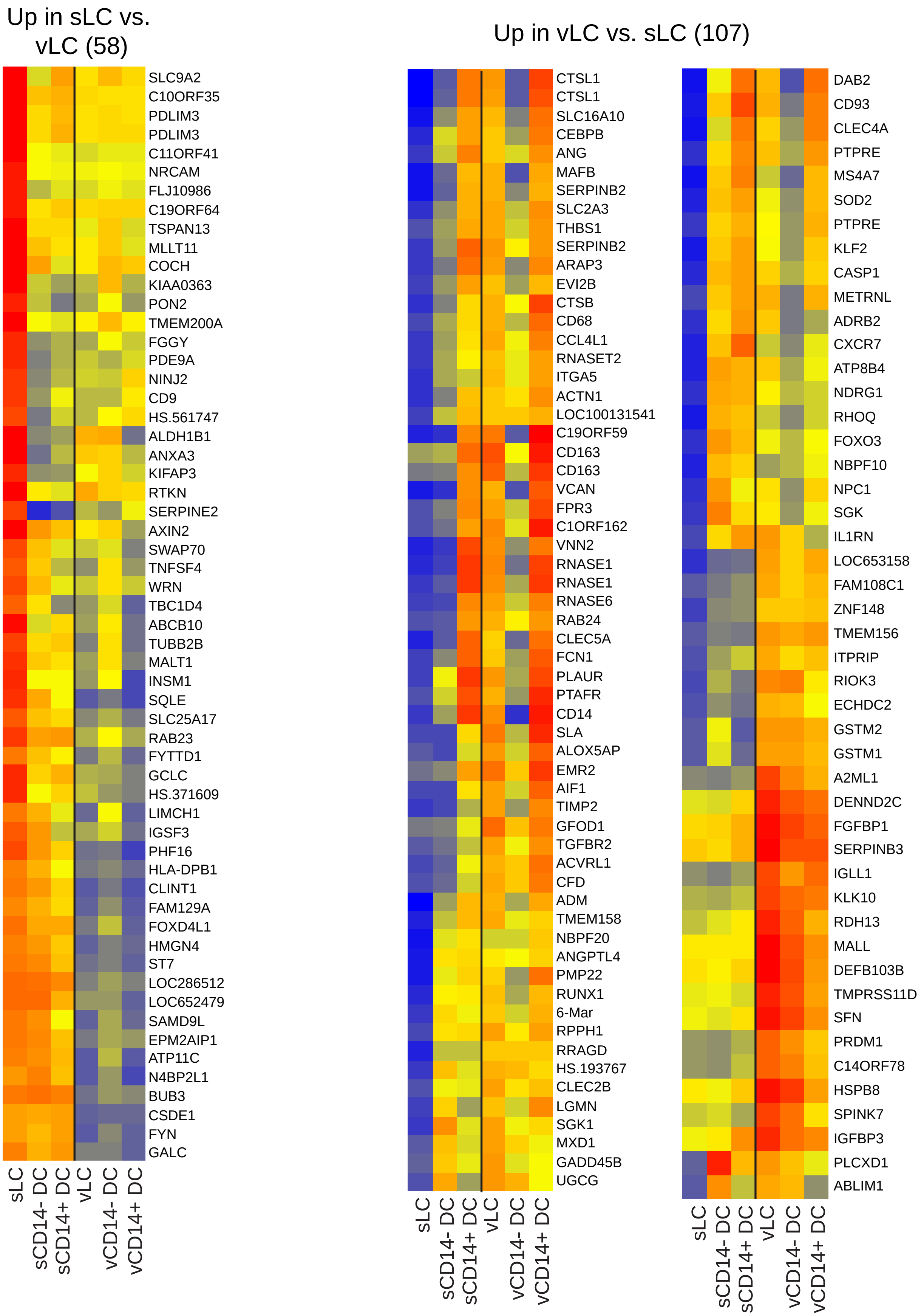

Figure S8 - Hierarchical clustering of the 165 transcripts differentially expressed between sLC and vLC.
Left panel: 58 transcriptsover-expressed in sLC. Right panel: 107 transcripts over-expressed in vLC. Data are normalized to the median of all samples.
