# Supplementary material for: Transcriptional fingerprints of antigen-presenting cell subsets in the human vaginal mucosa and skin reflect tissue-specific immune microenvironments
Source: Genome Med. 2014 Nov 25;6(11):98. doi: 10.1186/s13073-014-0098-y (PMC4268898; doi:10.1186/s13073-014-0098-y)
Supplement: Additional file 13: Figure S10. — DETs between skin and vaginal APC subsets. [file 13073_2014_98_MOESM13_ESM.pdf]

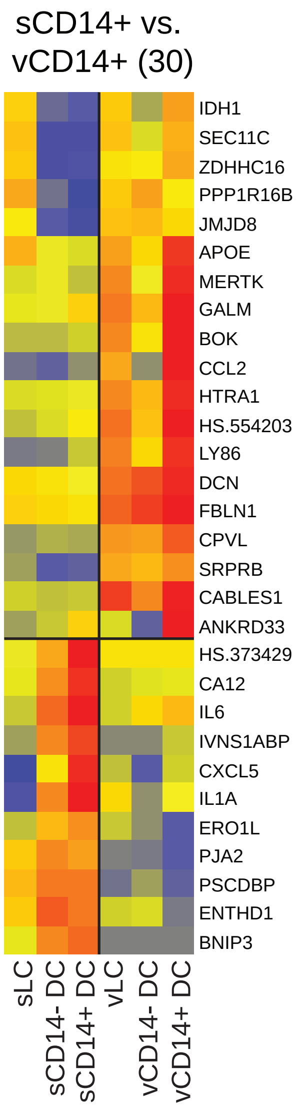

Figure S10 - Hierarchical clustering of the 30 transcripts differentially expressed between sCD14+ and vCD14+ DC. Data are normalized to the median of all samples.
