# Supplementary material for: Transcriptional fingerprints of antigen-presenting cell subsets in the human vaginal mucosa and skin reflect tissue-specific immune microenvironments
Source: Genome Med. 2014 Nov 25;6(11):98. doi: 10.1186/s13073-014-0098-y (PMC4268898; doi:10.1186/s13073-014-0098-y)
Supplement: Additional file 15: Figure S12. — Raw transcriptional expression of C-type lectins, TLRs, chemokines, cytokines and their receptors in all APC subsets. [file 13073_2014_98_MOESM15_ESM.pdf]

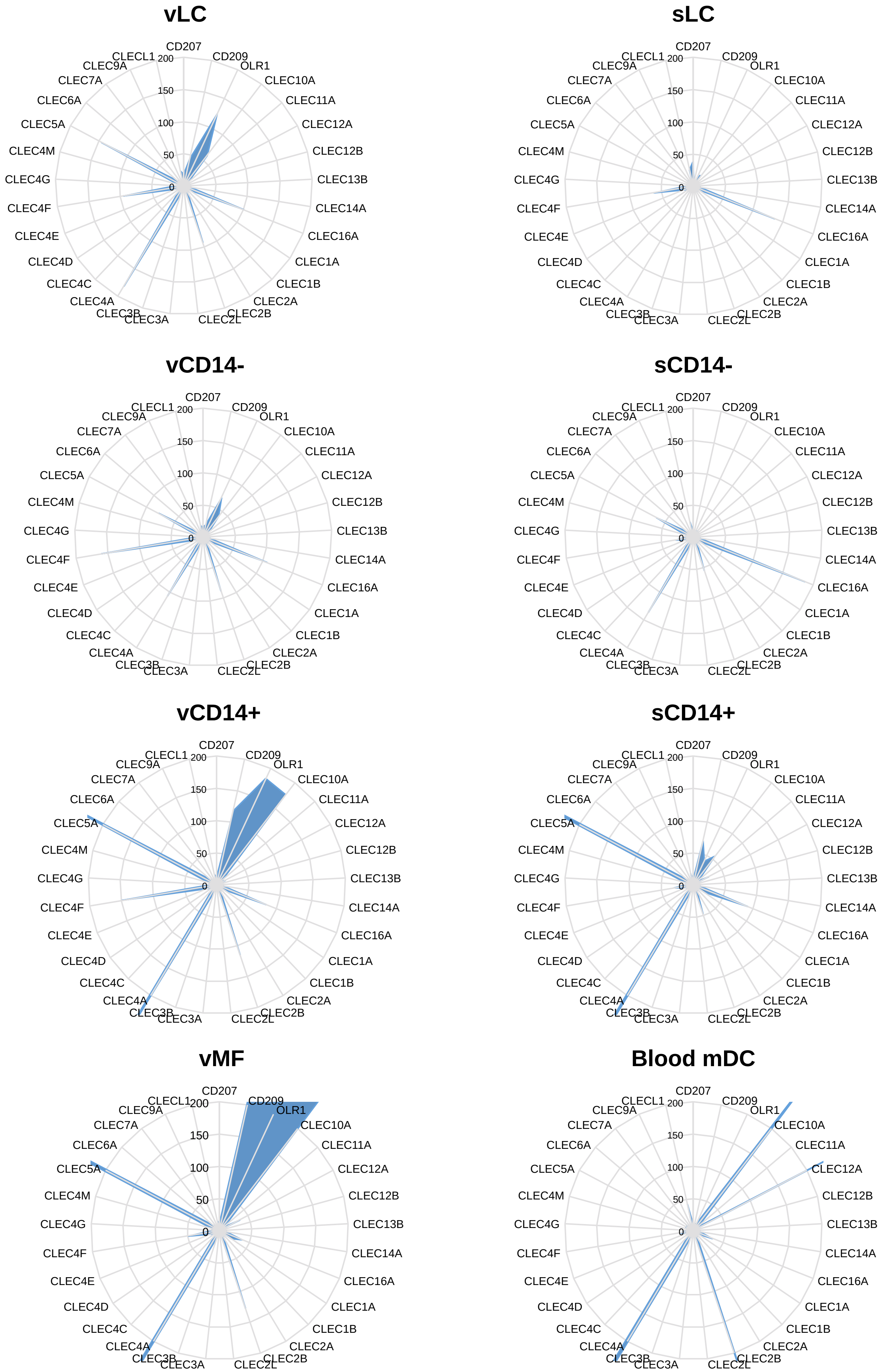

Figure S12: Radar charts representing the average C-type lectin raw expression values for vaginal, skin and blood APC subsets
