# Supplementary material for: Transcriptional fingerprints of antigen-presenting cell subsets in the human vaginal mucosa and skin reflect tissue-specific immune microenvironments
Source: Genome Med. 2014 Nov 25;6(11):98. doi: 10.1186/s13073-014-0098-y (PMC4268898; doi:10.1186/s13073-014-0098-y)
Supplement: Additional file 16: Table S2. — Expression of surface molecules, cytokines and chemokines on all APC subsets. [file 13073_2014_98_MOESM16_ESM.pdf]

Illumina ID	Symbol	Definition	Skin			Vagina				Blood
			sLC	sCD14- DC	sCD14+ DC	vLC	vCD14- DC	vCD14+ DC	vMF	mDC
ILMN_1769013	ASGR1	Homo sapiens asialoglycoprotein receptor 1 (ASGR1)	+	+	+	+	+	++	++	+
ILMN_2342638	ASGR2	Homo sapiens asialoglycoprotein receptor 2 (ASGR2)	+	+	+	+	+	+	+	+
ILMN_1700340	ASGR2	Homo sapiens asialoglycoprotein receptor 2 (ASGR2)	+	+	+	-	-	+	+	+
ILMN_1694966	ASGR2	Homo sapiens asialoglycoprotein receptor 2 (ASGR2)	+	+	+	+	+	+	+	+
ILMN_1723520	CD1A	Homo sapiens CD1a molecule (CD1A)	-	-	-	+	+	+	+	+
ILMN_1718754	CD207	Homo sapiens CD207 molecule, langerin (CD207)	+	-	-	+	+	+	+	+
ILMN_1676372	CD209	Homo sapiens CD209 molecule (CD209)	-	-	+	+	+	++	++	-
ILMN_2070940	CD302	Homo sapiens CD302 molecule (CD302)	+	+	+	+	+	+	+	+
ILMN_2367818	CD40	Homo sapiens CD40 molecule, TNF receptor superfamily me	+	+	+	+	+	+	+	+
ILMN_2188333	CD69	Homo sapiens CD69 molecule (CD69)	+	+	+	+	+	-	+	+++
ILMN_1716736	CD80	Homo sapiens CD80 molecule (CD80)	+	+	+	+	+	+	+	-
ILMN_2328666	CD83	Homo sapiens CD83 molecule (CD83)	+++	+++	+++	+++	+++	+++	+++	+++
ILMN_1780582	CD83	Homo sapiens CD83 molecule (CD83)	+++	+++	+++	+++	+++	+++	+++	+++
ILMN_1782560	CD86	Homo sapiens CD86 antigen (CD28 antigen ligand 2, B7-2 a	+	+	+	+	+	+	+	+
ILMN_1714602	CD86	Homo sapiens CD86 molecule (CD86)	+++	+++	+++	+++	+++	+++	+++	+++
ILMN_1691339	CLEC1A	Homo sapiens C-type lectin domain family 1, member A (CL	-	+	+	+	+	+	+	+
ILMN_1745103	CLEC1B	Homo sapiens C-type lectin domain family 1, member B (CL	ND	ND	ND	ND	ND	ND	ND	ND
ILMN_1779144	CLEC2A	Homo sapiens C-type lectin domain family 2, member A (CL	-	-	-	+	+	+	+	+
ILMN_1784608	CLEC2B	Homo sapiens C-type lectin domain family 2, member B (CL	-	+	+	+	+	++	++	++
ILMN_1791749	CLEC2L	PREDICTED: Homo sapiens C-type lectin domain family 2,	ND	ND	ND	ND	ND	ND	ND	ND
ILMN_2220735	CLEC3A	Homo sapiens C-type lectin domain family 3, member A (CL	ND	ND	ND	ND	ND	ND	ND	ND
ILMN_1682176	CLEC3B	Homo sapiens C-type lectin domain family 3, member B (CL	-	-	-	+	+	+	+	-
ILMN_2399363	CLEC4A	Homo sapiens C-type lectin domain family 4, member A (CL	-	++	+++	++	++	++	+++	+++
ILMN_1709204	CLEC4A	Homo sapiens C-type lectin domain family 4, member A (CL	+	+	+	+	+	+	+	+
ILMN_1682259	CLEC4C	Homo sapiens C-type lectin domain family 4, member C (CL	-	-	-	-	-	-	-	+
ILMN_1808979	CLEC4D	Homo sapiens C-type lectin domain family 4, member D (CL	ND	ND	ND	ND	ND	ND	ND	ND
ILMN_1771664	CLEC4E	Homo sapiens C-type lectin domain family 4, member E (CL	+	+	+	+	+	+	+	+
ILMN_1723115	CLEC4F	Homo sapiens C-type lectin domain family 4, member F (CL	+	+	+	+	++	++	+	+
ILMN_2193817	CLEC4G	Homo sapiens C-type lectin superfamily 4, member G (CLEC	+	+	+	+	+	+	+	+
ILMN_2322131	CLEC4M	Homo sapiens C-type lectin domain family 4, member M (CI	-	+	+	+	+	+	+	-
ILMN_1780465	CLEC5A	Homo sapiens C-type lectin domain family 5, member A (CL	-	+	+++	++	+	++	+++	+
ILMN_1742962	CLEC6A	Homo sapiens C-type lectin domain family 6, member A (CL	+	+	+	+	+	+	+	+
ILMN_2323992	CLEC7A	Homo sapiens C-type lectin domain family 7, member A (CL	ND	ND	ND	ND	ND	ND	ND	ND
ILMN_2068274	CLEC9A	Homo sapiens C-type lectin domain family 9, member A (CL	ND	ND	ND	ND	ND	ND	ND	ND
ILMN_2415303	CLEC10A	Homo sapiens C-type lectin domain family 10, member A (C	-	+	+	+	+	++	++	++
ILMN_1704797	CLEC10A	Homo sapiens C-type lectin domain family 10, member A (C	+	+	+	+	+	+	+	+
ILMN_1653166	CLEC10A	Homo sapiens C-type lectin domain family 10, member A (C	-	-	-	+	+	+	+	+
ILMN_1807359	CLEC11A	Homo sapiens C-type lectin domain family 11, member A (C	+	+	+	+	+	+	+	-
ILMN_2403228	CLEC12A	Homo sapiens C-type lectin domain family 12, member A (C	-	-	-	-	-	-	+	+
ILMN_2292178	CLEC12A	Homo sapiens C-type lectin domain family 12, member A (C	-	-	-	-	-	-	-	+
ILMN_1711453	CLEC12A	Homo sapiens C-type lectin domain family 12, member A (C	-	-	+	-	-	+	+	+
ILMN_1663142	CLEC12A	Homo sapiens C-type lectin domain family 12, member A (C	-	-	+	-	-	+	+	++
ILMN_1657527	CLEC12B	Homo sapiens C-type lectin domain family 12, member B (C	ND	ND	ND	ND	ND	ND	ND	ND
ILMN_2142185	CLEC14A	Homo sapiens C-type lectin domain family 14, member A (C	+	+	+	+	+	+	+	+
ILMN_1781752	CLEC16A	Homo sapiens C-type lectin domain family 16, member A (C	++	++	+	++	++	+	+	+
ILMN_1782729	CLECL1	Homo sapiens C-type lectin-like 1 (CLECL1)	+	+	+	+	+	+	+	+
ILMN_1662962	COLEC10	Homo sapiens collectin sub-family member 10 (C-type lectin	ND	ND	ND	ND	ND	ND	ND	ND
ILMN_1735192	COLEC11	Homo sapiens collectin sub-family member 11 (COLEC11)	+	+	+	+	-	+	+	+
ILMN_1689088	COLEC12	Homo sapiens collectin sub-family member 12 (COLEC12)	+	++	++	+	+	+	+	-
ILMN_1797001	DDX58	Homo sapiens DEAD (Asp-Glu-Ala-Asp) box polypeptide 58	+	+	+	+	+	+	+	+
ILMN_1706502	EIF2AK2	Homo sapiens eukaryotic translation initiation factor 2-alpha	+	+	+	+	+	+	+	++
ILMN_1781373	IFIH1	Homo sapiens interferon induced with helicase C domain 1 (I	++	+	+	++	++	+	++	++
ILMN_2079655	KLRB1	Homo sapiens killer cell lectin-like receptor subfamily B, me	-	-	+	+	+	+	+	+
ILMN_2055781	KLRF1	Homo sapiens killer cell lectin-like receptor subfamily F, mei	+	+	+	+	+	+	+	+
ILMN_1685521	KLRF1	Homo sapiens killer cell lectin-like receptor subfamily F, mei	+	+	+	+	+	+	+	+
ILMN_1658399	KLRG1	Homo sapiens killer cell lectin-like receptor subfamily G, me	+	+	+	+	+	+	+	+
ILMN_2224657	KLRG2	Homo sapiens killer cell lectin-like receptor subfamily G, me	ND	ND	ND	ND	ND	ND	ND	ND
ILMN_1723978	LGALS1	Homo sapiens lectin, galactoside-binding, soluble, 1 (LGALS	+++	++	++	+++	+++	+++	++	++
ILMN_1776283	LGALS12	Homo sapiens lectin, galactoside-binding, soluble, 12 (LGAL	+	+	+	+	+	+	+	-
ILMN_1687306	LGALS2	Homo sapiens lectin, galactoside-binding, soluble, 2 (LGALS	+	+	+	+	+	+	+	++
ILMN_1803788	LGALS3	Homo sapiens lectin, galactoside-binding, soluble, 3 (galectin	+	++	++	++	++	++	++	+
ILMN_1694034	LGALS4	Homo sapiens lectin, galactoside-binding, soluble, 4 (galectin	+	+	+	+	+	+	+	+
ILMN_1661708	LGALS7	Homo sapiens lectin, galactoside-binding, soluble, 7 (galectin	+	+	+	++	++	++	++	+
ILMN_2356654	LGALS8	Homo sapiens lectin, galactoside-binding, soluble, 8 (LGALS	+	+	++	+	+	++	++	++
ILMN_2353358	LGALS8	Homo sapiens lectin, galactoside-binding, soluble, 8 (LGALS	+	+	+	+	+	+	++	+
ILMN_2266214	LGALS8	Homo sapiens lectin, galactoside-binding, soluble, 8 (LGALS	+	+	+	+	+	+	+	+
ILMN_2412214	LGALS9	Homo sapiens lectin, galactoside-binding, soluble, 9 (LGALS	+	+	+	+	+	+	+	+
ILMN_1715760	LGALS9	Homo sapiens lectin, galactoside-binding, soluble, 9 (LGALS	+	+	+	+	+	+	+	+
ILMN_1757011	LY75	Homo sapiens lymphocyte antigen 75 (LY75)	+	+	+	+	+	+	+	-
ILMN_1762464	MBL2	Homo sapiens mannose-binding lectin (protein C) 2, soluble (	+	+	+	+	+	+	+	+
ILMN_2203926	MRC1	Homo sapiens mannose receptor, C type 1 (MRC1)	ND	ND	ND	ND	ND	ND	ND	ND
ILMN_1810264	MRC1	Homo sapiens mannose receptor, C type 1 (MRC1)	+	+	+	+	+	+	+	-
ILMN_1683806	MRC1L1	Homo sapiens mannose receptor, C type 1-like 1 (MRC1L1)	+	+	+	+	+	+	+	+
ILMN_1783946	MRC2	Homo sapiens mannose receptor, C type 2 (MRC2)	+	-	+	+	+	+	+	-
ILMN_1723035	OLR1	Homo sapiens oxidized low density lipoprotein (lectin-like) r	-	+	+	++	+	++	++	+
ILMN_1704424	PLA2R1	Homo sapiens phospholipase A2 receptor 1, 180kDa (PLA2R	ND	ND	ND	ND	ND	ND	ND	ND
ILMN_2193892	SELE	Homo sapiens selectin E (endothelial adhesion molecule 1) (S	+	+	+	+	+	+	+	+
ILMN_1739393	SELE	Homo sapiens selectin E (endothelial adhesion molecule 1) (S	-	-	-	+	+	+	+	-
ILMN_1724422	SELL	Homo sapiens selectin L (SELL)	-	-	-	-	-	-	-	++
ILMN_1715417	SELP	Homo sapiens selectin P (granule membrane protein 140kDa,	-	-	-	+	+	+	+	-
ILMN_1678533	SFTPA1	Homo sapiens surfactant protein A1 (SFTPA1)	+	+	+	+	+	+	+	-
ILMN_2048659	SFTPA1B	Homo sapiens surfactant protein A1B (SFTPA1B)	ND	ND	ND	ND	ND	ND	ND	ND
ILMN_2205012	SFTPA2	Homo sapiens surfactant, pulmonary-associated protein A2 (S	ND	ND	ND	ND	ND	ND	ND	ND
ILMN_1768575	SFTPD	Homo sapiens surfactant protein D (SFTPD)	+	+	+	+	+	+	+	+
ILMN_1731048	TLR1	Homo sapiens toll-like receptor 1 (TLR1)	+	+	+	+	+	+	+	+
ILMN_2414762	TLR10	Homo sapiens toll-like receptor 10 (TLR10)	-	-	-	-	-	-	-	+
ILMN_1719905	TLR10	Homo sapiens toll-like receptor 10 (TLR10)	-	-	-	-	-	-	-	+

ILMN_1772387	TLR2	Homo sapiens toll-like receptor 2 (TLR2)	-	+	+	+	+	+	+	+
ILMN_2155708	TLR3	Homo sapiens toll-like receptor 3 (TLR3)	ND	ND	ND	ND	ND	ND	ND	ND
ILMN_1689578	TLR3	Homo sapiens toll-like receptor 3 (TLR3)	-	-	-	+	+	+	+	+
ILMN_1706217	TLR4	Homo sapiens toll-like receptor 4 (TLR4)	-	+	-	+	+	+	+	+
ILMN_1722981	TLR5	Homo sapiens toll-like receptor 5 (TLR5)	-	+	-	+	+	+	+	+
ILMN_1749287	TLR6	Homo sapiens toll-like receptor 6 (TLR6)	+	+	+	+	+	+	+	+
ILMN_1654560	TLR6	Homo sapiens toll-like receptor 6 (TLR6)	+	+	+	+	+	+	+	+
ILMN_1677827	TLR7	Homo sapiens toll-like receptor 7 (TLR7)	-	-	-	+	+	+	+	+
ILMN_1705047	TLR8	Homo sapiens toll-like receptor 8 (TLR8)	+	+	+	+	+	+	+	+
ILMN_1657892	TLR8	Homo sapiens toll-like receptor 8 (TLR8)	-	+	+	+	+	+	+	+
ILMN_1679798	TLR9	Homo sapiens toll-like receptor 9 (TLR9)	ND	ND	ND	ND	ND	ND	ND	ND

ND: not detected; -: <10; +: <100; ++: <500; +++: >= 500
