# Supplementary material for: Transcriptional fingerprints of antigen-presenting cell subsets in the human vaginal mucosa and skin reflect tissue-specific immune microenvironments
Source: Genome Med. 2014 Nov 25;6(11):98. doi: 10.1186/s13073-014-0098-y (PMC4268898; doi:10.1186/s13073-014-0098-y)
Supplement: Additional file 18: Figure S18. — Immunofluorescence staining of lectin receptors on human vagina. [file 13073_2014_98_MOESM18_ESM.pdf]

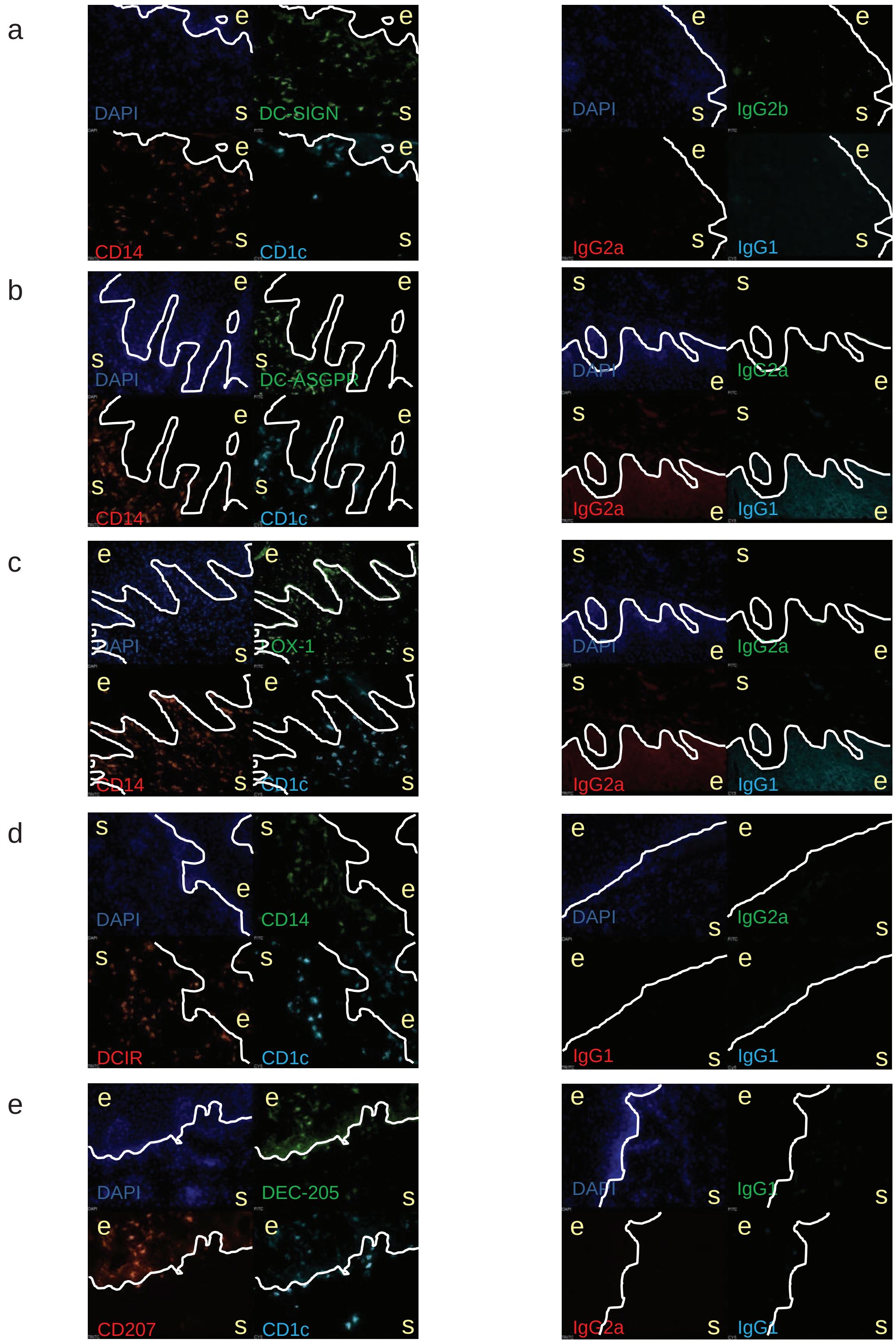

Figure S18: Immunofluorescence staining of lectin receptors on human vagina. a. Frozen tissue sections were stained for DC-SIGN, CD1c and CD14 (left panels) and isotype controls (right panels). **b.** DC-ASGPR, CD1c and CD14 (left panels) and isotype controls (right panels). **c.** LOX-1, CD1c and CD14 (left panels) and isotype controls (right panels). **d.** DCIR, CD1c and CD14 (left panels) and isotype controls (right panels). **e.** DEC-205, CD1c and CD14 (left panels) and isotype controls (right panels). (x20, bar is 100 mm). e: epithelium; s: submucosa
