# Supplementary material for: Transcriptional fingerprints of antigen-presenting cell subsets in the human vaginal mucosa and skin reflect tissue-specific immune microenvironments
Source: Genome Med. 2014 Nov 25;6(11):98. doi: 10.1186/s13073-014-0098-y (PMC4268898; doi:10.1186/s13073-014-0098-y)
Supplement: Additional file 19: Table S3. — Expression of surface molecules, cytokines and chemokines on all APC subsets. [file 13073_2014_98_MOESM19_ESM.pdf]

Illumina ID	Symbol	Definition	Class	Skin			Vagina				Blood
				sLC	sCD14- DC	sCD14+ DC	vLC	vCD14- DC	vCD14+ DC	vMF	mDC
ILMN_1719820	BDNF	Homo sapiens brain-derived neurotrophic factor (BDNF)	Chemokine	-	-	-	+	+	+	+	-
ILMN_1809543	BDNF	Homo sapiens brain-derived neurotrophic factor (BDNF)	Chemokine	+	+	+	+	+	+	+	+
ILMN_1706644	BDNF	Homo sapiens brain-derived neurotrophic factor (BDNF)	Chemokine	+	+	+	+	+	+	+	+
ILMN_1761910	BDNF	Homo sapiens brain-derived neurotrophic factor (BDNF)	Chemokine	+	+	+	+	+	+	+	+
ILMN_1793988	BDNF	Homo sapiens brain-derived neurotrophic factor (BDNF)	Chemokine	+	+	+	+	+	+	+	+
ILMN_1751276	BDNF	Homo sapiens brain-derived neurotrophic factor (BDNF)	Chemokine	ND	ND	ND	ND	ND	ND	ND	ND
ILMN_1712712	BDNF	Homo sapiens brain-derived neurotrophic factor (BDNF)	Chemokine	ND	ND	ND	ND	ND	ND	ND	ND
ILMN_2086965	CCL1	Homo sapiens chemokine (C-C motif) ligand 1 (CCL1)	Chemokine	ND	ND	ND	ND	ND	ND	ND	ND
ILMN_1725519	CCL11	Homo sapiens chemokine (C-C motif) ligand 11 (CCL11)	Chemokine	-	-	+	+	+	+	+	-
ILMN_1783593	CCL13	Homo sapiens chemokine (C-C motif) ligand 13 (CCL13)	Chemokine	+	+	+	+	+	++	++	+
ILMN_2045324	CCL16	Homo sapiens chemokine (C-C motif) ligand 16 (CCL16)	Chemokine	+	+	+	+	+	+	+	+
ILMN_1710186	CCL17	Homo sapiens chemokine (C-C motif) ligand 17 (CCL17)	Chemokine	+	+	+	+	+	+	+	+
ILMN_1654411	CCL18	Homo sapiens chemokine (C-C motif) ligand 18 (pulmonary an	Chemokine	+	+	+	+	+	+	+	+
ILMN_1769129	CCL19	Homo sapiens chemokine (C-C motif) ligand 19 (CCL19)	Chemokine	+	+	+	+	+	+	+	+
ILMN_1720048	CCL2	Homo sapiens chemokine (C-C motif) ligand 2 (CCL2)	Chemokine	+	-	+	+	+	++	+++	-
ILMN_1657234	CCL20	Homo sapiens chemokine (C-C motif) ligand 20 (CCL20)	Chemokine	-	+++	+++	++	++	++	+++	-
ILMN_1677505	CCL21	Homo sapiens chemokine (C-C motif) ligand 21 (CCL21)	Chemokine	ND	ND	ND	ND	ND	ND	ND	ND
ILMN_1686109	CCL23	Homo sapiens chemokine (C-C motif) ligand 23 (CCL23)	Chemokine	+	+	+	-	+	+	+	+
ILMN_1764030	CCL23	Homo sapiens chemokine (C-C motif) ligand 23 (CCL23)	Chemokine	ND	ND	ND	ND	ND	ND	ND	ND
ILMN_1653766	CCL24	Homo sapiens chemokine (C-C motif) ligand 24 (CCL24)	Chemokine	ND	ND	ND	ND	ND	ND	ND	ND
ILMN_1737817	CCL25	Homo sapiens chemokine (C-C motif) ligand 25 (CCL25)	Chemokine	+	+	+	+	+	+	+	+
ILMN_1782596	CCL25	Homo sapiens chemokine (C-C motif) ligand 25 (CCL25)	Chemokine	ND	ND	ND	ND	ND	ND	ND	ND
ILMN_1659601	CCL26	Homo sapiens chemokine (C-C motif) ligand 26 (CCL26)	Chemokine	+	+	+	+	+	+	+	+
ILMN_2113606	CCL27	Homo sapiens chemokine (C-C motif) ligand 27 (CCL27)	Chemokine	-	-	-	+	+	+	+	-
ILMN_1701347	CCL28	Homo sapiens chemokine (C-C motif) ligand 28 (CCL28)	Chemokine	+	+	+	-	+	+	+	+
ILMN_1774087	CCL28	Homo sapiens chemokine (C-C motif) ligand 28 (CCL28)	Chemokine	+	+	+	-	+	+	+	+
ILMN_2228845	CCL28	Homo sapiens chemokine (C-C motif) ligand 28 (CCL28)	Chemokine	+	+	+	+	+	+	+	+
ILMN_1671509	CCL3	Homo sapiens chemokine (C-C motif) ligand 3 (CCL3)	Chemokine	+	++	++	+	+	+	++	+
ILMN_1674563	CCL4	Homo sapiens chemokine (C-C motif) ligand 4 (CCL4)	Chemokine	ND	ND	ND	ND	ND	ND	ND	ND
ILMN_1773352	CCL5	Homo sapiens chemokine (C-C motif) ligand 5 (CCL5)	Chemokine	+	+	+	+	+	+	+	+
ILMN_2098126	CCL5	Homo sapiens chemokine (C-C motif) ligand 5 (CCL5)	Chemokine	++	+++	++	++	++	++	++	++
ILMN_1683456	CCL7	Homo sapiens chemokine (C-C motif) ligand 7 (CCL7)	Chemokine	-	-	+	+	+	+	+	-
ILMN_1772964	CCL8	Homo sapiens chemokine (C-C motif) ligand 8 (CCL8)	Chemokine	-	+	-	+	+	+	+	+
ILMN_2322768	CSF3	Homo sapiens colony stimulating factor 3 (granulocyte) (CSF3	Chemokine	-	-	+	+	+	+	+	-
ILMN_1697069	CSF3	Homo sapiens colony stimulating factor 3 (granulocyte) (CSF3	Chemokine	-	-	+	+	+	+	+	-
ILMN_1706852	CSF3	Homo sapiens colony stimulating factor 3 (granulocyte) (CSF3	Chemokine	ND	ND	ND	ND	ND	ND	ND	ND
ILMN_1654072	CX3CL1	Homo sapiens chemokine (C-X3-C motif) ligand 1 (CX3CL1)	Chemokine	+	+	+	+	+	+	+	+
ILMN_1787897	CXCL1	Homo sapiens chemokine (C-X-C motif) ligand 1 (melanoma g	Chemokine	+	++	+++	+	+	++	++	+
ILMN_1791759	CXCL10	Homo sapiens chemokine (C-X-C motif) ligand 10 (CXCL10)	Chemokine	-	-	-	-	-	+	+	++
ILMN_2067890	CXCL11	Homo sapiens chemokine (C-X-C motif) ligand 11 (CXCL11)	Chemokine	+	+	+	+	+	+	+	+
ILMN_2067895	CXCL11	Homo sapiens chemokine (C-X-C motif) ligand 11 (CXCL11)	Chemokine	ND	ND	ND	ND	ND	ND	ND	ND
ILMN_1791447	CXCL12	Homo sapiens chemokine (C-X-C motif) ligand 12 (stromal cel	Chemokine	-	-	-	+	+	+	+	+
ILMN_1803825	CXCL12	Homo sapiens chemokine (C-X-C motif) ligand 12 (stromal cel	Chemokine	+	+	+	+	+	+	-	+
ILMN_1689111	CXCL12	Homo sapiens chemokine (C-X-C motif) ligand 12 (stromal cel	Chemokine	ND	ND	ND	ND	ND	ND	ND	ND
ILMN_1718552	CXCL13	Homo sapiens chemokine (C-X-C motif) ligand 13 (B-cell che	Chemokine	+	++	+	+	+	+	+	+
ILMN_1748323	CXCL14	Homo sapiens chemokine (C-X-C motif) ligand 14 (CXCL14)	Chemokine	-	+	+	+	+	+	+	+
ILMN_1728478	CXCL16	Homo sapiens chemokine (C-X-C motif) ligand 16 (CXCL16)	Chemokine	+++	+++	+++	+++	+++	+++	+++	+++
ILMN_1672278	CXCL16	Homo sapiens chemokine (C-X-C motif) ligand 16 (CXCL16)	Chemokine	ND	ND	ND	ND	ND	ND	ND	ND
ILMN_1682636	CXCL2	Homo sapiens chemokine (C-X-C motif) ligand 2 (CXCL2)	Chemokine	-	++	+++	+	+	++	++	+
ILMN_1709350	CXCL3	Homo sapiens chemokine (C-X-C motif) ligand 3 (CXCL3)	Chemokine	+	+	+	+	+	+	+	+
ILMN_1752562	CXCL5	Homo sapiens chemokine (C-X-C motif) ligand 5 (CXCL5)	Chemokine	+	++	+++	++	++	++	+++	+
ILMN_2171384	CXCL5	Homo sapiens chemokine (C-X-C motif) ligand 5 (CXCL5)	Chemokine	+	+++	+++	++	+	+++	+++	+
ILMN_1779234	CXCL6	Homo sapiens chemokine (C-X-C motif) ligand 6 (granulocyte	Chemokine	+	+	+	-	-	-	+	-
ILMN_2161577	CXCL6	Homo sapiens chemokine (C-X-C motif) ligand 6 (granulocyte	Chemokine	+	+	+	-	-	-	-	-
ILMN_1745356	CXCL9	Homo sapiens chemokine (C-X-C motif) ligand 9 (CXCL9)	Chemokine	+	+	+	+	+	+	+	+
ILMN_1681283	HIF1A	Homo sapiens hypoxia-inducible factor 1, alpha subunit (basic	Chemokine	+	+	+	+	+	+	+	+
ILMN_1763260	HIF1A	Homo sapiens hypoxia-inducible factor 1, alpha subunit (basic	Chemokine	+	+	+	-	-	-	+	+
ILMN_2379788	HIF1A	Homo sapiens hypoxia-inducible factor 1, alpha subunit (basic	Chemokine	+	+	+	+	+	+	+	++
ILMN_1673711	HSP90AB1	Homo sapiens heat shock protein 90kDa alpha (cytosolic), class	Chemokine	++	++	++	++	++	++	++	++
ILMN_2052511	IL13	Homo sapiens interleukin 13 (IL13)	Chemokine	ND	ND	ND	ND	ND	ND	ND	ND
ILMN_1813572	IL16	Homo sapiens interleukin 16 (lymphocyte chemoattractant fact	Chemokine	-	-	-	-	-	-	-	+
ILMN_2290628	IL16	Homo sapiens interleukin 16 (lymphocyte chemoattractant fact	Chemokine	-	-	-	-	-	-	-	+
ILMN_1778457	IL18	Homo sapiens interleukin 18 (interferon-gamma-inducing facto	Chemokine	+++	+++	+++	+++	+++	+++	+++	+++
ILMN_1658483	IL1A	Homo sapiens interleukin 1, alpha (IL1A)	Chemokine	+	++	++	+	+	+	++	+
ILMN_2389080	IL4	Homo sapiens interleukin 4 (IL4)	Chemokine	ND	ND	ND	ND	ND	ND	ND	ND
ILMN_1669174	IL4	Homo sapiens interleukin 4 (IL4)	Chemokine	ND	ND	ND	ND	ND	ND	ND	ND
ILMN_1666733	IL8	Homo sapiens interleukin 8 (IL8)	Chemokine	+++	+++	+++	+++	+++	+++	+++	+++
ILMN_2184373	IL8	Homo sapiens interleukin 8 (IL8)	Chemokine	+++	+++	+++	+++	+++	+++	+++	+++
ILMN_1762106	MMP2	Homo sapiens matrix metallopeptidase 2 (gelatinase A, 72kDa	Chemokine	ND	ND	ND	ND	ND	ND	ND	ND
ILMN_1685403	MMP7	Homo sapiens matrix metallopeptidase 7 (matrilysin, uterine) (	Chemokine	+	+	+	+	+	+	+	-
ILMN_2192072	MMP7	Homo sapiens matrix metallopeptidase 7 (matrilysin, uterine) (	Chemokine	+	+	+	+	+	+	+	-
ILMN_1655414	TNFSF14	Homo sapiens tumor necrosis factor (ligand) superfamily, mem	Chemokine	+	+	+	+	+	+	+	-
ILMN_2363392	TNFSF14	Homo sapiens tumor necrosis factor (ligand) superfamily, mem	Chemokine	++	++	++	++	++	++	++	++
ILMN_1661343	TNFSF14	Homo sapiens tumor necrosis factor (ligand) superfamily, mem	Chemokine	ND	ND	ND	ND	ND	ND	ND	ND
ILMN_2224955	XCL1	Homo sapiens chemokine (C motif) ligand 1 (XCL1)	Chemokine	+	+	+	+	+	+	+	+
ILMN_1718792	XCL1	Homo sapiens chemokine (C motif) ligand 1 (XCL1)	Chemokine	ND	ND	ND	ND	ND	ND	ND	ND
ILMN_1699669	BLR1	Homo sapiens Burkitt lymphoma receptor 1, GTP binding prote	Chemokine Receptor	ND	ND	ND	ND	ND	ND	ND	ND
ILMN_1678833	CCR1	Homo sapiens chemokine (C-C motif) receptor 1 (CCR1)	Chemokine Receptor	-	-	+	+	+	+	++	+
ILMN_1666493	CCR10	Homo sapiens chemokine (C-C motif) receptor 10 (CCR10)	Chemokine Receptor	+	+	+	+	+	+	+	+
ILMN_2376431	CCR2	Homo sapiens chemokine (C-C motif) receptor 2 (CCR2)	Chemokine Receptor	+	+	+	+	+	+	+	-
ILMN_1658299	CCR2	Homo sapiens chemokine (C-C motif) receptor 2 (CCR2)	Chemokine Receptor	+	+	+	+	+	+	+	-
ILMN_1774761	CCR2	Homo sapiens chemokine (C-C motif) receptor 2 (CCR2)	Chemokine Receptor	+	+	+	-	+	-	+	+
ILMN_1669062	CCR2	Homo sapiens chemokine (C-C motif) receptor 2 (CCR2)	Chemokine Receptor	+	+	+	+	+	+	+	+
ILMN_1769895	CCR2	Homo sapiens chemokine (C-C motif) receptor 2 (CCR2)	Chemokine Receptor	+	+	+	+	+	+	+	+
ILMN_1776706	CCR2	Homo sapiens chemokine (C-C motif) receptor 2 (CCR2)	Chemokine Receptor	ND	ND	ND	ND	ND	ND	ND	ND
ILMN_1777461	CCR2	Homo sapiens chemokine (C-C motif) receptor 2 (CCR2)	Chemokine Receptor	ND	ND	ND	ND	ND	ND	ND	ND
ILMN_2276996	CCR2	Homo sapiens chemokine (C-C motif) receptor 2 (CCR2)	Chemokine Receptor	ND	ND	ND	ND	ND	ND	ND	ND
ILMN_1763322	CCR3	Homo sapiens chemokine (C-C motif) receptor 3 (CCR3)	Chemokine Receptor	ND	ND	ND	ND	ND	ND	ND	ND
ILMN_1656684	CCR3	Homo sapiens chemokine (C-C motif) receptor 3 (CCR3)	Chemokine Receptor	ND	ND	ND	ND	ND	ND	ND	ND
ILMN_2086143	CCR4	Homo sapiens chemokine (C-C motif) receptor 4 (CCR4)	Chemokine Receptor	ND	ND	ND	ND	ND	ND	ND	ND
ILMN_2145033	CCR5	Homo sapiens chemokine (C-C motif) receptor 5 (CCR5)	Chemokine Receptor	ND	ND	ND	ND	ND	ND	ND	ND
ILMN_1653395	CCR5	Homo sapiens chemokine (C-C motif) receptor 5 (CCR5)	Chemokine Receptor	ND	ND	ND	ND	ND	ND	ND	ND
ILMN_1759635	CCR6	Homo sapiens chemokine (C-C motif) receptor 6 (CCR6)	Chemokine Receptor	-	-	-	-	-	-	-	+
ILMN_1690907	CCR6	Homo sapiens chemokine (C-C motif) receptor 6 (CCR6)	Chemokine Receptor	+	+	+	+	+	+	+	+
ILMN_2387696	CCR6	Homo sapiens chemokine (C-C motif) receptor 6 (CCR6)	Chemokine Receptor	+++	+++	+++	+++	+++	+++	+++	+++
ILMN_1715131	CCR7	Homo sapiens chemokine (C-C motif) receptor 7 (CCR7)	Chemokine Receptor	+++	+++	+++	+++	+++	+++	+++	+++
ILMN_1739421	CCR8	Homo sapiens chemokine (C-C motif) receptor 8 (CCR8)	Chemokine Receptor	+	+	+	+	+	+	+	-
ILMN_2337386	CCR9	Homo sapiens chemokine (C-C motif) receptor 9 (CCR9)	Chemokine Receptor	-	-	-	+	+	+	-	+
ILMN_1664316	CCR9	Homo sapiens chemokine (C-C motif) receptor 9 (CCR9)	Chemokine Receptor	+	+	+	+	+	+	+	+
ILMN_1781769	CCRL1	Homo sapiens chemokine (C-C motif) receptor-like 1 (CCRL1)	Chemokine Receptor	-	-	-	+	+	+	+	-
ILMN_2331922	CCRL1	Homo sapiens chemokine (C-C motif) receptor-like 1 (CCRL1)	Chemokine Receptor	-	-	-	-	-	-	-	+
ILMN_1740436	CCRL1	Homo sapiens chemokine (C-C motif) receptor-like 1 (CCRL1)	Chemokine Receptor	ND	ND	ND	ND	ND	ND	ND	ND
ILMN_1773992	CCRL1	Homo sapiens chemokine (C-C motif) receptor-like 1 (CCRL1)	Chemokine Receptor	ND	ND	ND	ND	ND	ND	ND	ND

ILMN_1799030	CMTM2	Homo sapiens CKLF-like MARVEL transmembrane domain co	Chemokine Receptor	-	-	-	+	+	+	+	+
ILMN_1679277	CMTM3	Homo sapiens CKLF-like MARVEL transmembrane domain co	Chemokine Receptor	-	-	-	+	+	+	+	-
ILMN_1667840	CMTM3	Homo sapiens CKLF-like MARVEL transmembrane domain co	Chemokine Receptor	+	+	+	+	+	+	+	+
ILMN_2370208	CMTM3	Homo sapiens CKLF-like MARVEL transmembrane domain co	Chemokine Receptor	+	+	+	+	+	+	+	+
ILMN_1705442	CMTM3	Homo sapiens CKLF-like MARVEL transmembrane domain co	Chemokine Receptor	+	+	+	+	+	++	++	++
ILMN_1762718	CMTM4	Homo sapiens CKLF-like MARVEL transmembrane domain co	Chemokine Receptor	+	+	+	+	+	+	+	-
ILMN_1815319	CMTM4	Homo sapiens CKLF-like MARVEL transmembrane domain co	Chemokine Receptor	+	+	+	+	+	+	+	+
ILMN_1729514	CMTM4	Homo sapiens CKLF-like MARVEL transmembrane domain co	Chemokine Receptor	ND	ND	ND	ND	ND	ND	ND	ND
ILMN_1720930	CMTM4	Homo sapiens CKLF-like MARVEL transmembrane domain co	Chemokine Receptor	ND	ND	ND	ND	ND	ND	ND	ND
ILMN_1696494	CMTM6	Homo sapiens CKLF-like MARVEL transmembrane domain co	Chemokine Receptor	+++	++	++	++	++	++	++	+++
ILMN_1740487	CMTM7	Homo sapiens CKLF-like MARVEL transmembrane domain co	Chemokine Receptor	+	-	+	+	+	+	+	-
ILMN_2403247	CMTM7	Homo sapiens CKLF-like MARVEL transmembrane domain co	Chemokine Receptor	+	+	+	+	+	+	+	+
ILMN_1698934	CMTM7	Homo sapiens CKLF-like MARVEL transmembrane domain co	Chemokine Receptor	+++	++	++	+++	++	+++	+++	++
ILMN_1710124	CMTM8	Homo sapiens CKLF-like MARVEL transmembrane domain co	Chemokine Receptor	+	+	-	+	+	+	+	-
ILMN_1745788	CX3CR1	Homo sapiens chemokine (C-X3-C motif) receptor 1 (CX3CR1	Chemokine Receptor	+	+	+	+	+	+	+	+
ILMN_2088437	CX3CR1	Homo sapiens chemokine (C-X3-C motif) receptor 1 (CX3CR1	Chemokine Receptor	+	+	+	-	+	+	+	+
ILMN_1662524	CXCR1	Homo sapiens chemokine (C-X-C motif) receptor 1 (CXCR1)	Chemokine Receptor	-	-	+	+	+	+	+	-
ILMN_1797975	CXCR3	Homo sapiens chemokine (C-X-C motif) receptor 3 (CXCR3)	Chemokine Receptor	+	+	+	+	+	+	+	+
ILMN_2246410	CXCR4	Homo sapiens chemokine (C-X-C motif) receptor 4 (CXCR4)	Chemokine Receptor	+	+	+	-	+	+	+	+
ILMN_1801584	CXCR4	Homo sapiens chemokine (C-X-C motif) receptor 4 (CXCR4)	Chemokine Receptor	+	+	+	+	+	+	+	+
ILMN_2320888	CXCR4	Homo sapiens chemokine (C-X-C motif) receptor 4 (CXCR4)	Chemokine Receptor	+	++	++	+	+	+	+	++
ILMN_2337931	CXCR5	Homo sapiens chemokine (C-X-C motif) receptor 5 (CXCR5)	Chemokine Receptor	+	+	+	+	+	+	+	+
ILMN_2337928	CXCR5	Homo sapiens chemokine (C-X-C motif) receptor 5 (CXCR5)	Chemokine Receptor	+	+	+	+	+	+	+	+
ILMN_1674640	CXCR6	Homo sapiens chemokine (C-X-C motif) receptor 6 (CXCR6)	Chemokine Receptor	-	-	+	+	+	+	+	-
ILMN_2371458	CXCR7	Homo sapiens chemokine (C-X-C motif) receptor 7 (CXCR7)	Chemokine Receptor	-	+	++	+	+	+	+	+
ILMN_1798360	CXCR7	Homo sapiens chemokine (C-X-C motif) receptor 7 (CXCR7)	Chemokine Receptor	+	++	++	+	+	++	++	+
ILMN_1680397	IL8RB	Homo sapiens interleukin 8 receptor, beta (IL8RB)	Chemokine Receptor	+	+	+	+	+	+	+	+
ILMN_1747251	LTB4R	Homo sapiens leukotriene B4 receptor (LTB4R)	Chemokine Receptor	+	+	+	+	+	+	+	+
ILMN_1685005	TNFRSF1A	Homo sapiens tumor necrosis factor receptor superfamily, mem	Chemokine Receptor	++	++	++	++	++	++	++	++
ILMN_1688231	TREM1	Homo sapiens triggering receptor expressed on myeloid cells 1	Chemokine Receptor	+	++	++	+	+	++	++	+
ILMN_1701248	TREM2	Homo sapiens triggering receptor expressed on myeloid cells 2	Chemokine Receptor	+	+	+	-	-	+	+	+
ILMN_1764034	XCR1	Homo sapiens chemokine (C motif) receptor 1 (XCR1)	Chemokine Receptor	+	-	+	+	+	+	+	+
ILMN_2407954	XCR1	Homo sapiens chemokine (C motif) receptor 1 (XCR1)	Chemokine Receptor	ND	ND	ND	ND	ND	ND	ND	ND
ILMN_1773238	XCR1	Homo sapiens chemokine (C motif) receptor 1 (XCR1)	Chemokine Receptor	ND	ND	ND	ND	ND	ND	ND	ND
ILMN_1779257	CD40	Homo sapiens CD40 molecule, TNF receptor superfamily mem	Costimulation	+	+	+	+	+	+	+	+
ILMN_2367818	CD40	Homo sapiens CD40 molecule, TNF receptor superfamily mem	Costimulation	+	+	+	+	+	+	+	+
ILMN_1716736	CD80	Homo sapiens CD80 molecule (CD80)	Costimulation	+	+	+	+	+	+	+	-
ILMN_1782560	CD86	Homo sapiens CD86 antigen (CD28 antigen ligand 2, B7-2 anti	Costimulation	+	+	+	+	+	+	+	+
ILMN_1714602	CD86	Homo sapiens CD86 molecule (CD86)	Costimulation	+++	+++	+++	+++	+++	+++	+++	+++
ILMN_1672097	CD86	Homo sapiens CD86 antigen (CD28 antigen ligand 2, B7-2 anti	Costimulation	ND	ND	ND	ND	ND	ND	ND	ND
ILMN_1675671	ICOSLG	Homo sapiens inducible T-cell co-stimulator ligand (ICOSLG)	Costimulation	+	+	+	+	+	+	+	+
ILMN_2089875	TNFSF4	Homo sapiens tumor necrosis factor (ligand) superfamily, mem	Costimulation	++	+	+	+	+	+	+	+
ILMN_1746175	TNFSF4	Homo sapiens tumor necrosis factor (ligand) superfamily, mem	Costimulation	++	+	+	+	+	+	+	+
ILMN_1751464	TNFSF9	Homo sapiens tumor necrosis factor (ligand) superfamily, mem	Costimulation	+	+	+	+	+	+	+	+
ILMN_1804421	AREG	Homo sapiens amphiregulin (AREG)	Cytokine	ND	ND	ND	ND	ND	ND	ND	ND
ILMN_1697694	ATP6AP1	Homo sapiens ATPase, H+ transporting, lysosomal accessory pr	Cytokine	+++	+++	+++	+++	+++	+++	+++	+++
ILMN_1791688	BMP10	Homo sapiens bone morphogenetic protein 10 (BMP10)	Cytokine	ND	ND	ND	ND	ND	ND	ND	ND
ILMN_1702755	BMP15	Homo sapiens bone morphogenetic protein 15 (BMP15)	Cytokine	+	-	+	+	+	+	+	-
ILMN_1722718	BMP2	Homo sapiens bone morphogenetic protein 2 (BMP2)	Cytokine	+	+	+	+	+	+	+	+
ILMN_1682661	BMP3	Homo sapiens bone morphogenetic protein 3 (BMP3)	Cytokine	ND	ND	ND	ND	ND	ND	ND	ND
ILMN_1660552	BMP5	Homo sapiens bone morphogenetic protein 5 (BMP5)	Cytokine	ND	ND	ND	ND	ND	ND	ND	ND
ILMN_1747650	BMP6	Homo sapiens bone morphogenetic protein 6 (BMP6)	Cytokine	+	++	++	+	+	+	+	-
ILMN_1741566	BMP7	Homo sapiens bone morphogenetic protein 7 (osteogenic protei	Cytokine	+	+	+	+	+	+	+	+
ILMN_2123415	BMP8B	Homo sapiens bone morphogenetic protein 8b (BMP8B)	Cytokine	-	-	-	+	+	+	+	+
ILMN_1659077	CD40LG	Homo sapiens CD40 ligand (CD40LG)	Cytokine	ND	ND	ND	ND	ND	ND	ND	ND
ILMN_1760247	CD70	Homo sapiens CD70 molecule (CD70)	Cytokine	+	+	+	+	+	+	+	+
ILMN_2302947	CSF1	Homo sapiens colony stimulating factor 1 (macrophage) (CSF1	Cytokine	ND	ND	ND	ND	ND	ND	ND	ND
ILMN_1805930	CSF1	Homo sapiens colony stimulating factor 1 (macrophage) (CSF1	Cytokine	ND	ND	ND	ND	ND	ND	ND	ND
ILMN_1733402	CSF1	Homo sapiens colony stimulating factor 1 (macrophage) (CSF1	Cytokine	ND	ND	ND	ND	ND	ND	ND	ND
ILMN_1661861	CSF2	Homo sapiens colony stimulating factor 2 (granulocyte-macrop	Cytokine	+	+	+	+	+	+	+	+
ILMN_2322768	CSF3	Homo sapiens colony stimulating factor 3 (granulocyte) (CSF3	Cytokine	-	-	+	+	+	+	+	-
ILMN_1697069	CSF3	Homo sapiens colony stimulating factor 3 (granulocyte) (CSF3	Cytokine	-	-	+	+	+	+	+	-
ILMN_1706852	CSF3	Homo sapiens colony stimulating factor 3 (granulocyte) (CSF3	Cytokine	ND	ND	ND	ND	ND	ND	ND	ND
ILMN_1691276	CXXC1	Homo sapiens CXXC finger 1 (PHD domain) (CXXC1)	Cytokine	+++	+++	+++	+++	+++	+++	+++	++
ILMN_1685767	FAM3B	Homo sapiens family with sequence similarity 3, member B (FA	Cytokine	-	-	-	+	+	+	+	-
ILMN_2355486	FAM3B	Homo sapiens family with sequence similarity 3, member B (FA	Cytokine	-	-	+	+	+	+	+	-
ILMN_1777261	FAM3C	Homo sapiens family with sequence similarity 3, member C (FA	Cytokine	+	+	+	+	+	+	+	-
ILMN_2368773	FAM3C	Homo sapiens family with sequence similarity 3, member C (FA	Cytokine	+	+	+	+	-	-	+	+
ILMN_1781824	FASLG	Homo sapiens Fas ligand (TNF superfamily, member 6) (FASL	Cytokine	ND	ND	ND	ND	ND	ND	ND	ND
ILMN_2110110	FGF10	Homo sapiens fibroblast growth factor 10 (FGF10)	Cytokine	ND	ND	ND	ND	ND	ND	ND	ND
ILMN_1707612	FIGF	Homo sapiens c-fos induced growth factor (vascular endothelia	Cytokine	+	+	+	+	+	+	+	+
ILMN_2091412	FLT3LG	Homo sapiens fms-related tyrosine kinase 3 ligand (FLT3LG)	Cytokine	-	-	-	+	+	+	+	-
ILMN_1678191	GDF10	Homo sapiens growth differentiation factor 10 (GDF10)	Cytokine	ND	ND	ND	ND	ND	ND	ND	ND
ILMN_1745132	GDF11	Homo sapiens growth differentiation factor 11 (GDF11)	Cytokine	+	+	+	+	+	+	+	+
ILMN_2188862	GDF15	Homo sapiens growth differentiation factor 15 (GDF15)	Cytokine	+	+	+	+	+	+	+	+
ILMN_1760363	GDF2	Homo sapiens growth differentiation factor 2 (GDF2)	Cytokine	ND	ND	ND	ND	ND	ND	ND	ND
ILMN_2210111	GDF3	Homo sapiens growth differentiation factor 3 (GDF3)	Cytokine	ND	ND	ND	ND	ND	ND	ND	ND
ILMN_1796471	GDF5	Homo sapiens growth differentiation factor 5 (GDF5)	Cytokine	ND	ND	ND	ND	ND	ND	ND	ND
ILMN_1738783	GDF9	Homo sapiens growth differentiation factor 9 (GDF9)	Cytokine	+	+	+	+	+	+	+	+
ILMN_1688663	IFNA1	Homo sapiens interferon, alpha 1 (IFNA1)	Cytokine	ND	ND	ND	ND	ND	ND	ND	ND
ILMN_2193942	IFNA13	Homo sapiens interferon, alpha 13 (IFNA13)	Cytokine	ND	ND	ND	ND	ND	ND	ND	ND
ILMN_1664595	IFNA13	Homo sapiens interferon, alpha 13 (IFNA13)	Cytokine	ND	ND	ND	ND	ND	ND	ND	ND
ILMN_2129202	IFNA14	Homo sapiens interferon, alpha 14 (IFNA14)	Cytokine	ND	ND	ND	ND	ND	ND	ND	ND
ILMN_1742328	IFNA14	Homo sapiens interferon, alpha 14 (IFNA14)	Cytokine	ND	ND	ND	ND	ND	ND	ND	ND
ILMN_1698186	IFNA2	Homo sapiens interferon, alpha 2 (IFNA2)	Cytokine	ND	ND	ND	ND	ND	ND	ND	ND
ILMN_1782448	IFNA4	Homo sapiens interferon, alpha 4 (IFNA4)	Cytokine	+	+	+	+	+	+	+	+
ILMN_2202096	IFNA4	Homo sapiens interferon, alpha 4 (IFNA4)	Cytokine	ND	ND	ND	ND	ND	ND	ND	ND
ILMN_1660195	IFNA5	Homo sapiens interferon, alpha 5 (IFNA5)	Cytokine	ND	ND	ND	ND	ND	ND	ND	ND
ILMN_2110561	IFNA8	Homo sapiens interferon, alpha 8 (IFNA8)	Cytokine	ND	ND	ND	ND	ND	ND	ND	ND
ILMN_1678574	IFNA8	Homo sapiens interferon, alpha 8 (IFNA8)	Cytokine	ND	ND	ND	ND	ND	ND	ND	ND
ILMN_1682245	IFNB1	Homo sapiens interferon, beta 1, fibroblast (IFNB1)	Cytokine	ND	ND	ND	ND	ND	ND	ND	ND
ILMN_2207291	IFNG	Homo sapiens interferon, gamma (IFNG)	Cytokine	+	-	+	+	+	+	+	+
ILMN_1676704	IFNK	Homo sapiens interferon, kappa (IFNK)	Cytokine	ND	ND	ND	ND	ND	ND	ND	ND
ILMN_1781057	IFNW1	Homo sapiens interferon, omega 1 (IFNW1)	Cytokine	+	+	+	+	+	+	+	+
ILMN_2176225	IFNW1	Homo sapiens interferon, omega 1 (IFNW1)	Cytokine	ND	ND	ND	ND	ND	ND	ND	ND
ILMN_1699362	IK	Homo sapiens IK cytokine, down-regulator of HLA II (IK)	Cytokine	++	++	++	++	++	++	++	++
ILMN_1674167	IL10	Homo sapiens interleukin 10 (IL10)	Cytokine	+	+	+	+	+	+	+	+
ILMN_2073307	IL10	Homo sapiens interleukin 10 (IL10)	Cytokine	+++	+++	+++	+++	+++	+++	+++	+++
ILMN_1788107	IL11	Homo sapiens interleukin 11 (IL11)	Cytokine	+	+	+	+	+	+	+	+
ILMN_1671353	IL12A	Homo sapiens interleukin 12A (natural killer cell stimulatory fa	Cytokine	+	+	-	-	-	-	-	+
ILMN_1681132	IL12B	Homo sapiens interleukin 12B (natural killer cell stimulatory fa	Cytokine	ND	ND	ND	ND	ND	ND	ND	ND
ILMN_2052511	IL13	Homo sapiens interleukin 13 (IL13)	Cytokine	ND	ND	ND	ND	ND	ND	ND	ND
ILMN_1813572	IL16	Homo sapiens interleukin 16 (lymphocyte chemoattractant facto	Cytokine	-	-	-	-	-	-	-	+
ILMN_2290628	IL16	Homo sapiens interleukin 16 (lymphocyte chemoattractant facto	Cytokine	-	-	-	-	-	-	-	+
ILMN_1774983	IL17A	Homo sapiens interleukin 17A (IL17A)	Cytokine	+	-	+	+	+	+	+	-

ILMN_1766707	IL17B	Homo sapiens interleukin 17B (IL17B)	Cytokine	ND	ND	ND	ND	ND	ND	ND	ND
ILMN_1788109	IL17C	Homo sapiens interleukin 17C (IL17C)	Cytokine	ND	ND	ND	ND	ND	ND	ND	ND
ILMN_2188247	IL17F	Homo sapiens interleukin 17F (IL17F)	Cytokine	ND	ND	ND	ND	ND	ND	ND	ND
ILMN_1778457	IL18	Homo sapiens interleukin 18 (interferon-gamma-inducing facto	Cytokine	+++	+++	+++	+++	+++	+++	+++	+++
ILMN_1799575	IL19	Homo sapiens interleukin 19 (IL19)	Cytokine	+	+	+	-	-	+	+	+
ILMN_1682592	IL19	Homo sapiens interleukin 19 (IL19)	Cytokine	ND	ND	ND	ND	ND	ND	ND	ND
ILMN_1658483	IL1A	Homo sapiens interleukin 1, alpha (IL1A)	Cytokine	+	++	++	+	+	+	++	+
ILMN_1775501	IL1B	Homo sapiens interleukin 1, beta (IL1B)	Cytokine	++	+++	+++	++	++	+++	+++	+++
ILMN_1804901	IL1F5	Homo sapiens interleukin 1 family, member 5 (delta) (IL1F5)	Cytokine	+	+	+	+	+	+	+	+
ILMN_1759141	IL1F5	Homo sapiens interleukin 1 family, member 5 (delta) (IL1F5)	Cytokine	ND	ND	ND	ND	ND	ND	ND	ND
ILMN_1704000	IL1F6	Homo sapiens interleukin 1 family, member 6 (epsilon) (IL1F6	Cytokine	ND	ND	ND	ND	ND	ND	ND	ND
ILMN_2158713	IL1F9	Homo sapiens interleukin 1 family, member 9 (IL1F9)	Cytokine	+	+	+	++	++	+	++	-
ILMN_1704321	IL2	Homo sapiens interleukin 2 (IL2)	Cytokine	ND	ND	ND	ND	ND	ND	ND	ND
ILMN_1772674	IL21	Homo sapiens interleukin 21 (IL21)	Cytokine	ND	ND	ND	ND	ND	ND	ND	ND
ILMN_2100046	IL22	Homo sapiens interleukin 22 (IL22)	Cytokine	ND	ND	ND	ND	ND	ND	ND	ND
ILMN_1735208	IL22	Homo sapiens interleukin 22 (IL22)	Cytokine	ND	ND	ND	ND	ND	ND	ND	ND
ILMN_1715603	IL23A	Homo sapiens interleukin 23, alpha subunit p19 (IL23A)	Cytokine	+	+++	++	+	+	+	+	+
ILMN_1725814	IL24	Homo sapiens interleukin 24 (IL24)	Cytokine	+	+	+	+	+	+	+	-
ILMN_2407799	IL24	Homo sapiens interleukin 24 (IL24)	Cytokine	+	++	+++	+	-	+	++	+
ILMN_1774685	IL24	Homo sapiens interleukin 24 (IL24)	Cytokine	+	+++	+++	+	-	++	+++	+
ILMN_2401883	IL25	Homo sapiens interleukin 25 (IL25)	Cytokine	-	-	-	+	+	+	+	-
ILMN_1720243	IL25	Homo sapiens interleukin 25 (IL25)	Cytokine	+	+	+	+	+	+	+	+
ILMN_2401884	IL25	Homo sapiens interleukin 25 (IL25)	Cytokine	ND	ND	ND	ND	ND	ND	ND	ND
ILMN_2123182	IL26	Homo sapiens interleukin 26 (IL26)	Cytokine	ND	ND	ND	ND	ND	ND	ND	ND
ILMN_1753758	IL27	Homo sapiens interleukin 27 (IL27)	Cytokine	ND	ND	ND	ND	ND	ND	ND	ND
ILMN_1662302	IL28A	Homo sapiens interleukin 28A (interferon, lambda 2) (IL28A)	Cytokine	ND	ND	ND	ND	ND	ND	ND	ND
ILMN_1768900	IL28B	Homo sapiens interleukin 28B (interferon, lambda 3) (IL28B)	Cytokine	ND	ND	ND	ND	ND	ND	ND	ND
ILMN_1684570	IL29	Homo sapiens interleukin 29 (interferon, lambda 1) (IL29)	Cytokine	ND	ND	ND	ND	ND	ND	ND	ND
ILMN_2149624	IL29	Homo sapiens interleukin 29 (interferon, lambda 1) (IL29)	Cytokine	ND	ND	ND	ND	ND	ND	ND	ND
ILMN_1766320	IL3	Homo sapiens interleukin 3 (colony-stimulating factor, multiple	Cytokine	ND	ND	ND	ND	ND	ND	ND	ND
ILMN_2201866	IL31	Homo sapiens interleukin 31 (IL31)	Cytokine	ND	ND	ND	ND	ND	ND	ND	ND
ILMN_2368530	IL32	Homo sapiens interleukin 32 (IL32)	Cytokine	+	-	-	+	+	+	+	-
ILMN_1778010	IL32	Homo sapiens interleukin 32 (IL32)	Cytokine	+	+	-	+	+	+	+	+
ILMN_1809099	IL33	Homo sapiens interleukin 33 (IL33)	Cytokine	ND	ND	ND	ND	ND	ND	ND	ND
ILMN_2052924	IL33	Homo sapiens interleukin 33 (IL33)	Cytokine	ND	ND	ND	ND	ND	ND	ND	ND
ILMN_1713686	IL34	Homo sapiens interleukin 34 (IL34)	Cytokine	ND	ND	ND	ND	ND	ND	ND	ND
ILMN_2389080	IL4	Homo sapiens interleukin 4 (IL4)	Cytokine	ND	ND	ND	ND	ND	ND	ND	ND
ILMN_1669174	IL4	Homo sapiens interleukin 4 (IL4)	Cytokine	ND	ND	ND	ND	ND	ND	ND	ND
ILMN_1709300	IL5	Homo sapiens interleukin 5 (colony-stimulating factor, eosinop	Cytokine	+	+	+	+	+	+	+	+
ILMN_2207190	IL5	Homo sapiens interleukin 5 (colony-stimulating factor, eosinop	Cytokine	ND	ND	ND	ND	ND	ND	ND	ND
ILMN_1699651	IL6	Homo sapiens interleukin 6 (interferon, beta 2) (IL6)	Cytokine	+	+	++	+	+	+	+	+
ILMN_2059744	IL7	Homo sapiens interleukin 7 (IL7)	Cytokine	ND	ND	ND	ND	ND	ND	ND	ND
ILMN_1705769	IL7	Homo sapiens interleukin 7 (IL7)	Cytokine	ND	ND	ND	ND	ND	ND	ND	ND
ILMN_1666733	IL8	Homo sapiens interleukin 8 (IL8)	Cytokine	+++	+++	+++	+++	+++	+++	+++	+++
ILMN_2184373	IL8	Homo sapiens interleukin 8 (IL8)	Cytokine	+++	+++	+++	+++	+++	+++	+++	+++
ILMN_2151676	IL9	Homo sapiens interleukin 9 (IL9)	Cytokine	+	+	+	+	+	+	+	+
ILMN_1653704	IL9	Homo sapiens interleukin 9 (IL9)	Cytokine	ND	ND	ND	ND	ND	ND	ND	ND
ILMN_1756443	INHA	Homo sapiens inhibin, alpha (INHA)	Cytokine	ND	ND	ND	ND	ND	ND	ND	ND
ILMN_1719547	INHBA	Homo sapiens inhibin, beta A (INHBA)	Cytokine	ND	ND	ND	ND	ND	ND	ND	ND
ILMN_1685714	INHBB	Homo sapiens inhibin, beta B (activin AB beta polypeptide) (IN	Cytokine	ND	ND	ND	ND	ND	ND	ND	ND
ILMN_1673232	LASS1	Homo sapiens LAG1 homolog, ceramide synthase 1 (LASS1)	Cytokine	ND	ND	ND	ND	ND	ND	ND	ND
ILMN_1781256	LEFTY2	Homo sapiens left-right determination factor 2 (LEFTY2)	Cytokine	+	+	+	-	-	-	+	+
ILMN_1795464	LTA	Homo sapiens lymphotoxin alpha (TNF superfamily, member 1	Cytokine	-	+	+	+	+	+	+	+
ILMN_2376204	LTB	Homo sapiens lymphotoxin beta (TNF superfamily, member 3)	Cytokine	+	+	+	+	+	+	+	+
ILMN_2376205	LTB	Homo sapiens lymphotoxin beta (TNF superfamily, member 3)	Cytokine	+	-	+	++	++	++	++	++
ILMN_1780516	MSTN	Homo sapiens myostatin (MSTN)	Cytokine	ND	ND	ND	ND	ND	ND	ND	ND
ILMN_1712537	NODAL	Homo sapiens nodal homolog (mouse) (NODAL)	Cytokine	ND	ND	ND	ND	ND	ND	ND	ND
ILMN_2342695	PDGFA	Homo sapiens platelet-derived growth factor alpha polypeptide	Cytokine	+	+	+	+	+	+	+	+
ILMN_1782661	PDGFA	PREDICTED: Homo sapiens platelet-derived growth factor alp	Cytokine	+	+	+	+	+	+	+	+
ILMN_1685982	PDGFA	Homo sapiens platelet-derived growth factor alpha polypeptide	Cytokine	ND	ND	ND	ND	ND	ND	ND	ND
ILMN_1775822	PDGFB	Homo sapiens platelet-derived growth factor beta polypeptide (	Cytokine	+	+	+	+	+	+	+	+
ILMN_2394305	PDGFB	Homo sapiens platelet-derived growth factor beta polypeptide (	Cytokine	+	+	+	+	+	+	+	+
ILMN_1737695	PDGFB	Homo sapiens platelet-derived growth factor beta polypeptide (	Cytokine	ND	ND	ND	ND	ND	ND	ND	ND
ILMN_1813753	PTN	Homo sapiens pleiotrophin (PTN)	Cytokine	ND	ND	ND	ND	ND	ND	ND	ND
ILMN_1755883	RPS27A	Homo sapiens ribosomal protein S27a (RPS27A)	Cytokine	+	+	-	+	+	+	+	+
ILMN_2048326	RPS27A	Homo sapiens ribosomal protein S27a (RPS27A)	Cytokine	+++	+++	+++	+++	+++	+++	+++	+++
ILMN_1705080	SLURP1	Homo sapiens secreted LY6/PLAUR domain containing 1 (SLU	Cytokine	+	+	+	+	+	+	+	+
ILMN_2374449	SPP1	Homo sapiens secreted phosphoprotein 1 (SPP1)	Cytokine	+	+	+	+	+	+	+	+
ILMN_1651354	SPP1	Homo sapiens secreted phosphoprotein 1 (SPP1)	Cytokine	+	++	++	+	+	++	+++	+
ILMN_2083946	TGFA	Homo sapiens transforming growth factor, alpha (TGFA)	Cytokine	+	+	+	+	+	+	+	+
ILMN_1805175	TGFA	Homo sapiens transforming growth factor, alpha (TGFA)	Cytokine	+	+	+	+	+	+	+	+
ILMN_2129668	TGFB1	Homo sapiens transforming growth factor, beta 1 (TGFB1)	Cytokine	+	-	+	+	+	+	+	-
ILMN_1812526	TGFB2	Homo sapiens transforming growth factor, beta 2 (TGFB2)	Cytokine	ND	ND	ND	ND	ND	ND	ND	ND
ILMN_1687652	TGFB3	Homo sapiens transforming growth factor, beta 3 (TGFB3)	Cytokine	+	+	-	+	+	+	-	+
ILMN_1737943	THPO	Homo sapiens thrombopoietin (THPO)	Cytokine	ND	ND	ND	ND	ND	ND	ND	ND
ILMN_1728106	TNF	Homo sapiens tumor necrosis factor (TNF superfamily, membe	Cytokine	+	++	++	++	+	++	++	+
ILMN_1676663	TNFRSF11E	Homo sapiens tumor necrosis factor receptor superfamily, mem	Cytokine	ND	ND	ND	ND	ND	ND	ND	ND
ILMN_1801307	TNFSF10	Homo sapiens tumor necrosis factor (ligand) superfamily, mem	Cytokine	+	+	+	+	+	+	+	++
ILMN_1658713	TNFSF11	Homo sapiens tumor necrosis factor (ligand) superfamily, mem	Cytokine	+	+	+	+	+	+	+	+
ILMN_1673175	TNFSF11	Homo sapiens tumor necrosis factor (ligand) superfamily, mem	Cytokine	+	+	+	+	+	+	-	+
ILMN_1695900	TNFSF11	Homo sapiens tumor necrosis factor (ligand) superfamily, mem	Cytokine	ND	ND	ND	ND	ND	ND	ND	ND
ILMN_2399190	TNFSF13	Homo sapiens tumor necrosis factor (ligand) superfamily, mem	Cytokine	ND	ND	ND	ND	ND	ND	ND	ND
ILMN_1784264	TNFSF13	Homo sapiens tumor necrosis factor (ligand) superfamily, mem	Cytokine	ND	ND	ND	ND	ND	ND	ND	ND
ILMN_1758418	TNFSF13B	Homo sapiens tumor necrosis factor (ligand) superfamily, mem	Cytokine	+	+	+	+	+	+	+	+
ILMN_2066858	TNFSF13B	Homo sapiens tumor necrosis factor (ligand) superfamily, mem	Cytokine	+	++	++	++	++	++	++	++
ILMN_1655414	TNFSF14	Homo sapiens tumor necrosis factor (ligand) superfamily, mem	Cytokine	+	+	+	+	+	+	+	-
ILMN_2363392	TNFSF14	Homo sapiens tumor necrosis factor (ligand) superfamily, mem	Cytokine	++	++	++	++	++	++	++	++
ILMN_1661343	TNFSF14	Homo sapiens tumor necrosis factor (ligand) superfamily, mem	Cytokine	ND	ND	ND	ND	ND	ND	ND	ND
ILMN_1759501	TNFSF15	Homo sapiens tumor necrosis factor (ligand) superfamily, mem	Cytokine	+	+	+	+	+	+	+	+
ILMN_2106380	TNFSF15	Homo sapiens tumor necrosis factor (ligand) superfamily, mem	Cytokine	+	++	++	+	++	+	+	+
ILMN_1738335	TNFSF18	Homo sapiens tumor necrosis factor (ligand) superfamily, mem	Cytokine	ND	ND	ND	ND	ND	ND	ND	ND
ILMN_2089875	TNFSF4	Homo sapiens tumor necrosis factor (ligand) superfamily, mem	Cytokine	++	+	+	+	+	+	+	+
ILMN_1746175	TNFSF4	Homo sapiens tumor necrosis factor (ligand) superfamily, mem	Cytokine	++	+	+	+	+	+	+	+
ILMN_1761778	TNFSF8	Homo sapiens tumor necrosis factor (ligand) superfamily, mem	Cytokine	+	+	+	+	+	+	+	+
ILMN_1751464	TNFSF9	Homo sapiens tumor necrosis factor (ligand) superfamily, mem	Cytokine	+	+	+	+	+	+	+	+
ILMN_1737084	TXLNA	Homo sapiens taxilin alpha (TXLNA)	Cytokine	+	+	+	++	++	++	+	+
ILMN_1722855	VEGFB	Homo sapiens vascular endothelial growth factor B (VEGFB)	Cytokine	+	+	+	+	+	+	+	+
ILMN_1726981	VEGFB	Homo sapiens vascular endothelial growth factor B (VEGFB)	Cytokine	++	++	++	++	++	++	++	++
ILMN_1801814	VEGFB	Homo sapiens vascular endothelial growth factor B (VEGFB)	Cytokine	ND	ND	ND	ND	ND	ND	ND	ND
ILMN_1772274	VEGFB	PREDICTED: Homo sapiens vascular endothelial growth facto	Cytokine	ND	ND	ND	ND	ND	ND	ND	ND
ILMN_1798475	CSF2RB	Homo sapiens colony stimulating factor 2 receptor, beta, low-af	Interleukin Receptor	-	-	-	+	+	+	+	-
ILMN_1662524	CXCR1	Homo sapiens chemokine (C-X-C motif) receptor 1 (CXCR1)	Interleukin Receptor	-	-	+	+	+	+	+	-
ILMN_1652825	IL10RA	Homo sapiens interleukin 10 receptor, alpha (IL10RA)	Interleukin Receptor	++	++	++	++	++	++	++	++
ILMN_1767360	IL10RB	Homo sapiens interleukin 10 receptor, beta (IL10RB)	Interleukin Receptor	+	+	+	+	+	+	+	+
ILMN_2230892	IL10RB	Homo sapiens interleukin 10 receptor, beta (IL10RB)	Interleukin Receptor	++	++	+++	++	++	+++	+++	++

ILMN_1664912	IL11RA	Homo sapiens interleukin 11 receptor, alpha (IL11RA)	Interleukin Receptor	++	++	++	++	++	++	++	++
ILMN_1720024	IL11RA	Homo sapiens interleukin 11 receptor, alpha (IL11RA)	Interleukin Receptor	ND	ND	ND	ND	ND	ND	ND	ND
ILMN_1653459	IL11RA	Homo sapiens interleukin 11 receptor, alpha (IL11RA)	Interleukin Receptor	ND	ND	ND	ND	ND	ND	ND	ND
ILMN_1815890	IL12RB1	Homo sapiens interleukin 12 receptor, beta 1 (IL12RB1)	Interleukin Receptor	+	+	-	+	+	+	+	+
ILMN_1789645	IL12RB1	Homo sapiens interleukin 12 receptor, beta 1 (IL12RB1)	Interleukin Receptor	ND	ND	ND	ND	ND	ND	ND	ND
ILMN_1699908	IL12RB1	Homo sapiens interleukin 12 receptor, beta 1 (IL12RB1)	Interleukin Receptor	ND	ND	ND	ND	ND	ND	ND	ND
ILMN_1761921	IL12RB2	Homo sapiens interleukin 12 receptor, beta 2 (IL12RB2)	Interleukin Receptor	ND	ND	ND	ND	ND	ND	ND	ND
ILMN_1768505	IL13RA1	Homo sapiens interleukin 13 receptor, alpha 1 (IL13RA1)	Interleukin Receptor	+++	+++	+++	+++	+++	+++	+++	+++
ILMN_1688722	IL13RA2	Homo sapiens interleukin 13 receptor, alpha 2 (IL13RA2)	Interleukin Receptor	+	+	+	+	+	+	+	+
ILMN_1693910	IL15RA	Homo sapiens interleukin 15 receptor, alpha (IL15RA)	Interleukin Receptor	+	+	+	+	+	+	+	+
ILMN_1759958	IL15RA	Homo sapiens interleukin 15 receptor, alpha (IL15RA)	Interleukin Receptor	+	+	+	+	+	+	+	+
ILMN_1665682	IL15RA	Homo sapiens interleukin 15 receptor, alpha (IL15RA)	Interleukin Receptor	+	+	+	+	+	+	+	+
ILMN_1728724	IL17RA	Homo sapiens interleukin 17 receptor A (IL17RA)	Interleukin Receptor	+	+	+	+	+	+	+	+
ILMN_1767523	IL17RB	Homo sapiens interleukin 17 receptor B (IL17RB)	Interleukin Receptor	ND	ND	ND	ND	ND	ND	ND	ND
ILMN_1710747	IL17RB	Homo sapiens interleukin 17 receptor B (IL17RB)	Interleukin Receptor	ND	ND	ND	ND	ND	ND	ND	ND
ILMN_2369919	IL17RC	Homo sapiens interleukin 17 receptor C (IL17RC)	Interleukin Receptor	+	+	+	+	+	+	+	+
ILMN_1798790	IL17RC	Homo sapiens interleukin 17 receptor C (IL17RC)	Interleukin Receptor	+	+	+	-	+	+	+	+
ILMN_1689357	IL17RC	Homo sapiens interleukin 17 receptor C (IL17RC)	Interleukin Receptor	+	+	+	+	+	+	+	+
ILMN_1690458	IL17RC	Homo sapiens interleukin 17 receptor C (IL17RC)	Interleukin Receptor	ND	ND	ND	ND	ND	ND	ND	ND
ILMN_1666226	IL17RC	Homo sapiens interleukin 17 receptor C (IL17RC)	Interleukin Receptor	ND	ND	ND	ND	ND	ND	ND	ND
ILMN_2407851	IL17RD	Homo sapiens interleukin 17 receptor D (IL17RD)	Interleukin Receptor	++	++	++	++	+++	++	++	++
ILMN_2294542	IL17RD	Homo sapiens interleukin 17 receptor D (IL17RD)	Interleukin Receptor	ND	ND	ND	ND	ND	ND	ND	ND
ILMN_1728663	IL17RD	Homo sapiens interleukin 17 receptor D (IL17RD)	Interleukin Receptor	ND	ND	ND	ND	ND	ND	ND	ND
ILMN_2409876	IL17RE	Homo sapiens interleukin 17 receptor E (IL17RE)	Interleukin Receptor	ND	ND	ND	ND	ND	ND	ND	ND
ILMN_1752089	IL17RE	Homo sapiens interleukin 17 receptor E (IL17RE)	Interleukin Receptor	ND	ND	ND	ND	ND	ND	ND	ND
ILMN_1689718	IL17RE	Homo sapiens interleukin 17 receptor E (IL17RE)	Interleukin Receptor	ND	ND	ND	ND	ND	ND	ND	ND
ILMN_1781700	IL18R1	Homo sapiens interleukin 18 receptor 1 (IL18R1)	Interleukin Receptor	+	++	++	+	+	+	+	+
ILMN_1721762	IL18RAP	Homo sapiens interleukin 18 receptor accessory protein (IL18R	Interleukin Receptor	+	+	+	+	+	+	+	+
ILMN_1810584	IL1R1	Homo sapiens interleukin 1 receptor, type I (IL1R1)	Interleukin Receptor	++	++	++	++	++	+	+	-
ILMN_1758371	IL1R2	Homo sapiens interleukin 1 receptor, type II (IL1R2)	Interleukin Receptor	+	+	+	+	+	+	-	+
ILMN_1772131	IL1R2	Homo sapiens interleukin 1 receptor, type II (IL1R2)	Interleukin Receptor	++	+++	+++	++	++	++	+++	++
ILMN_2251766	IL1R2	Homo sapiens interleukin 1 receptor, type II (IL1R2)	Interleukin Receptor	ND	ND	ND	ND	ND	ND	ND	ND
ILMN_2357062	IL1RAP	Homo sapiens interleukin 1 receptor accessory protein (IL1RAI	Interleukin Receptor	-	-	-	+	+	+	+	-
ILMN_1686884	IL1RAP	Homo sapiens interleukin 1 receptor accessory protein (IL1RAI	Interleukin Receptor	+	+	+	+	+	+	+	+
ILMN_1793870	IL1RAP	Homo sapiens interleukin 1 receptor accessory protein (IL1RAI	Interleukin Receptor	ND	ND	ND	ND	ND	ND	ND	ND
ILMN_2160428	IL1RAPL1	Homo sapiens interleukin 1 receptor accessory protein-like 1 (I	Interleukin Receptor	ND	ND	ND	ND	ND	ND	ND	ND
ILMN_1767413	IL1RAPL2	Homo sapiens interleukin 1 receptor accessory protein-like 2 (I	Interleukin Receptor	-	-	-	-	-	-	-	+
ILMN_1695107	IL20RA	Homo sapiens interleukin 20 receptor, alpha (IL20RA)	Interleukin Receptor	ND	ND	ND	ND	ND	ND	ND	ND
ILMN_1722209	IL21R	Homo sapiens interleukin 21 receptor (IL21R)	Interleukin Receptor	-	-	-	-	-	+	-	+
ILMN_1798204	IL21R	Homo sapiens interleukin 21 receptor (IL21R)	Interleukin Receptor	+	+	+	+	+	+	+	-
ILMN_1661687	IL21R	Homo sapiens interleukin 21 receptor (IL21R)	Interleukin Receptor	+	+	+	+	+	+	+	+
ILMN_1786609	IL21R	Homo sapiens interleukin 21 receptor (IL21R)	Interleukin Receptor	ND	ND	ND	ND	ND	ND	ND	ND
ILMN_1666175	IL22RA1	Homo sapiens interleukin 22 receptor, alpha 1 (IL22RA1)	Interleukin Receptor	+	+	+	+	+	+	+	+
ILMN_2307721	IL22RA2	Homo sapiens interleukin 22 receptor, alpha 2 (IL22RA2)	Interleukin Receptor	ND	ND	ND	ND	ND	ND	ND	ND
ILMN_1805385	IL22RA2	Homo sapiens interleukin 22 receptor, alpha 2 (IL22RA2)	Interleukin Receptor	ND	ND	ND	ND	ND	ND	ND	ND
ILMN_1653324	IL22RA2	Homo sapiens interleukin 22 receptor, alpha 2 (IL22RA2)	Interleukin Receptor	ND	ND	ND	ND	ND	ND	ND	ND
ILMN_2223663	IL23R	Homo sapiens interleukin 23 receptor (IL23R)	Interleukin Receptor	ND	ND	ND	ND	ND	ND	ND	ND
ILMN_1734937	IL23R	Homo sapiens interleukin 23 receptor (IL23R)	Interleukin Receptor	ND	ND	ND	ND	ND	ND	ND	ND
ILMN_1688152	IL27RA	Homo sapiens interleukin 27 receptor, alpha (IL27RA)	Interleukin Receptor	-	-	+	+	+	+	+	+
ILMN_1680805	IL28RA	Homo sapiens interleukin 28 receptor, alpha (interferon, lambda	Interleukin Receptor	+	+	+	+	+	+	+	+
ILMN_2405324	IL28RA	Homo sapiens interleukin 28 receptor, alpha (interferon, lambda	Interleukin Receptor	+	+	+	+	+	+	+	+
ILMN_2108699	IL2RA	Homo sapiens interleukin 2 receptor, alpha (IL2RA)	Interleukin Receptor	+	+	+	+	+	+	+	+
ILMN_1683774	IL2RA	Homo sapiens interleukin 2 receptor, alpha (IL2RA)	Interleukin Receptor	+	++	++	+	+	+	+	+
ILMN_1684349	IL2RB	Homo sapiens interleukin 2 receptor, beta (IL2RB)	Interleukin Receptor	+	+	+	+	+	+	+	+
ILMN_1667575	IL31RA	Homo sapiens interleukin 31 receptor A (IL31RA)	Interleukin Receptor	+	+	+	+	+	+	+	+
ILMN_1747344	IL3RA	Homo sapiens interleukin 3 receptor, alpha (low affinity) (IL3R	Interleukin Receptor	+	++	++	+	+	++	++	+
ILMN_1691881	IL4R	Homo sapiens interleukin 4 receptor (IL4R)	Interleukin Receptor	+	-	+	+	+	+	+	+
ILMN_1652185	IL4R	Homo sapiens interleukin 4 receptor (IL4R)	Interleukin Receptor	++	++	++	++	++	++	++	++
ILMN_2327812	IL5RA	Homo sapiens interleukin 5 receptor, alpha (IL5RA)	Interleukin Receptor	+	+	+	-	+	-	+	+
ILMN_1756455	IL5RA	Homo sapiens interleukin 5 receptor, alpha (IL5RA)	Interleukin Receptor	+	+	+	+	+	+	+	+
ILMN_1754753	IL6R	Homo sapiens interleukin 6 receptor (IL6R)	Interleukin Receptor	-	-	+	+	+	+	+	-
ILMN_1696394	IL6R	Homo sapiens interleukin 6 receptor (IL6R)	Interleukin Receptor	-	-	+	+	+	+	+	-
ILMN_1746604	IL6ST	Homo sapiens interleukin 6 signal transducer (gp130, oncostati	Interleukin Receptor	-	-	+	+	+	+	+	-
ILMN_1797861	IL6ST	Homo sapiens interleukin 6 signal transducer (gp130, oncostati	Interleukin Receptor	ND	ND	ND	ND	ND	ND	ND	ND
ILMN_2342579	IL7R	Homo sapiens interleukin 7 receptor (IL7R)	Interleukin Receptor	+	+	+	+	+	+	+	+
ILMN_1691341	IL7R	PREDICTED: Homo sapiens interleukin 7 receptor (IL7R)	Interleukin Receptor	++	++	+++	+++	+++	+++	+++	+
ILMN_1680397	IL8RB	Homo sapiens interleukin 8 receptor, beta (IL8RB)	Interleukin Receptor	+	+	+	+	+	+	+	+
ILMN_1794686	IL9R	Homo sapiens interleukin 9 receptor (IL9R)	Interleukin Receptor	ND	ND	ND	ND	ND	ND	ND	ND
ILMN_1777296	ACTB	Homo sapiens actin, beta (ACTB)	Type I IFN Response	++	+	+	+	++	+	+	++
ILMN_2038777	ACTB	Homo sapiens actin, beta (ACTB)	Type I IFN Response	+++	+++	+++	+++	+++	+++	+++	+++
ILMN_2152131	ACTB	Homo sapiens actin, beta (ACTB)	Type I IFN Response	+++	+++	+++	+++	+++	+++	+++	+++
ILMN_1669113	ATF5	Homo sapiens activating transcription factor 5 (ATF5)	Type I IFN Response	+++	+++	+++	+++	+++	+++	+++	++
ILMN_2148459	B2M	Homo sapiens beta-2-microglobulin (B2M)	Type I IFN Response	+++	+++	+++	+++	+++	+++	+++	+++
ILMN_1725427	B2M	Homo sapiens beta-2-microglobulin (B2M)	Type I IFN Response	+++	+++	+++	+++	+++	+++	+++	+++
ILMN_1659766	BAG3	Homo sapiens BCL2-associated athanogene 3 (BAG3)	Type I IFN Response	++	++	+	++	++	++	++	++
ILMN_1723480	BST2	Homo sapiens bone marrow stromal cell antigen 2 (BST2)	Type I IFN Response	++	++	++	++	++	++	++	++
ILMN_1687583	CAV1	Homo sapiens caveolin 1, caveolae protein, 22kDa (CAV1)	Type I IFN Response	-	-	-	+	+	+	+	+
ILMN_2149226	CAV1	Homo sapiens caveolin 1, caveolae protein, 22kDa (CAV1)	Type I IFN Response	+	-	+	+	+	+	+	-
ILMN_1766408	CBFB	Homo sapiens core-binding factor, beta subunit (CBFB)	Type I IFN Response	+	++	+	+	+	+	+	+
ILMN_2399489	CBFB	Homo sapiens core-binding factor, beta subunit (CBFB)	Type I IFN Response	ND	ND	ND	ND	ND	ND	ND	ND
ILMN_1760247	CD70	Homo sapiens CD70 molecule (CD70)	Type I IFN Response	+	+	+	+	+	+	+	+
ILMN_1760374	CD8A	Homo sapiens CD8a molecule (CD8A)	Type I IFN Response	+	+	+	+	+	+	+	+
ILMN_2353732	CD8A	Homo sapiens CD8a molecule (CD8A)	Type I IFN Response	+	+	+	+	+	+	+	+
ILMN_1768482	CD8A	Homo sapiens CD8a molecule (CD8A)	Type I IFN Response	+	-	-	+	+	+	+	-
ILMN_1722811	CDKN1B	Homo sapiens cyclin-dependent kinase inhibitor 1B (p27, Kip1	Type I IFN Response	+	+	+	+	+	+	+	+
ILMN_2196347	CDKN1B	Homo sapiens cyclin-dependent kinase inhibitor 1B (p27, Kip1	Type I IFN Response	+	+	+	+	+	+	+	+
ILMN_1684554	COL16A1	Homo sapiens collagen, type XVI, alpha 1 (COL16A1)	Type I IFN Response	+	+	+	+	+	+	+	+
ILMN_1721204	CSF2RA	PREDICTED: Homo sapiens colony stimulating factor 2 recept	Type I IFN Response	++	++	++	++	+++	++	++	++
ILMN_1661196	CSF2RA	Homo sapiens colony stimulating factor 2 receptor, alpha, low-a	Type I IFN Response	+++	+++	+++	++	+++	++	++	++
ILMN_2376455	CSF2RA	Homo sapiens colony stimulating factor 2 receptor, alpha, low-a	Type I IFN Response	+++	+++	+++	++	+++	++	++	++
ILMN_2376458	CSF2RA	Homo sapiens colony stimulating factor 2 receptor, alpha, low-a	Type I IFN Response	+++	+++	+++	+++	+++	+++	+++	+++
ILMN_2371280	CSF3R	Homo sapiens colony stimulating factor 3 receptor (granulocyte	Type I IFN Response	+	+	+	+	+	+	++	++
ILMN_2323172	CSF3R	Homo sapiens colony stimulating factor 3 receptor (granulocyte	Type I IFN Response	ND	ND	ND	ND	ND	ND	ND	ND
ILMN_1791759	CXCL10	Homo sapiens chemokine (C-X-C motif) ligand 10 (CXCL10)	Type I IFN Response	-	-	-	-	-	+	+	++
ILMN_1731619	DAD1	Homo sapiens defender against cell death 1 (DAD1)	Type I IFN Response	++	++	++	++	++	++	++	++
ILMN_2285213	DNAJB2	Homo sapiens DnaJ (Hsp40) homolog, subfamily B, member 2	Type I IFN Response	+	+	+	+	+	+	+	+
ILMN_2390974	DNAJB2	Homo sapiens DnaJ (Hsp40) homolog, subfamily B, member 2	Type I IFN Response	+++	+++	+++	+++	+++	+++	+++	++
ILMN_1802252	GAPDH	Homo sapiens glyceraldehyde-3-phosphate dehydrogenase (GA	Type I IFN Response	++	++	+++	+++	+++	+++	+++	+++
ILMN_1343295	GAPDH	Homo sapiens glyceraldehyde-3-phosphate dehydrogenase (GA	Type I IFN Response	++	++	+++	+++	+++	+++	+++	+++
ILMN_2038778	GAPDH	Homo sapiens glyceraldehyde-3-phosphate dehydrogenase (GA	Type I IFN Response	+++	+++	+++	+++	+++	+++	+++	+++
ILMN_2148785	GBP1	Homo sapiens guanylate binding protein 1, interferon-inducible	Type I IFN Response	+	+	+	+	+	+	+	++
ILMN_1701114	GBP1	Homo sapiens guanylate binding protein 1, interferon-inducible	Type I IFN Response	+	+	+	+	+	+	+	++
ILMN_1774077	GBP2	Homo sapiens guanylate binding protein 2, interferon-inducible	Type I IFN Response	++	++	++	+++	++	++	++	+++
ILMN_1788802	GCH1	Homo sapiens GTP cyclohydrolase 1 (GCH1)	Type I IFN Response	-	-	-	+	+	+	+	-
ILMN_2335813	GCH1	Homo sapiens GTP cyclohydrolase 1 (GCH1)	Type I IFN Response	+	+	+	+	+	+	+	+

ILMN_1812759	GCH1	Homo sapiens GTP cyclohydrolase 1 (GCH1)	Type I IFN Response	+	+	+	+	-	+	+	+
ILMN_1671054	HLA-A	Homo sapiens major histocompatibility complex, class I, A (HL	Type I IFN Response	+++	+++	+++	+++	+++	+++	+++	+++
ILMN_2203950	HLA-A	Homo sapiens major histocompatibility complex, class I, A (HL	Type I IFN Response	+++	+++	+++	+++	+++	+++	+++	+++
ILMN_1810274	HOXB2	Homo sapiens homeobox B2 (HOXB2)	Type I IFN Response	+	+	+	+	+	+	+	+
ILMN_1673711	HSP90AB1	Homo sapiens heat shock protein 90kDa alpha (cytosolic), class	Type I IFN Response	++	++	++	++	++	++	++	++
ILMN_1658383	HSPA6	Homo sapiens heat shock 70kDa protein 6 (HSP70B') (HSPA6)	Type I IFN Response	-	-	-	+	+	+	+	-
ILMN_1806165	HSPA6	Homo sapiens heat shock 70kDa protein 6 (HSP70B') (HSPA6)	Type I IFN Response	+	++	++	+++	+++	+++	+++	++
ILMN_1710937	IFI16	Homo sapiens interferon, gamma-inducible protein 16 (IFI16)	Type I IFN Response	+	+	+	+	+	+	+	+
ILMN_2058782	IFI27	Homo sapiens interferon, alpha-inducible protein 27 (IFI27)	Type I IFN Response	+	+	+	++	++	+	+	+
ILMN_1807277	IFI30	Homo sapiens interferon, gamma-inducible protein 30 (IFI30)	Type I IFN Response	+++	++	+++	+++	+++	+++	+++	+++
ILMN_1745374	IFI35	Homo sapiens interferon-induced protein 35 (IFI35)	Type I IFN Response	+	+	+	+	+	+	+	+
ILMN_1760062	IFI44	Homo sapiens interferon-induced protein 44 (IFI44)	Type I IFN Response	+	+	+	+	+	+	++	+++
ILMN_1723912	IFI44L	Homo sapiens interferon-induced protein 44-like (IFI44L)	Type I IFN Response	+	+	+	+	+	+	+	++
ILMN_2347798	IFI6	Homo sapiens interferon, alpha-inducible protein 6 (IFI6)	Type I IFN Response	+	+	+	+	+	+	+	+
ILMN_1687384	IFI6	Homo sapiens interferon, alpha-inducible protein 6 (IFI6)	Type I IFN Response	+	+	+	+	+	++	++	++
ILMN_1781373	IFIH1	Homo sapiens interferon induced with helicase C domain 1 (IFI	Type I IFN Response	++	+	+	++	++	+	++	++
ILMN_1739428	IFIT2	Homo sapiens interferon-induced protein with tetratricopeptide	Type I IFN Response	++	++	++	++	++	++	++	+++
ILMN_1696654	IFIT5	Homo sapiens interferon-induced protein with tetratricopeptide	Type I IFN Response	+	+	+	+	+	+	+	+
ILMN_1801246	IFITM1	Homo sapiens interferon induced transmembrane protein 1 (9-2	Type I IFN Response	+	+	+	+	+	+	+	+++
ILMN_1673352	IFITM2	Homo sapiens interferon induced transmembrane protein 2 (1-8	Type I IFN Response	++	++	++	++	++	++	++	+++
ILMN_1688663	IFNA1	Homo sapiens interferon, alpha 1 (IFNA1)	Type I IFN Response	ND	ND	ND	ND	ND	ND	ND	ND
ILMN_1698186	IFNA2	Homo sapiens interferon, alpha 2 (IFNA2)	Type I IFN Response	ND	ND	ND	ND	ND	ND	ND	ND
ILMN_1782448	IFNA4	Homo sapiens interferon, alpha 4 (IFNA4)	Type I IFN Response	+	+	+	+	+	+	+	+
ILMN_2202096	IFNA4	Homo sapiens interferon, alpha 4 (IFNA4)	Type I IFN Response	ND	ND	ND	ND	ND	ND	ND	ND
ILMN_1752923	IFNAR1	Homo sapiens interferon (alpha, beta and omega) receptor 1 (IF	Type I IFN Response	+	+	+	++	++	++	++	+
ILMN_1791057	IFNAR2	Homo sapiens interferon (alpha, beta and omega) receptor 2 (IF	Type I IFN Response	-	+	+	+	+	+	+	+
ILMN_1765146	IFNAR2	Homo sapiens interferon (alpha, beta and omega) receptor 2 (IF	Type I IFN Response	+	+	+	+	+	+	++	++
ILMN_2347999	IFNAR2	Homo sapiens interferon (alpha, beta and omega) receptor 2 (IF	Type I IFN Response	ND	ND	ND	ND	ND	ND	ND	ND
ILMN_1682245	IFNB1	Homo sapiens interferon, beta 1, fibroblast (IFNB1)	Type I IFN Response	ND	ND	ND	ND	ND	ND	ND	ND
ILMN_2207291	IFNG	Homo sapiens interferon, gamma (IFNG)	Type I IFN Response	+	-	+	+	+	+	+	+
ILMN_1675939	IFNGR1	Homo sapiens interferon gamma receptor 1 (IFNGR1)	Type I IFN Response	++	+++	+++	+++	+++	+++	+++	+++
ILMN_1764964	IFNGR2	Homo sapiens interferon gamma receptor 2 (interferon gamma	Type I IFN Response	+++	+++	+++	+++	+++	+++	+++	+++
ILMN_1781057	IFNW1	Homo sapiens interferon, omega 1 (IFNW1)	Type I IFN Response	+	+	+	+	+	+	+	+
ILMN_2176225	IFNW1	Homo sapiens interferon, omega 1 (IFNW1)	Type I IFN Response	ND	ND	ND	ND	ND	ND	ND	ND
ILMN_1742031	IFRD2	Homo sapiens interferon-related developmental regulator 2 (IFI	Type I IFN Response	+	-	+	+	+	+	+	+
ILMN_1652825	IL10RA	Homo sapiens interleukin 10 receptor, alpha (IL10RA)	Type I IFN Response	++	++	++	++	++	++	++	++
ILMN_1664912	IL11RA	Homo sapiens interleukin 11 receptor, alpha (IL11RA)	Type I IFN Response	++	++	++	++	++	++	++	++
ILMN_1720024	IL11RA	Homo sapiens interleukin 11 receptor, alpha (IL11RA)	Type I IFN Response	ND	ND	ND	ND	ND	ND	ND	ND
ILMN_1653459	IL11RA	Homo sapiens interleukin 11 receptor, alpha (IL11RA)	Type I IFN Response	ND	ND	ND	ND	ND	ND	ND	ND
ILMN_1681132	IL12B	Homo sapiens interleukin 12B (natural killer cell stimulatory fa	Type I IFN Response	ND	ND	ND	ND	ND	ND	ND	ND
ILMN_1768505	IL13RA1	Homo sapiens interleukin 13 receptor, alpha 1 (IL13RA1)	Type I IFN Response	+++	+++	+++	+++	+++	+++	+++	+++
ILMN_1684349	IL2RB	Homo sapiens interleukin 2 receptor, beta (IL2RB)	Type I IFN Response	+	+	+	+	+	+	+	+
ILMN_1794386	IL2RG	Homo sapiens interleukin 2 receptor, gamma (severe combined	Type I IFN Response	+	+	+	-	+	+	+	+
ILMN_1747344	IL3RA	Homo sapiens interleukin 3 receptor, alpha (low affinity) (IL3R	Type I IFN Response	+	++	++	+	+	++	++	+
ILMN_1691881	IL4R	Homo sapiens interleukin 4 receptor (IL4R)	Type I IFN Response	+	-	+	+	+	+	+	+
ILMN_1652185	IL4R	Homo sapiens interleukin 4 receptor (IL4R)	Type I IFN Response	++	++	++	++	++	++	++	++
ILMN_2327812	IL5RA	Homo sapiens interleukin 5 receptor, alpha (IL5RA)	Type I IFN Response	+	+	+	-	+	-	+	+
ILMN_1756455	IL5RA	Homo sapiens interleukin 5 receptor, alpha (IL5RA)	Type I IFN Response	+	+	+	+	+	+	+	+
ILMN_1754753	IL6R	Homo sapiens interleukin 6 receptor (IL6R)	Type I IFN Response	-	-	+	+	+	+	+	-
ILMN_1696394	IL6R	Homo sapiens interleukin 6 receptor (IL6R)	Type I IFN Response	-	-	+	+	+	+	+	-
ILMN_2342579	IL7R	Homo sapiens interleukin 7 receptor (IL7R)	Type I IFN Response	+	+	+	+	+	+	+	+
ILMN_1691341	IL7R	PREDICTED: Homo sapiens interleukin 7 receptor (IL7R)	Type I IFN Response	++	++	+++	+++	+++	+++	+++	+
ILMN_1794686	IL9R	Homo sapiens interleukin 9 receptor (IL9R)	Type I IFN Response	ND	ND	ND	ND	ND	ND	ND	ND
ILMN_1708375	IRF1	Homo sapiens interferon regulatory factor 1 (IRF1)	Type I IFN Response	+	++	++	++	++	++	++	++
ILMN_1765547	IRF2	Homo sapiens interferon regulatory factor 2 (IRF2)	Type I IFN Response	+	+	+	+	+	+	+	+
ILMN_2090607	IRF2	Homo sapiens interferon regulatory factor 2 (IRF2)	Type I IFN Response	+	+	+	-	-	-	+	-
ILMN_1765649	IRF3	Homo sapiens interferon regulatory factor 3 (IRF3)	Type I IFN Response	+	+	+	+	+	+	+	+
ILMN_1670576	IRF5	Homo sapiens interferon regulatory factor 5 (IRF5)	Type I IFN Response	+	+	+	+	+	+	+	+
ILMN_2312606	IRF5	Homo sapiens interferon regulatory factor 5 (IRF5)	Type I IFN Response	++	++	++	++	++	++	++	++
ILMN_2349061	IRF7	Homo sapiens interferon regulatory factor 7 (IRF7)	Type I IFN Response	+	+	+	+	+	+	+	+
ILMN_1798181	IRF7	Homo sapiens interferon regulatory factor 7 (IRF7)	Type I IFN Response	++	++	++	++	++	++	++	+++
ILMN_1674646	IRF7	Homo sapiens interferon regulatory factor 7 (IRF7)	Type I IFN Response	ND	ND	ND	ND	ND	ND	ND	ND
ILMN_2054019	ISG15	Homo sapiens ISG15 ubiquitin-like modifier (ISG15)	Type I IFN Response	++	++	++	+++	++	++	++	+++
ILMN_1659913	ISG20	Homo sapiens interferon stimulated exonuclease gene 20kDa (I	Type I IFN Response	+++	+++	+++	+++	+++	+++	++	+++
ILMN_1808494	ITIH2	Homo sapiens inter-alpha (globulin) inhibitor H2 (ITIH2)	Type I IFN Response	+	+	+	+	+	+	+	+
ILMN_1793384	JAK1	Homo sapiens Janus kinase 1 (JAK1)	Type I IFN Response	+	+	+	+	+	+	+	+
ILMN_1782292	LAMP1	Homo sapiens lysosomal-associated membrane protein 1 (LAM	Type I IFN Response	+++	+++	+++	+++	+++	+++	+++	++
ILMN_2320330	MAL	Homo sapiens mal, T-cell differentiation protein (MAL)	Type I IFN Response	+	+	+	++	++	+	++	+
ILMN_2327860	MAL	Homo sapiens mal, T-cell differentiation protein (MAL)	Type I IFN Response	++	++	+	+++	+++	+++	+++	++
ILMN_1694240	MAP2K1	Homo sapiens mitogen-activated protein kinase kinase 1 (MAP	Type I IFN Response	++	+++	++	++	++	++	++	+++
ILMN_1715175	MET	Homo sapiens met proto-oncogene (hepatocyte growth factor re	Type I IFN Response	+	+	++	+	+	+	+	+
ILMN_1651767	MKL1	Homo sapiens megakaryoblastic leukemia (translocation) 1 (MI	Type I IFN Response	+	+	+	+	+	+	+	+
ILMN_1792910	MNT	Homo sapiens MAX binding protein (MNT)	Type I IFN Response	+	+	+	+	+	+	+	+
ILMN_1662358	MX1	Homo sapiens myxovirus (influenza virus) resistance 1, interfer	Type I IFN Response	+++	++	++	+++	++	+++	+++	+++
ILMN_2231928	MX2	Homo sapiens myxovirus (influenza virus) resistance 2 (mouse	Type I IFN Response	+	+	+	+	+	+	+	++
ILMN_2110908	MYC	Homo sapiens v-myc myelocytomatosis viral oncogene homolo	Type I IFN Response	-	-	+	+	+	+	+	-
ILMN_1680618	MYC	Homo sapiens v-myc myelocytomatosis viral oncogene homolo	Type I IFN Response	+	+	+	+	+	+	+	+
ILMN_1738523	MYD88	Homo sapiens myeloid differentiation primary response gene (8	Type I IFN Response	+	+	+	+	+	+	+	++
ILMN_1812616	MYO1C	Homo sapiens myosin IC (MYO1C)	Type I IFN Response	+	+	+	+	+	+	+	-
ILMN_2329165	MYO1C	Homo sapiens myosin IC (MYO1C)	Type I IFN Response	+	+	+	-	+	+	+	+
ILMN_2250820	MYO1C	Homo sapiens myosin IC (MYO1C)	Type I IFN Response	ND	ND	ND	ND	ND	ND	ND	ND
ILMN_1739541	NMI	Homo sapiens N-myc (and STAT) interactor (NMI)	Type I IFN Response	+	+	+	+	+	+	+	+
ILMN_2059797	NOL3	Homo sapiens nucleolar protein 3 (apoptosis repressor with CA	Type I IFN Response	+	+	+	+	+	+	-	+
ILMN_1813925	NOL3	Homo sapiens nucleolar protein 3 (apoptosis repressor with CA	Type I IFN Response	+	+	+	+	+	+	+	+
ILMN_1681721	OASL	Homo sapiens 2'-5'-oligoadenylate synthetase-like (OASL)	Type I IFN Response	+	+	+	+	+	+	+	+
ILMN_1674811	OASL	Homo sapiens 2'-5'-oligoadenylate synthetase-like (OASL)	Type I IFN Response	++	++	+	++	+	++	++	++
ILMN_1721549	PLA2G1B	Homo sapiens phospholipase A2, group IB (pancreas) (PLA2G	Type I IFN Response	ND	ND	ND	ND	ND	ND	ND	ND
ILMN_1758474	PRKRA	Homo sapiens protein kinase, interferon-inducible double stran	Type I IFN Response	+	+	+	+	+	+	+	+
ILMN_1804490	PRKRIP1	Homo sapiens PRKR interacting protein 1 (IL11 inducible) (PR	Type I IFN Response	+	+	+	+	+	+	+	+
ILMN_1786612	PSME2	Homo sapiens proteasome (prosome, macropain) activator subu	Type I IFN Response	++	++	+	++	++	++	+	++
ILMN_1753196	PTTG1	Homo sapiens pituitary tumor-transforming 1 (PTTG1)	Type I IFN Response	+	+	+	+	+	+	+	+
ILMN_2042771	PTTG1	Homo sapiens pituitary tumor-transforming 1 (PTTG1)	Type I IFN Response	+	++	+	+	+	+	+	+
ILMN_1695317	RCBTB1	Homo sapiens regulator of chromosome condensation (RCC1) a	Type I IFN Response	+	-	-	+	+	+	+	+
ILMN_1769637	RNMT	Homo sapiens RNA (guanine-7-) methyltransferase (RNMT)	Type I IFN Response	+	+	+	+	+	+	+	+
ILMN_1755883	RPS27A	Homo sapiens ribosomal protein S27a (RPS27A)	Type I IFN Response	+	+	-	+	+	+	+	+
ILMN_2048326	RPS27A	Homo sapiens ribosomal protein S27a (RPS27A)	Type I IFN Response	+++	+++	+++	+++	+++	+++	+++	+++
ILMN_2171289	SAMSN1	Homo sapiens SAM domain, SH3 domain and nuclear localizat	Type I IFN Response	+	+	+	+	+	+	+	+
ILMN_1684887	SAMSN1	Homo sapiens SAM domain, SH3 domain and nuclear localizat	Type I IFN Response	++	++	++	++	++	++	++	++
ILMN_1684211	SEC14L2	Homo sapiens SEC14-like 2 (S. cerevisiae) (SEC14L2)	Type I IFN Response	+	+	+	+	+	+	+	+
ILMN_1705892	SH2D1A	Homo sapiens SH2 domain protein 1A, Duncan's disease (lymp	Type I IFN Response	+	+	+	+	+	+	+	+
ILMN_1785380	SLC1A2	Homo sapiens solute carrier family 1 (glial high affinity glutam	Type I IFN Response	+	+	+	+	+	+	+	-
ILMN_1742224	SLTM	Homo sapiens SAFB-like, transcription modulator (SLTM)	Type I IFN Response	++	++	++	+	+	+	++	++
ILMN_2395204	SLTM	Homo sapiens SAFB-like, transcription modulator (SLTM)	Type I IFN Response	++	++	++	++	++	++	++	++
ILMN_1718815	SLTM	Homo sapiens SAFB-like, transcription modulator (SLTM)	Type I IFN Response	ND	ND	ND	ND	ND	ND	ND	ND
ILMN_1774733	SOCS1	Homo sapiens suppressor of cytokine signaling 1 (SOCS1)	Type I IFN Response	+	+	+	+	+	+	+	-

ILMN_2156250	SOCS3	Homo sapiens suppressor of cytokine signaling 3 (SOCS3)	Type I IFN Response	+	+	+	+	+	+	+	+
ILMN_1781001	SOCS3	Homo sapiens suppressor of cytokine signaling 3 (SOCS3)	Type I IFN Response	+	+	+	+	+	+	+	+
ILMN_1690105	STAT1	Homo sapiens signal transducer and activator of transcription 1	Type I IFN Response	+	+	+	+	+	+	+	+
ILMN_1777325	STAT1	Homo sapiens signal transducer and activator of transcription 1	Type I IFN Response	+	+	+	+	+	++	++	++
ILMN_1691364	STAT1	Homo sapiens signal transducer and activator of transcription 1	Type I IFN Response	++	++	++	++	++	+++	+++	+++
ILMN_1690921	STAT2	Homo sapiens signal transducer and activator of transcription 2	Type I IFN Response	++	++	+++	+++	++	+++	+++	+++
ILMN_1751079	TAP1	Homo sapiens transporter 1, ATP-binding cassette, sub-family I	Type I IFN Response	+++	+++	+++	+++	+++	+++	+++	+++
ILMN_1773031	TAPBP	Homo sapiens TAP binding protein (tapasin) (TAPBP)	Type I IFN Response	-	-	-	+	+	+	+	-
ILMN_1742450	TAPBP	Homo sapiens TAP binding protein (tapasin) (TAPBP)	Type I IFN Response	++	++	+	++	++	++	++	++
ILMN_1782851	TAPBP	Homo sapiens TAP binding protein (tapasin) (TAPBP)	Type I IFN Response	ND	ND	ND	ND	ND	ND	ND	ND
ILMN_1812432	TIRAP	Homo sapiens toll-interleukin 1 receptor (TIR) domain containi	Type I IFN Response	+	+	+	+	+	+	+	+
ILMN_2290679	TIRAP	Homo sapiens toll-interleukin 1 receptor (TIR) domain containi	Type I IFN Response	+	+	+	+	+	+	+	+
ILMN_1776703	TIRAP	Homo sapiens toll-interleukin 1 receptor (TIR) domain containi	Type I IFN Response	+	+	+	+	+	+	+	+
ILMN_2400603	TIRAP	Homo sapiens toll-interleukin 1 receptor (TIR) domain containi	Type I IFN Response	ND	ND	ND	ND	ND	ND	ND	ND
ILMN_1801307	TNFSF10	Homo sapiens tumor necrosis factor (ligand) superfamily, mem	Type I IFN Response	+	+	+	+	+	+	+	++
ILMN_1779252	TRIM22	Homo sapiens tripartite motif-containing 22 (TRIM22)	Type I IFN Response	-	+	+	-	-	-	-	++
ILMN_1676955	TYK2	Homo sapiens tyrosine kinase 2 (TYK2)	Type I IFN Response	+++	++	++	++	++	++	++	+++
