# Supplementary material for: Transcriptional fingerprints of antigen-presenting cell subsets in the human vaginal mucosa and skin reflect tissue-specific immune microenvironments
Source: Genome Med. 2014 Nov 25;6(11):98. doi: 10.1186/s13073-014-0098-y (PMC4268898; doi:10.1186/s13073-014-0098-y)
Supplement: Additional file 7: Figure S4. — Results of the comparisons between vaginal LC and macrophages. [file 13073_2014_98_MOESM7_ESM.pdf]

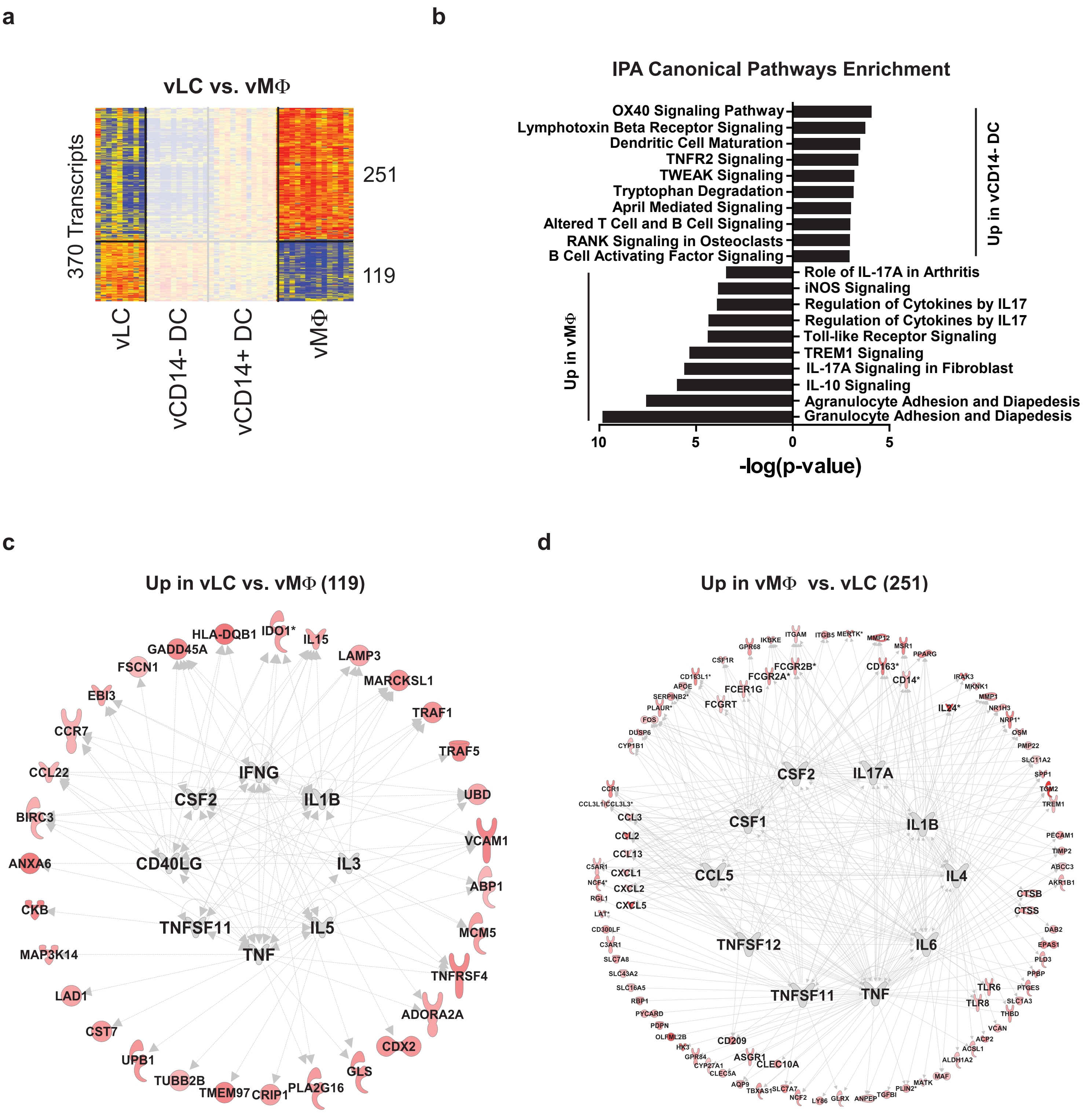

Figure S4: Transcriptional analysis of vLC vs. vMΦ. a. Hierarchical clustering of the 370 transcripts (identified by post-hoc Tukey test) differentially expressed between vLC and vMΦ. **b.** Bar chart representing the IPA canonical pathway enrichment for the 119 transcripts over-expressed in vLC and the 251 transcripts over-expressed vMF. **c.** Network analysis of the 119 transcripts over-expressed in vLC. **d.** Network analysis of the 251 transcripts over-expressed in vMΦ.
