# Supplementary material for: Transcriptional fingerprints of antigen-presenting cell subsets in the human vaginal mucosa and skin reflect tissue-specific immune microenvironments
Source: Genome Med. 2014 Nov 25;6(11):98. doi: 10.1186/s13073-014-0098-y (PMC4268898; doi:10.1186/s13073-014-0098-y)
Supplement: Additional file 14: Figure S11. — IPA analysis of DETs in skin and vagina. [file 13073_2014_98_MOESM14_ESM.pdf]

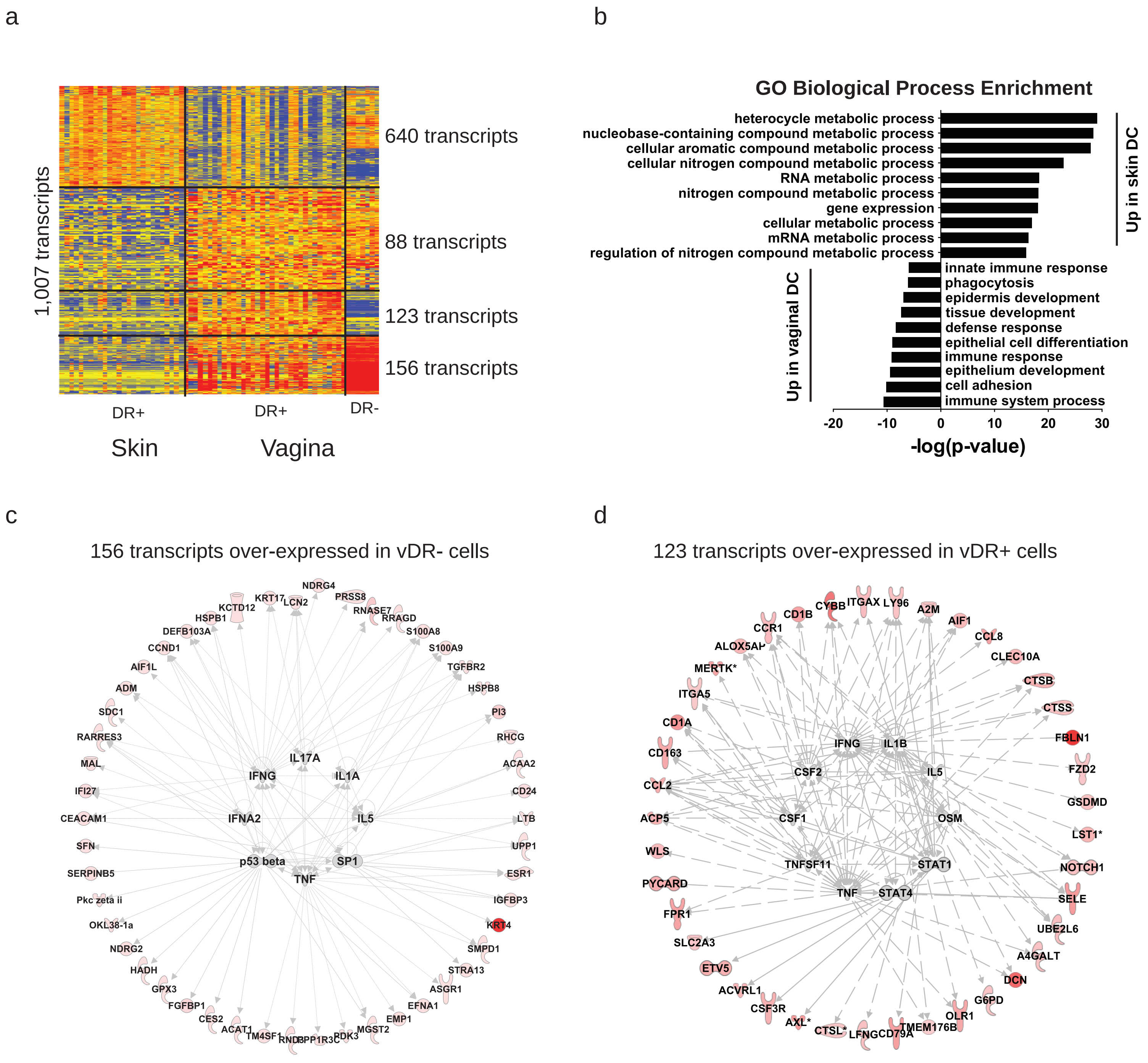

Figure S11 - Transcriptional comparison of skin vs vaginal DC subsets. **a.** 1,007 transcripts differentially expressed between skin and vaginal APC. HLA-DR- vaginal cells are also displayed. The data are normalized to the median of all APC (HLA-DR+) cells. **b.** Barchart representing GO biological process enrichment in skin and vaginal APC. **c.** IPA network analysis of the 156 transcripts over-expressed in vaginal HLA-DR- cells as compared to HLA-DR+ cells. **d.** IPA network analysis of the 123 transcripts over-expressed in vaginal HLA-DR+ cells as compared to HLA-DR- cells.
